# Integrated Omics-Based Discovery of Bioactive Halogenated Metabolites from the Deep-Sea *Streptomyces* sp. B188M101

**DOI:** 10.3390/md23090362

**Published:** 2025-09-19

**Authors:** Emmanuel Tope Oluwabusola, Stephen A. Jackson, Cristina Brunati, Stefanie Gackstatter, Hannah Vedder, Marianna Iorio, Gargee Chawande, Lekha Menon Margassery, Giang-Son Nguyen, David J. Clarke, Rainer Ebel, Marcel Jaspars, Alan D. W. Dobson

**Affiliations:** 1 Marine Biodiscovery Centre, Department of Chemistry, University of Aberdeen, Aberdeen AB24 3FX, UK; r.ebel@abdn.ac.uk (R.E.); m.jaspars@abdn.ac.uk (M.J.); 2 School of Microbiology, University College Cork, College Road, T12 K8AF Cork, Ireland; sjackson@ucc.ie (S.A.J.); gargee1997chawande@gmail.com (G.C.); l.margassery@ucc.ie (L.M.M.); david.clarke@ucc.ie (D.J.C.); a.dobson@ucc.ie (A.D.W.D.); 3 Sustainability Institute, University College Cork, Lee Road, T23 XE10 Cork, Ireland; 4 NAICONS, viale Ortles 22/4, 20139 Milan, Italy; cbrunati@naicons.com (C.B.); miorio@naicons.com (M.I.); 5 Interfaculty Institute of Biochemistry, University of Tübingen, 72076 Tübingen, Germany; stefanie.gackstatter@student.uni-tuebingen.de; 6 Department of Pharmacy, Philipps University Marburg, 35043 Marburg, Germany; hannahvedder@online.de; 7 Department of Biotechnology and Nanomedicine, SINTEF Industry, 7034 Trondheim, Norway; giangson.nguyen@sintef.no

**Keywords:** *Streptomyces* spp., antimicrobial activity, halogenated compound, *A. salmonicida*, lipopeptides, feature-based molecular networking, metaboanalyst

## Abstract

Using the one-strain-many-compounds (OSMAC) culturing approach, metabolomic studies, and bioassay-guided purification, we have isolated and characterised three new chlorinated natural products, agelolines B-D (**1**–**3**), together with two known compounds, ageloline A (**4**) and gausemycin A (**5**), which have been identified by high-resolution mass spectrometry and 1D and 2D NMR analyses. The preliminary evaluation of three small-scale extracts (M400, R358 and SGG) against the fish pathogen, *Aeromonas salmonicida* subsp. *achromogenes* KELDUR265-87, showed that the R358 extract displayed significant activity. Furthermore, the natural products (**1**–**5**) were evaluated against the fish pathogen *Aeromonas salmonicida* and human pathogens (*Stenotrophomonas maltophilia* L2125, *Staphylococcus aureus* ATCC6538P, and *S. pneumoniae* L44) using a serial dilution assay. Compound **3** displayed activity against *Staphylococcus aureus* ATCC6538P, *S. maltophilia* L2125, and *S. pneumoniae* L44 with MIC values of 6, 32, and 64 µg/mL, respectively. Interestingly, only gausemycin A (**5**) exhibited considerable inhibition against *A. salmonicida* with an MIC value of 32 µg/mL, and the activity increased by two-fold when supplemented with 0.45 mM calcium salt, while **2** and **4** showed moderate inhibition against *S. maltophilia* L2125. The biosynthetic pathways of compounds **1**–**4** were proposed. This is the first report of specific inhibition of *A. salmonicida* by **5**.

## 1. Introduction

Aquaculture is one of the fastest growing food production systems within the agrifood sector, with a 54% increase in production over the period 2010–2019, reaching levels of 94 million tonnes of seafood in 2022 [1], and it continues to be an important source of food for the world’s population [2]. The global aquaculture market was valued at USD 311.1 billion in 2023 and is projected to increase to USD 573.7 billion by 2035 at a 5.2% compound annual growth rate [3]. With the ongoing increase in food consumption globally, aquaculture will continue to play an important role as a food source and in doing so help with global food security, while addressing nutritional deficiencies and malnutrition challenges against the backdrop of an escalating world population [2]. One of the main challenges facing the aquaculture industry, particularly as it intensifies, is controlling a number of diseases caused by pathogenic bacteria, which can result in significant economic damage due to morbidity and death, which in 2014 was estimated to be in the region of USD 6 billion annually [4].

In this context, *Aeromonas salmonicida* (Family Aeromonadaceae, Phylum Pseudomonadota) is an important fish pathogen that constitutes the only known non-motile species in its genus, which comprises five subspecies which cause typical (subsp. *salmonicida*) or atypical (subsp. *achromogenes*, subsp. *masoucida*, subsp. *pectinolytica*, and subsp. *smithia*) furunculosis, soft tissue rot, fin rot, and haemorrhagic septicaemia in salmonid fish [5].

Antibiotics are commonly used in the aquaculture sector to treat fish during disease outbreaks, but their efficacy is often undermined due to the target bacteria developing antibiotic resistance resulting from the frequent use of these antibiotics and their subsequent persistence in the aquatic environment [6]. For example, the plasmidome of *A. salmonicida* is known to contain plasmids conferring resistance against chloramphenicol, spectinomycin, streptomycin, sulfonamides, tetracycline, mercury, and quaternary ammonium compounds [7,8]. A recent genomic-based study of *A. salmonicida* GMT3 further highlights the antibiotic resistance characteristics of the strain with genes potentially involved in resistance to quinolones, ampicillin, streptogramin, chloramphenicol, and polymyxin, together with genes encoding efflux pump systems [9]. *Aeromonas* spp. are also known to be able to transfer antibiotic resistance genes in different environmental ecosystems, and to survive as biofilms on biotic or abiotic surfaces, thereby facilitating the exchange in antimicrobial resistance genes within these ecosystems [10].

Thus, a more sustainable control strategy to protect fish from these disease outbreaks is required. In this respect the introduction or augmentation of beneficial microbes and their use in aquaculture as probiotics is considered an extremely promising approach to reduce fish diseases [11,12,13]. A stable and resilient microbiome is intimately involved in helping maintain overall good fish health by assisting the fish host in its defence against colonization and infection by pathogens. Specific microbes, or microbial consortia, and their metabolites are known to protect fish against these pathogens by enhancing stress resistance and immunity [12,14,15]. In particular a number of actinobacterial-based probiotics have been successfully employed in aquaculture systems, including *Streptomyces* strains [16], with for example *S. panacagri* and *S. floculus* being reported to display antagonistic activity against *Vibrio harveyi*, *V. parahaemolyticus*, and *V. vulnificus* [17].

Recent years have seen technological advances in areas such as high-throughput screening, genomics, and associated bioinformatics, as well as metabolomics and related chemoinformatics, which have accelerated the discovery, dereplication, and further characterisation of marine natural products [18,19]. The integration of these technologies, such as with the Paired Omics Data Platform [20] and with such tools as FERMO [21] and nanoRAPIDS [22], has streamlined the integration of complementary workflows, making detailed characterisations of the biosynthetic routes and structural characteristics of the chemical entities responsible for observed bioactivities much easier. The integration of high-throughput metabolomics and genomic sequencing has allowed for the targeted prioritisation of novel metabolites while linking this novel chemistry to their biosynthetic gene clusters from large libraries of bacteria [23,24] and fungi [25]. These studies have enabled the discovery of the anti-cancer peptide tambromycin, produced by *Streptomyces* spp. from a collection of actinomycetes, and also the discovery of 20 members of a new class of non-ribosomal peptides produced by *Micromonospora* spp. [24].

In an effort to identify a marine-derived antibiotic that may be useful in preventing the growth of various fish pathogens, we isolated *Streptomyces* sp. B188M101 from the deep-sea sponge *Lissodendoryx diversichela* [26]. In the fermentation process of the isolated bacterial strain, an OSMAC (one-strain-many-compounds) strategy was adopted to enhance production of unique secondary metabolites with the aim of expressing cryptic genes by altering the media composition of the culture broth [27,28,29,30]. The carbon and nitrogen sources, including the salt content of the growth media composition, were considered in particular in the selection of the three media (R358, M400, and SGG) in the fermentation process. We report here on the use of metabolomic studies and bioassay-guided isolation in the identification of a unique and bioactive extract that led to the discovery of three new chlorinated natural products and the first report of a gausemycin-type compound with growth inhibition activity against the gram-negative fish pathogen, *A. salmonicida*.

## 2. Results and Discussion

### 2.1. Non-Targeted Multivariate Analysis of OSMAC Extracts

*Streptomyces* strain B188M101 was fermented in different growth media (SGG, M400, and R358) and in three biological replicates together with their respective process blanks. An untargeted metabolomic study was employed to determine the best culture media suitable for the growth and elicitation of uncommon bioactive secondary metabolites from the actinobacterial strain [31,32]. Hence, the extracts obtained were subjected to comparative metabolomic studies to uncover extracts with unique chemical diversity and specificity. Understanding the chemical space in an LC-MS dataset of a microorganism fermented under different conditions requires specialized unsupervised learning techniques: principal component analysis (PCA) [33,34], one-factor statistical analysis, including ANOVA, and the use of a Hierarchical Clustering Dendrogram [33,35].

The centroided raw data of positive mode ionisation of each extract generated from high-resolution Orbitrap IQ-X mass spectroscopy (Thermo Scientific, Waltham, MA, USA) was converted to the mzML format and further pre-processed on the MZmine software version 4.2.0 to produce feature peak lists and intensity data required for metaboAnalyst software version 6.0. Precursor mass ions from the UPLC solvent and media blanks were removed from the sample data before downstream analysis to prevent distortion of the results.

Of the 1508 molecular ion peaks obtained with an average of 167.8 peaks per sample, a total of 63.45% missing values were detected, resulting in a wide variability of metabolites amongst the extract datasets to be compared; this is a common occurrence in untargeted LC-MS data, in this case, due to the chemical diversity.

**Figure 1:**
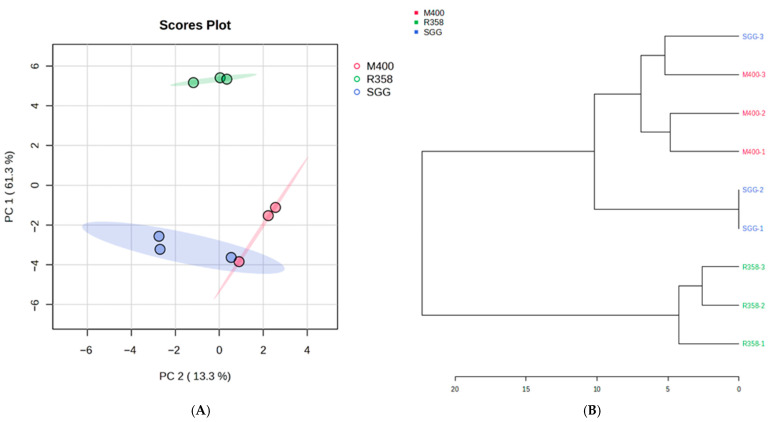
(**A**) Principal Component Analysis (PCA) score plot. Pink circles correspond to the M400 extract; green circles correspond to the R358 extract; navy blue circles correspond to the SGG extract. (**B**) Hierarchical Clustering Dendrogram (samples were clustered using the Ward method and Euclidean distance metric): the same colour representation as (**A**) using squares.

The principal component analysis (PCA) plot is displayed in Figure 1A clearly shows the relationship between the three extracts based on chemical profile similarity [36]. The first principal component (PC1) that covers the largest variance in the dataset followed by the second principal component (PC2), orthogonal to PC1 with 61.3% and 13.3%, confirmed the extent of the shared secondary metabolite distribution amongst the three extracts. However, the R358 extract was an outlier compared to the SGG and M400 extracts, being located at a distant quadrant from the respective two clusters.

The results show a low *p*-value of 0.0045 in comparison with the software default setting *p*-value of 0.05, indicating strong evidence that the variation is statistically significant. The extract datasets were further analysed using a Hierarchical Clustering Dendrogram, which as previously mentioned is another unsupervised technique used to perform exploratory data analysis (EDA) that allows for in-depth understanding of skewed data by identifying the main features which can be visualised in dendrogram formation, that is, a binary merge tree [37,38,39]. The R358 metabolites appear as a distinct cluster from the other two extract metabolites, supporting the variation observed in the two-dimensional space established by the PCA score plot (Figure 1A). Thus, it appears that the *Streptomyces* B188M101 produced shared groups of metabolites when fermented in SGG and M400 media.

### 2.2. Preliminary Disc Diffusion Assays

**Figure 2:**
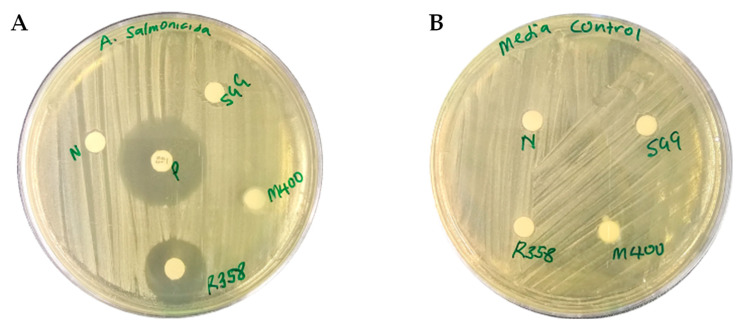
(**A**) Disc diffusion assay showing zone of inhibition of the M400, R358, and SGG extracts and gentamicin antibiotic (P) against *A. salmonicida*. (**B**) Media control of M400, R358, SGG, and dried methanol discs as negative control (N).

The M400, R358, and SGG extracts were screened against *Aeromonas salmonicida*, using a disc diffusion assay following a previously described method [40]. Following 24 h of incubation, significant inhibition was observed from the R358 extract at a concentration of 8 mg/mL with a zone of inhibition of 20 mm which was comparable to the 30 mm zone of inhibition observed from the broad-spectrum antibiotic gentamicin tested at 10 µg (Figure 2A). No activity was observed from the M400 or SGG extracts (Figure 2A) or from the media control discs (Figure 2B).

### 2.3. Chemoinformatic Feature-Based Molecular Networking and Database Dereplication Analysis

**Figure 3:**
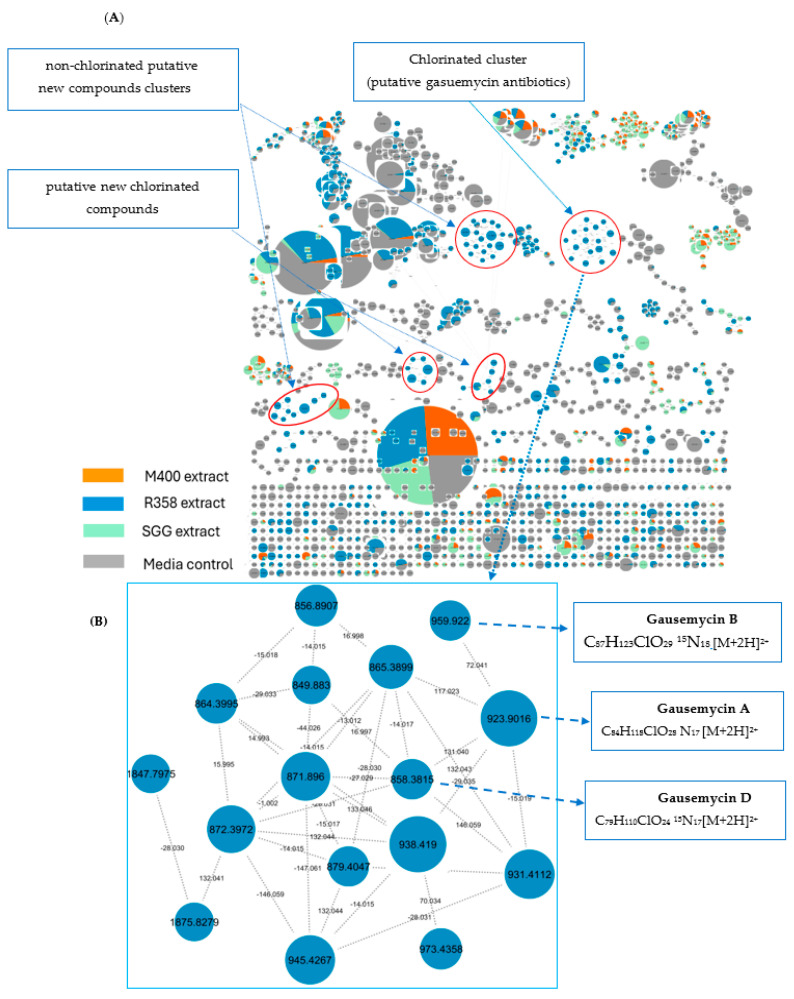
Global molecular network of the marine-derived *Streptomyces* B188M101 extracts (M400, R358 and SGG). (**A**) Overall molecular network of M400, R358, SGG, and media control extracts; red circles highlight three unique chlorinated clusters. (**B**) Clearer view of molecular mass clusters of putative new chlorinated compounds including the known gausemycin antibiotics.

Both the one-factor-statistical analysis and disc diffusion assay with *A. salmonicida* established that the R358 extract contained bioactive secondary metabolites. Thus, we subsequently subjected the LC-MS/MS ESI-Orbitrap data of the three extracts to feature-based molecular networking (FBMN) [41] analysis. FBMN is a computational method that bridges mass spectrometry data processing tools for LC-MS/MS and molecular networking analysis on the Global Natural Products Social Networking (GNPS) site and was employed here to investigate the molecular ecosystem and annotate new compounds in the crude extracts SGG, R358, and M400 by visualising the entire integrated features of the metabolomes. This type of bioinformatic technique mainly utilises the mass fragmentation information of the node (mass peak) in each dataset to determine their spectrum and feature similarity, hence, organising the family of molecules in clusters. The raw files of the Orbitrap MS were converted to mzML format using MSConvert software [42], followed by pre-processing with MZmine 4.2.0 version to create a quantitative MS1 file and MGF files of fragment ions [43]. The results of the analysis are publicly available at https://gnps.ucsd.edu/ProteoSAFe/result.jsp?task=a5069300445a407d90c4b80162125c2c&view=download_cytoscape_data, accessed on 23 May 2025. Analysis of the GNPS data on Cytoscape 3.10.0 [44,45] showed 596 molecular features arranged in clusters and singletons after removal of features (1285 nodes) belonging to SGG, R358, and M400 media controls (Figure 3A).

**Figure 4:**
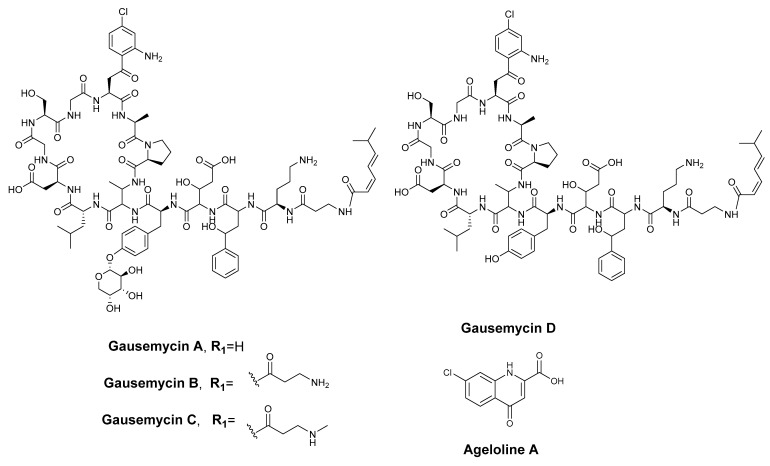
The known compounds identified in the R358 extract of *Streptomyces* B188M101.

It is worth noting that 15 of the 31 molecular clusters that contained two or more features in the molecular network were specific to the R358 extract. The remaining clusters consisted of features found in either M400 or SGG extracts, further corroborating the chemical variation observed using the multivariance one-factor analysis (Figure 1). The natural product database search on the GNPS website (GNPS; http://gnps.ucsd.edu; accessed on 14 June 2025) of the identified features detected in the molecular network clusters (Figure 3A) did not show any potential match. Hence, manual dereplication processing using the Thermo Scientific FreeStyle™ 1.8 SP2 QF1 (Thermo Scientific, Waltham, MA, USA) was utilised to analyse the MS spectrum of the R358 extract and chemically profile the precursor ions at *m*/*z* for each base peak organised according to their retention time. Also, molecular formulae in accordance with the mass isotopic pattern matching [46], theoretical mass, and mass error within 3 ppm mass accuracy were established [47,48]. The dereplication of the distinctive clusters gave 23 new molecular features whose elemental compositions were consistent with chlorinated isotopic signatures and intense doubly charged base peaks (Table S1 and Figure 3B). In addition, 24 putative new non-halogenated molecules (see Table S1) and 4 known gausemycins A–D derivatives [49,50] and ageloline A [51] (see Table S1 and Figure 4) were annotated following an assessment of both the accurate exact masses and the corresponding molecular formulae on the natural product databases in combination with MS/MS fragmentation analysis.

Earlier work on these natural lipoglycopeptides led to the isolation of gausemycin A and B from the fermentation broth of *Streptomyces* sp. INA-Ac-5812 [49]. Other minor analogues, C–F, were subsequently isolated and structurally elucidated from the same bacterial strain [50]. According to the high-resolution Orbitrap IQ-X ESI-MS, doubly charged intense precursor ion peaks at *m*/*z* 923.9016, 959.4201, 966.4280, and 857.8801(M+2H)^2+^ corresponding to molecular formulae of C_84_H_118_ClO_28_N_17_, C_87_H_123_ClO_29_N_18_, C_88_H_125_ClO_29_N_18_, and C_79_H_110_ClO_24_N_17_, were attributed, respectively, to gausemycins A–D [49,50]. Their structural scaffold consisted of a cyclic core and a tail which were made up of amino acids sequences and non-proteogenic amino acids, 2-amino-4-hydroxy-4-phenylbutyric acid (Ahpb), 2,3-diaminobutyric acid (Dab), chlorinated L-4-chlorokynurenine (ClKyn), and sugar moieties (Figure 4).

A detailed MS^2^ analysis of gausemycin A (*m*/*z* 1846.7936, [M+H]^+^) was performed using high-energy collisional dissociation (HCD), selected due to the presence of a cyclic core that is less amenable to fragmentation via low-energy collision-induced dissociation (CID). The resulting fragmentation spectra revealed characteristic b- and y-ions corresponding to the N- and C-terminal regions, respectively. Fragment ions of both lower and higher *m*/*z* values were observed, representing sequences from the linear tail region as well as the cyclic core structure of gausemycin A. A detailed explanation as to the MS/MS fragmentation of gausemycin A is provided in the supplementary information (Figures S1 and S2).

Gausemycin B and C whose molecular ions were deduced as *m*/*z* 1917.8325 and 1931.8458, with respective intense doubly charge ions at 966.42587 and 959.4201[M+2H]^+2^, differ from gausemycin A by 71.0487 and 87.0487 Da, confirming the addition of β-alanine and methyl-β-alanine as previously reported [50] and corroborated by the MS/MS fragmentation analysis (Figures S3 and S4). The absence of sugar moieties in the MS/MS fragments of gausemycin D (*m*/*z* 1714.7521 [M+H]^+1^) showed it was consistent with the reported data [50]. The variation amongst known compound structures of gausemycins has been established to exist in the fatty acid chain and the attachment of amino acid building blocks on the amine functionality of ornithine at the tail region [49,50]. For instance, the molecular ions observed at *m*/*z* 864.8885 were dereplicated as putative new congeners of gausemycin D, corresponding to the deduced molecular formula C_80_H_112_ClO_24_N_17_ (Table S1 and Figure S5). The mass difference of 14.0176 Da relative to gausemycin D, supported by MS/MS experiments, suggests the direct incorporation of monomethyl on the ornithine side-chain amine group (Figure S6). This interpretation is further corroborated by the clustering of molecular ions in the molecular networking analysis (Figure 3). Additionally, fragment ion analysis of doubly charged molecular ions at *m*/*z* 930.9088 and 973.4351, corresponding to molecular formulas C_85_H_120_ClO_28_ N_17_ and C_89_H_127_ClO_29_N_18_ (Table S1, and Figures S7 and S9), revealed structural features consistent with gausemycin A. However, these respective analogues exhibited additional modifications, specifically monomethyl and dimethyl-β-alanine substituents on the ornithine amine group (Figures S8 and S10). Recent findings provide mechanistic insights into the biosynthetic pathway by which non-standard residues, such as ornithine, are methylated through methyltransferase domains embedded within non-ribosomal peptide synthetase (NRPS) systems [52,53,54]. While this dereplication process has revealed the presence of several previously uncharacterised molecules within the R358 extract, the present study is now directed towards their targeted isolation and comprehensive structural characterisation.

### 2.4. Isolation and Structure Elucidation of New Chlorinated Compounds

**Figure 5:**
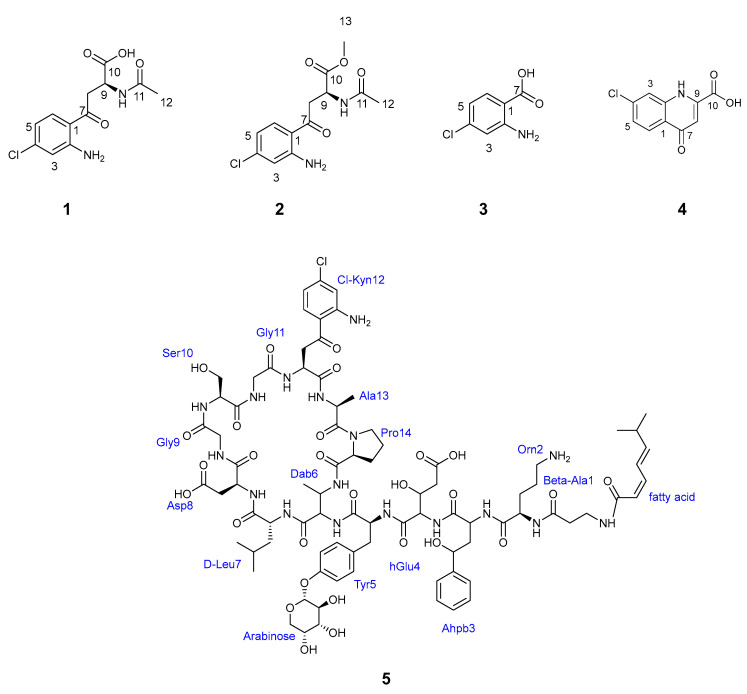
Structures of agelolines B–D (**1**–**3**), ageloline A (**4**), and gausemycin A (**5**).

A large-scale culture (8L) of *Streptomyces* sp. B188M101 was grown on R358 medium in a closed system shaker at 28 °C for 10 days, yielding 16.45 g of combined extract. The extract was fractionated following a modified Kupchan solvent partitioning method [55], and upon dereplication analysis, subsequent purification of the butanol fraction resulted in the isolation of three new chlorinated compounds, designated as agelolines B–D (**1**–**3**), and two known compounds, ageloline A (**4**) [51] and gausemycin A (**5**) [49,50] (Figure 5), as outlined in the materials and methods section.

**Figure 6:**
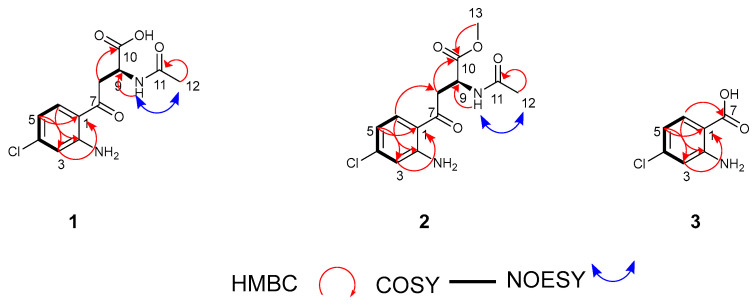
Key HMBC, ^1^H—^1^H COSY and NOESY correlations of compounds **1**–**3**.

Compound **1** was isolated as yellowish powder. The high-resolution electron spray ionisation Orbitrap mass spectroscopy of **1** revealed an isotopic pattern indicating the presence of a chlorinated atom at *m*/*z* 285.0638/287.0607 (M+H, Δ + 0.5 ppm), and assigned the molecular formula C_12_H_14_ClN_2_O_4_, requiring 7 degrees of unsaturation (see Figure S11). The 1D and 2D NMR data shows the presence of signals consistent with three sp^2^ aromatic methines, one sp^3^ aliphatic methine, one sp^3^ methylene, one sp^3^ methyl, and six sp^2^ quaternary carbons (see Table 1 and Figures S12–S14). Detailed analysis of ^1^H NMR chemical shifts, coupling constants, and COSY data suggested the presence of a 1,2,4-trisubstituted aromatic ring exhibiting coupling from H-5 (δ_H_ 6.56, *J* = 8.7, 2.2) to both H-6 (δ_H_ 7.72, *J* = 8.7) and H-3 (δ_H_ 6.77, *J* = 2.2). The assignment of aromatic substructure, including the quaternary carbons to positions C-1 (δ_C_ 116.7), C-2 (δ_C_ 153.3) and C-4 (δ_C_ 141.0), were based on the chemical shifts and strong HMBC correlation from H-5 to C-1 and C-3 (δ_C_ 116.9), from H-6 to C-2 and C-4, and from H-3 to C-1 and C-5 (*δ_C_* 115.9) (Figure 6 and Figure S15 and Table S2). The downfield chemical shifts at δ_C_ 153.3 and 141.0 were due to the presence of primary amine and chlorine substituents at C-2 and C-4, respectively.

The aliphatic substructure of **1** was constructed using the COSY spectrum, showing a spin system evident by the coupling between H-9 (δ_H_ 4.89, m) and the diastereotopic protons H_2_-8 (δ_H_ 3.54, *J* = 17.3, 6.3); 3.45, *J* = 16.9, 6.3) and a cross-peak HMBC correlation from H_2_-8 to C-6 (δ_C_ 133.6), C-7 (δ_C_ 198.1), C-9 (δ_C_ 49.3), and C-10 (δ_C_ 173.1, carboxylic acid unit), and from H-9 to C-7, C-8 (δ_C_ 41.2), C-10, and to the acetyl carbonyl carbon C-11 (δ_C_ 172.9). When the NMR data for compound **1** was recorded in deuterated DMSO (Figure S16), ^1^H-^1^H coupling was observed from H-9 to the amide proton (δ_H_ 8.05, NH), which supported the acetyl group (C-11; δ_C_ 21.8, C-12); this was corroborated by a NOESY correlation from the amide proton to methyl H_3_-12 (δ_H_ 1.97) (Figure S17) and HMBC correlation of singlet methyl protons H_3_-12 to C-11 (Figure 6). Hence, the complete structure of compound **1**, designated as ageloline B, confirmed it to be structurally related to the previously characterised antichlamydial quinolone ageloline A isolated from *Streptomyces* sp. SBT345 [51].

Compound **2** was isolated as a light yellowish powder, and its molecular formula was obtained from the ESI Orbitrap MS with a characteristic chlorinated isotopic cluster at *m*/*z* 299.0795/301.0764 (M+H, Δ +0.5 ppm) with 7 degrees of unsaturation (Figure S18). The complete 1D and 2D NMR data (see Table 1 and Table S3 and Figures S19–S22) confirmed **2** to be similar to **1** except for the presence in its spectra of signals corresponding to a methoxy group. A cross-peak HMBC correlation from the methyl proton signal (δ_H_ 3.72, H_3_-13) to carbonyl carbon C-10 supported the attachment (Figure 6). To assess whether compound **2** was an artefact resulting from spontaneous methylation induced by methanol during the extraction process—despite its detection in the LC-MS profile of the crude extract—a 50 mL culture was prepared and extracted using *sec*-butanol instead of methanol. The resulting extract was analysed via ESI-Orbitrap MS. The LC-MS chromatogram confirmed the presence of both compounds **1** and **2**, with base peaks observed at retention times of 9.72 and 10.59 min, respectively (see Figure S23). Notably, methanol was excluded from all subsequent extraction, fractionation, and purification steps.

The absolute configurations of compounds **1** and **2** were logically assigned as **L** based on extensive prior work demonstrating that 4-chloro-L-kynurenine (L-4-ClKyn)—a core substructure of both compounds—is an integral component of natural peptide antibiotics [56]. This non-proteinogenic, modified amino acid has been identified as part of the building block sequence in lipopeptide antibiotics such as taromycin-type compounds and lipoglycopeptide antibiotics [49,50,56], and is also reported in the present study. Further support for the L-configuration was provided by optical rotation measurements, which yielded a negative value, consistent with the expected behaviour of L-4-ClKyn derivatives and other L-amino acids. Based on this conclusion, structure **2** was determined and named ageloline C.

Ageloline D (**3**) was isolated as a reddish yellow oil, with a molecular formula of C_10_H_7_ClO_3_N deduced from the molecular ion in the positive mode ESI Orbitrap MS at *m*/*z* 172.0163 (M+H, Δ + 1.3 ppm) corresponding to 5 degrees of unsaturation (Figure S24). The ^1^H NMR spectrum of **3** showed coupling patterns suggestive of the same 1,2,4-trisubstituted aromatic ring found in **1** and **2**. The carboxyl functionality was placed at C-7 (δ_C_ 170.8) due to the downfield chemical shift and a strong HMBC cross peak from H-6. The 1D and 2D NMR spectra data (see Table 1 and Table S4 and Figures S25–S28), together with the mass spectrometry analysis, established **3** as 2-amino-4-chlorobenzoic acid—a previously unreported natural product and a structural motif shared by **1** and **2**.

Compound **4** was obtained as a yellowish powder which displayed a protonated ion, [M+H]^+^, in the positive mode HRESIMS at *m*/*z* 224.0109, from which the molecular formula of **4** was deduced as C_10_H_7_ClO_3_N (Δ: 0 ppm, 8 degrees of unsaturation, calcd. for C_10_H_7_ClO_3_N, 224.0109) (Figure S29). The comparison of 1D, 2D NMR data as well as the MS data of 4 (see Table S5, Figures S30–S33) with previously reported experimental data for ageloline A confirmed its identity [51].

Compound **5** was isolated as a white solid and peaks in its Orbitrap HR-ESI-MS spectrum at *m*/*z* 1846.7936 with a characteristic chlorinated pattern and a doubly charged ion at *m*/*z* 923.9016, both in agreement with a molecular formula of C_84_H_118_ClO_28_ N_17_ and 34 degrees of unsaturation (Figure S34). The structure of **5** was determined from full NMR spectroscopy data measured in DMSO-d6 at 318 K, and the MS/MS fragmentation pattern as suggested in the chemical dereplication section (see Tables S6 and S7 and Figures S1, S2 and S35–S37). The absolute configurations of all the proteinogenic amino acids in **5** were established by the Marfey experiment, which was performed following chromatographic analysis by reverse-phase analytical HPLC with a wavelength of 340 nm of the hydrolysate and the standard derivatised amino acids (see Table S8). The structure of **5** was confirmed as gausemycin A after a thorough check with the literature data [49].

### 2.5. Genomics of B188M101

**Figure 7:**
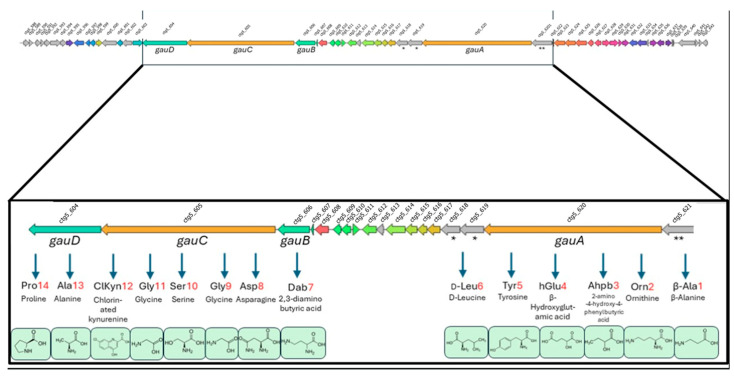
Structure of the biosynthetic gene cluster, from *Streptomyces* sp. B188M101, presumed to produce gausemycins. The substrates of the NRPS modules are indicated. * Two ORFS (ctg_618 and ctg_619) are homologous to a single NRPS module from the gausemycin BGC from *Streptomyces* sp. INA-Ac-5812 [49]. These ORFs are predicted to incorporate d-Leucine into the polypeptide. ** An ORF not annotated by antiSMASH to be part of the *gauA* NRPS is homologous to a NRPS module in the *Streptomyces* sp. INA-Ac-5812, within *gauA*, and incorporates β-alanine into the polypeptide.

*Streptomyces* sp. B188M101 was taxonomically classified as a member of the *S. fimicarius* subgroup (Figure S38). The identification and characterisation of the chlorinated compounds (**1**–**5**) allowed us to explore the B188M101 genome and identify a putative halogenase gene, namely a tryptophan-7-halogenase encoding gene within a 111.8 Kb biosynthetic gene cluster (BGC). The BGC was annotated by antiSMASH version 7.0 as a NRPS/betalactone hybrid cluster comprising 56 ORFs (Table S9). The BGCs most closely related to the NRPS/betalactone cluster from *Streptomyes* sp. B188M101 are those which produce compound **5** (gausemycin A) and gausemycin B from *Streptomyces* sp. INA-Ac-5812 [49], the cadasides- [57], and malacidins- [58] producing clusters from soil metagenome cosmid libraries, and the friulimicins-producing cluster from *Actinoplanes friuliensis* [59] (Figure S39). The products of these clusters are cyclic lipopeptides or lipoglycopeptides with antimicrobial activities against Gram-positive bacteria and have been reported to have Ca^2+^ dependency. Based on the biosynthetic gene cluster composition, the heterocylic polypeptide produced from the *Streptomyces* sp. B188M101 is identical to that reported for gausemycin A produced by *Streptomyces* sp. INA-Ac-5812 (βAla1-Orn2-Ahpb3-hGlu4-Tyr5-Dab6-D-Leu7-Asp8-Gly9-Ser10-Gly11-ClKyn12-Ala13-Pro14) [49] (Figure 7).

Unlike the gausemycins, which comprise 9 amino acid residues in the heterocycle, and exocyclic 5 amino acids—including non-proteogenic components—cadasides comprise 9 amino acids in the heterocycle with 4 exocyclic residues, while friulimicins comprise 10 amino acids in the heterocycle with one exocyclic residue. While the BGC of *Streptomyces* sp. B188M101 hosts 56 ORFs (Table S9) spanning 111.8 Kb, the gausemycins cluster of *Streptomyces* sp. INA-Ac-5812 comprises 68 ORFs and is 121.5 Kb in length. A comparison of the two clusters reveals that 35 ORFs are homologous with 34.6–99.1% amino acid identities (Figure S33 and Table S10). While the *gauA* NRPS of *Streptomyces* sp. INA-Ac-5812 (Accession no. QWT72279.1) is annotated as comprising 8448 amino acids, incorporating 6 moieties into the polypeptide, that of *Streptomyces* sp. B188M101 (ORF 33: Figure 7; Supplementary Table S9) is annotated as a 5910 amino acid NRPS incorporating 4 amino acids into the peptide. However, ORFs 31, 32, and 34 are annotated as separate NRPS genes, and along with ORF 33, they are predicted to produce an identical polypeptide to that of *Streptomyces* sp. INA-Ac-5812. The homologues of *gauB*, *gauC*, and *gauD* in the genome of *Streptomyces* sp. B188M101 share amino acid sequence identities of 93.9%, 89.8%, and 92.4%, respectively (Supplementary Table S10).

Notwithstanding that the orientation of *Streptomyces* sp. B188M101 BGC and *Streptomyces* sp. INA-Ac-5812 is different, ORF 9 to ORF 50 in the former is largely homologous to ORF 11 to ORF 44 in the latter. The main differences are the insertions of genes encoding a pyridoxal-phosphate dependent enzyme; cysteine synthase (ORF 13), an argininosuccinate lyase/adenylosuccinate lyase (ORF 15), and a methyltransferase (ORF 26) in the BGC of *Streptomyces* sp. B188M101, which are absent from the gene cluster of *Streptomyces* sp. INA-Ac-5812, while the latter cluster hosts a flavin reductase family protein encoding gene (ORF 33) and a cation/H(+) antiporter encoding gene (ORF 45) not present in the cluster of *Streptomyces* sp. B188M101. A biosynthesis schema has been proposed by Tyurin and colleagues [49], with a similar pathway likely to be present in *Streptomyces* sp. B188M101. In some cases, single BGCs are known to produce more than one metabolite [60] and differences between the gausemycin BGC from *Streptomyces* sp. INA-Ac-5812 and the NRPS/betalactone cluster from *Streptomyces* sp. B188M101 may result in the production of different minor metabolites. As highlighted in Figure 3, while a number of new gausemycin analogues have been identified in the metabolome of *Streptomyces* sp. B188M101 as visualised through the molecular network annotation, attempts to isolate these new analogues have to date been unsuccessful. Interestingly, ORF 51 in the BGC of *Streptomyces* sp. B188M101 encodes an IS3 family transposase, suggesting the potential for horizontal gene transfer of part or all of this cluster.

### 2.6. The Proposed Biosynthesis of Ageloline A-D (***1***–***4***)

**Figure 8:**
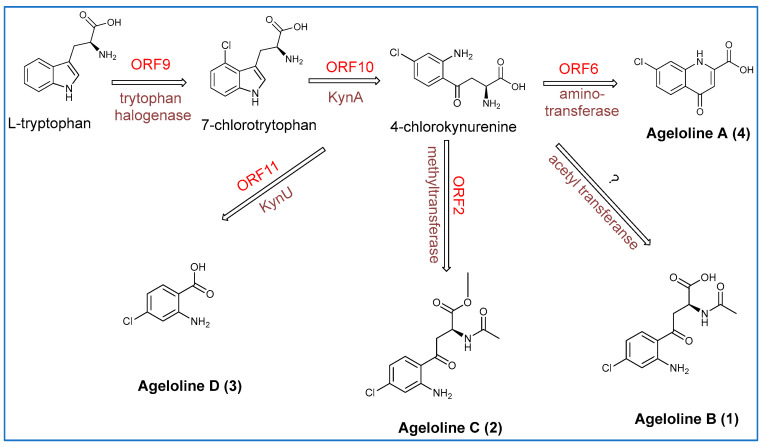
Proposed biosynthetic scheme for ageloline A–D (**1**–**4**).

The agelolines A–D are natural derivatives of 7-chlorokynurenic acid, partially biosynthesized from the gausemycin biosynthetic gene cluster (BGC), as evidenced by their shared chlorinated moiety. Ageloline A is proposed to originate from the kynurenine pathway, beginning with halogenation of L-tryptophan by ORF9, followed by conversion to 7-chlorokynurenine via ORF10, annotated as tryptophan 2,3-dioxygenase (antiSMASH) and *kynA* (PROKKA version 1.14.5). A final transamination step, likely catalysed by a kynurenine aminotransferase (KAT), completes the biosynthesis (See Table S10 and Figure 8). ORF6, annotated as a histidinol-phosphate aminotransferase (*hisC*), may fulfil this role despite histidinols not being typical KAT substrates [61].

Agelolines B and C follow similar initial steps, diverging through distinct tailoring reactions. Ageloline B requires acetylation of 7-chlorokynurenine, yet no acetyltransferase is encoded within or near the BGC, which suggests the likely involvement of a distal genomic element; the closest candidate lies 84.6 Kb upstream, annotated as an L-methionine sulfoximine/sulfone acetyltransferase. Ageloline C is methylated via a methyltransferase encoded by ORF2, which is absent in the gausemycin-producing *Streptomyces* sp. INA-Ac-5812, suggesting strain-specific biosynthetic capabilities. Ageloline D, a chlorinated anthranilic acid, is likely formed via kynureninase (*kynU*) activity on chlorinated L-kynurenine. ORF11, annotated as an α-/β-hydrolase (antiSMASH) and *kynU* (PROKKA), is presumed to catalyse this conversion (Table S10 and Figure 8).

Together, these findings suggest that agelolines are derived from a common halogenated intermediate, with diversification driven by distal or strain-specific tailoring enzymes. Annotation discrepancies between antiSMASH and PROKKA highlight the need for functional validation, while the absence of key enzymes within the BGC points to broader genomic involvement in ageloline biosynthesis.

### 2.7. Biological Evaluation of Compounds ***1***–***5***

Compounds **1**–**5** were initially evaluated for their antibacterial activity against the aquaculture fish pathogen *A. salmonicida* using the enzymatic cell-based assay previously described. Only compound **5** exhibited considerable activity at an MIC value of 32 µg/mL (see Figure S40A). However, the screening platform was expanded to accommodate human pathogens, including *S. aureus* ATCC6538P, *S. aureus* GISA L3797, *S. pneumoniae* L44, *S. maltophilia* L2125, *P. aeruginosa* PA01, and *E. coli* ΔtolC L4242. Compound **3** showed significant to moderate inhibition against *Staphylococcus aureus* ATCC6538P, *S. maltophilia* L2125, and *S. pneumoniae* L44 with MIC values of 6, 32, and 64 µg/mL, respectively, while moderate inhibition against *S. maltophilia* L2125 was observed for **2** and **4** (Table 2; Figures S40 and S41).

A recent discovery reported that the bioactivity of compound **5** against methicillin-resistant *S. aureus* increases with the addition of calcium to the cell culture—an observation that led to a successful patent application [62]. Our experiment confirmed this finding and extended it beyond Gram-positive *S. aureus* to Gram-negative *A. salmonicida*, showing an 8- and 2-fold increase in activity with MIC values of 4 µg/mL and 16 µg/mL, respectively, when the assay cell culture broths were supplemented with 0.45 mM of calcium chloride. In contrast, increasing the calcium concentration to 20 mM yielded no further enhancement of the activity of **5** against any of the tested pathogens (Table 3 and Figure S42).

It is quite unusual for cyclic glycolipopeptides to display activity against Gram-negative bacteria, as most antibiotics of these structural scaffolds are prevented from crossing the extra outer membrane (OM) barrier consisting of lipopolysaccharides (LPS) [63,64].

The outer layer is negatively charged and creates a permeability barrier that repels most antibiotics partly due to their large size [63,64,65]. It was found that gausemycins are species-specific, as a wide range of antimicrobial inhibitions were observed when tested against a panel of Gram-positive bacteria [50], indicating multiple molecular targets, including membrane disruption in a Ca^2+^-dependent manner and channel-forming activity at a higher concentration of calcium ions [49,50].

This study presents the first report of compound **5** exhibiting species-specific inhibition of *Aeromonas salmonicida* while other Gram-negative strains—including *S. maltophilia* L2125, *P. aeruginosa* PA01, and *E. coli* ΔtolC L4242—remained unaffected, regardless of calcium salt supplementation. This selective activity may be attributed to the distinctive structural and antigenic properties of the surface layers (S-layer) of the *A. salmonicida* outer membrane, which differ substantially from those of other Gram-negative bacteria [66,67].

## 3. Materials and Methods

### 3.1. General Experimental Procedures

UV spectra were recorded on a Thermo Scientific UPLC system coupled to a photodiode array detector (Thermo Scientific, Waltham, MA, USA). Optical rotations were measured on an ADP 410 digital polarimeter (Bellingham + Stanley Ltd., Nottingham, UK). NMR spectra were recorded on Bruker AVANCE III spectrometers at 600 MHz (Bruker Corporation, Billerica, MA, USA). The Thermo Instruments UPLC system was connected to the Orbitrap IQ-X MS (Thermo Scientific, San Jose, CA, USA), equipped with heated electrospray ionisation (H-ESI). The Orbitrap was used to analyse the biological replicate samples under the following operating conditions: capillary temperature 275 °C, auxiliary gas flow rate 10 arbitrary units, sheath gas flow rate 50 arbitrary units, static gas mode, sweet gas (Arb) 1.0, ion transfer tube temperature 275 °C, vaporiser temperature 350 °C, capillary positive ion spray voltage 3.5k, and a lock mass correction set at RunStart EASY-IC. The full MS was set with a scan mass range of 150–2000 *m*/*z*, Orbitrap resolution at 120,000, RF Lens at 35%, absolute AGC target at 4.000 × 105, maximum injection time mode at Auto, and data type set to centroid with the data-dependent acquisition mode set at Cycle Time (Top Speed). The assisted HCD (high-energy collisional dissociation) energy was set at 30%, with an activation time of 10 milliseconds, and the Orbitrap resolution was set at 60,000 for the MS/MS fragmentation step.

The LC system consists of a diode array detector (DAD) with a variable wavelength detection range of 200–800 nm, and an analytical Kinetex 2.6 µm EVO C18 column (100 Å, 100 × 2.1 mm). A mobile phase gradient of 0.1% formic acid in LC-MS grade H_2_O: acetonitrile from 5 to 95% in 13 min, and isocratic elution at 95% for 5 min at a flow rate of 300 µL/min with MS acquisition for 17 min, was employed in the UPLC and MS system.

### 3.2. Microbial Isolation and Identification

*Streptomyces* sp. B188M101 was isolated from the deep-sea sponge *Lissodendoryx diversichela* as previously described [26]. The genome of *Streptomyces* sp. B188M101 was sequenced using PacBio Sequel II following library preparation using SMRTbell Express Template Prep Kit 2.0, by Novogene Co. Ltd., Cambridge, UK. The genome was assembled using SPAdes version 3.11.1 [68]. The assembled genome was annotated using Prokka v1.14.5 [69]. Taxonomic assignment and phylogenetic analysis were performed using the Genome Taxonomy Database (GTDB-Tk) v2.3.2 [70], which was implemented on the KBase web platform [70,71]. Biosynthetic gene clusters were identified using antiSMASH v 7.0 [72], cblaster [73], and clinker [74].

### 3.3. Small Fermentation of Streptomyces sp. B188M101 Using an OSMAC Approach

A seed culture of *Streptomyces* sp. B188M101 was prepared by inoculating an autoclaved 50 mL ISP2 medium (malt extract 10 g, yeast extract 4 g, and glucose 4 g in 1 L of milli-Q H_2_O, pH adjusted to 7.2) in a 250 mL Erlenmeyer baffled flask with a pure colony of the bacterial strain from an agar plate. Incubation of the seed culture lasted for 72 h at 28 °C and 180 rpm in a closed-system rotary shaker (SciQuip, Rotherham, UK). Following an OSMAC approach, three biological replicates of 200 mL media culture consisting of SGG-(a-c) [glycerol 10 g, soluble starch 10 g, corn steep powder 2.5 g, yeast extract 2 g, peptone 5 g, CaCO_3_ 3 g, NaCl 1 g, in 1 L H_2_O, pH adjusted to 7.3], M400-(a-c) [1% glucose, 0.03% meat extract, 0.3% peptone, 2% soluble starch, 0.5% yeast extract, 0.3% CaCO_3_), and R358-(a-c)[0.2% peptone, 0.4% yeast, 1% soluble starch, 4% sea salt, 5 mL/L of an 8 g/L FeSO_4_∙7H_2_O stock solution, 5 mL/L of an 8 g/L KBr stock solution, 4% sigma sea salts] in 1L Erlenmeyer baffled flasks were further inoculated with 2 mL of the seed culture and incubated for 10 days under the same shaking conditions as the seed culture. Alongside the bacterial cultures, the blank controls consisting of 200 mL of the three uncultured media were prepared to remove interfering metabolites from the culture media. After fermentation, an autoclaved Dianion HP-20 absorbent resin (12 g/200 mL) was added to each of the culture broths and allowed to shake for another 7 h. The HP-20 adsorbent resins were filtered under a vacuum, and each culture broth resin and the respective process blank medium was placed in a separate, cleaned 1 L Erlenmeyer flask containing 200 mL of 100% methanol to macerate for 24 h. Complete extraction of the biomass was achieved after subsequent cycles of extraction with MeOH (100 mL) and ethyl acetate (100 mL). The combined extract was concentrated under reduced pressure with a vacuum rotatory evaporator (IKA VACSTAR digital, Staufen im Breisgau, Germany) and later dried completely in a nitrogen drier yielding 225.70 mg (SGG-a), 255.20 mg (SGG-b), 247.20 mg (SGG-c), 250.00 mg (M400-a), 176.00 mg (M400-b), 207.80 mg (M400-c), 264.80 mg (R358-a), 297.50 mg (R358-b), 310.50 mg (R358-c), 89.05 mg (SGG-blank), 85.05 mg (M400-blank), and 71.12 mg (R358-blank).

### 3.4. Disc Diffusion Assay of Crude Extracts

The antibacterial activity of extracts M400, R358, and SGG was tested using a disc diffusion assay following a previously described method [40,55]. Therefore, 8 mg/mL concentration solutions of the extracts and extracts’ media controls were prepared, loaded on sterile filter paper (6 mm disc), and allowed to dry. The inoculum pathogen broth (2 mL) prepared according to the 0.5 McFarland standard was spread on Mueller Hinton agar plates. Sterile dried filter paper discs loaded with methanol were used as the negative control and a 10 µg concentration of gentamicin discs (ThermoFisher Scientific Ltd, Cheshire, UK) as the positive control. The extract filter paper, media blanks, solvent control, and antibiotic discs were impregnated into the agar plates and incubated at 28 °C for 24 h and the diameter of inhibition zones was measured. Antibacterial activity was evaluated by measuring the zone of inhibition around the disc using a transparent millimetre scale. The bioassay experiment was repeated twice.

### 3.5. Large-Scale Fermentation of Streptomyces sp. B188M101

The large-scale fermentation (8 L) was prepared by the following process: 5 mL of a 200 mL seed culture of *Streptomyces* sp. B188M101 was transferred with a disposable sterile serological pipet into 40 × 1 L baffled Erlenmeyer flasks containing autoclaved 200 mL R358 media solution. After inoculation, they were incubated for 10 days at 28 °C and shaken at 180 rpm in a closed system shaker. When fermentation was completed, 12 g of autoclaved Diaion HP-20 resin was added to each flask and allowed to shake for 24 h before removing the resin and subsequently extracted with methanol (800 mL × 3) followed by ethyl acetate (300 mL × 2). The combined organic extract was dried and yielded 16.45 g of crude extract.

### 3.6. Fractionation and Isolation of Metabolites from Streptomyces sp. B188M101

The R358 crude extract (16.45 g) was subjected to fractionation by Kupchan partitioning [55]. The extract was partitioned between aqueous (H_2_O, 700 mL) and organic phases (dichloromethane, 700 mL) in a 2 L round bottom flask. The aqueous phase (16.15 g) was further extracted with *sec*-butanol (300 mL × 3) yielding FB (3.85 g) and FW (8.80 g). The dichloromethane fraction (210 mg) was discarded upon chemical analysis. The FB fraction was chromatographed using a reveries BUCHI preparative flash system (Flawil, Switzerland) equipped with a reversed-phase EcoFlex C18 cartridge (80 g, 50 µm), stationary phase cartridge, and variable UV and ELSD detectors to monitor the run at 210, 230, and 254 nm. Chromatographic elution started with a mobile isocratic scheme of 100% H_2_O and run for 7 min followed by linear gradient elution to 100% MeOH for 80 min and finally maintained at 100% MeOH for 20 min at the flow rate of 7 mL /min with a combined running time of 94 min to afford FB1 (225.08 mg, t_R_ 2–13 min), FB2 (111.28 mg, t_R_ 15–19 min), FB3(97.55 mg, t_R_ 20–26 min), FB4 (76.17 mg, t_R_ 29–39 min), FB5 (32.38 mg, t_R_ 45–48 min), FB6 (20.29 mg, t_R_ 49–53 min), FB7 (31.62 mg, t_R_ 56–62 min), FB8 (35.66 mg, t_R_ 63–66 min), FB9(20.13 mg, t_R_ 67–70 min), FB10 (17.59 mg, t_R_ 71–75 min), FB11 (27.52 mg, t_R_ 76–80 min), and FB12 (61.52 mg, t_R_ 83–89 min). Following the chemical dereplication of the FB fractions, the FB6-FB9 fractions (107.7 mg) were combined and purified using a reversed-phase HPLC, ACE (5 µm 10 × 250 mm) with a gradient elution of 95: 5% H_2_O: MeCN for 15 min to 38% MeCN, followed by a gradient elution for 30 min to 50% H_2_O: MeCN and continued for another 20 min to 80% MeCN at the flow rate of 2 mL/min for a combined run of 50 min yielding co-eluting gausemycin derivatives (6.12 mg, t_R_ 20–23 min) and compounds **1** (3.5 mg, t_R_ 26.2 min), **2** (0.8 mg, t_R_ 34.8 min), **3** (5 mg, t_R_ 30.4 min), and **4** (5 mg, t_R_ 27.1 min). The gausemycin mixture was further purified by re-injection with a starting gradient elution of 90:5% H_2_O: MeCN to 20% MeCN at 40 min, 38% MeCN at 70 min up to 50% MeCN for 80 min at the flow rate of 2 mL/min, yielding gausemycin A (**5**) (2.7 mg, t_R_ 55 min).

Ageloline B (**1**): yellow powder; [α]_D_^25^ −17.7 (c 0.225, MeOH); UV (MeOH) λ_max_, nm (log ε): 194 (2.94); 234(2.91), 266(2.64), 358(3.41); HRESIMS (*m*/*z*): [M+H]^+^ calcd. for C_12_H_14_ClN_2_O_4_, 285.0638; found 285.0607(Δ: +0.5 ppm); NMR data, see Table 1 and Table S2 and Figures S11–S17 and S43.

Ageloline C (**2**): light yellowish powder; HRESIMS (*m*/*z*): [α]_D_^25^ −40 (c = 0.01 g/mL, MeOH); UV (MeCN-H_2_O)) λ_max_, nm (log ε): 194 (3.46); 234 (3.63), 254(3.24), 362(3.03); [M+H]^+^ calcd. for C_13_H_16_ClO_4_N_2_, 299.0795; found, 299.0793 (Δ: +0.5 ppm); NMR data, see Table 1 and Table S3 and Figures S18–S22 and S44.

Ageloline D (**3**): yellow powder; HRESIMS (*m*/*z*); UV (MeCN-H_2_O λ_max_, nm (log ε): 194 (2.86); 222 (2.33), 254(2.89), 346(2.66); [M+H]^+^ calcd. for C_7_H_7_ClO_4_N_2_, 172.0163; found, 172.0160 (Δ: +1.3 ppm); NMR data, see Table 1 and Table S4 and Figures S24–S28 and S45.

### 3.7. Antimicrobial Activity

The antibacterial activity of compounds **1**–**5** was determined against strains from the NAICONS collection of bacterial pathogens, including ATCC strains, as follows: 90 μL of 1–6 × 10^5^ CFU/mL bacterial suspension was dispensed into each well of a 96-well plate containing 10 μL of serial 1:2 dilutions of compound **1**–**5** (from a 10 mg/mL stock solution in 100% DMSO). All the strains were grown in Müller Hinton broth except for *Streptococcus* spp. and *Candida albicans* strains, which were grown in Todd Hewitt broth and Yeast Peptone Dextrose medium, respectively. Sterile solutions of CaCl_2_ in water were added to the media at the time of the experiments to obtain the final concentrations of 450 µM or 20 mM. Plates were incubated at 37 °C in a Synergy2 microplate reader (BioTek Instruments, Winooski, VT, USA) with readings at 595 nm registered every hour.

### 3.8. MZmine 4.20 Data Pre-Processing, MetaboAnalyst 5.0, and Feature-Based Molecular Networking

The biological replicate MS data was converted into mzML format using MSConvert from the ProteoWizard [42] and imported to MZmine (version 4.2.0), a scalable MS data analysis platform that supports hybrid datasets from various instrumental setups [43]. The mzML data was pre-processed with mass ion detection achieved through the removal of MS1 and MS2 baseline noise levels set to 1.0 × 10^4^ and 1.0 × 10^2^, respectively. The chromatogram construction was performed for each of the centroided MS1 detected using the ADAP chromatogram builder, where minimum consecutive scans, minimum intensity for consecutive scans, minimum absolute height, and *m*/*z* mass tolerance were set to 4, 1.0 × 10^2^, 1.0 × 10^4^, and 5 ppm, respectively. The peak detection utilised local minimum resolver as a deconvolution algorithm to construct the chromatogram of each mass ion while keeping the original feature list created in the previous step. For this algorithm, the chromatographic threshold was set to 85%, the minimum search range in the RT absolute range to 0.05 min, the minimum relative height to 0.0%, the minimum absolute height to 2.0 × 10^6^, and the minimum ratio of peak top/edge to 1.70. The identified peaks were deisotoped using the isotopic peaks grouper, in which the *m*/*z* tolerance (intra-sample) was set to 5 ppm, the RT tolerance to 0.02 for absolute minutes, maximum charge of 3 absolute minutes, and the representative isotope as the most intense. The deisotoped peaks were then aligned using a join aligner to correct any deviation of the retention time of each mass peak: the ion *m*/*z* tolerance for the alignment was set to 10 ppm, retention time tolerance to 0.06 at absolute minute, weight for *m*/*z* and RT set to 75 and 25, respectively, and the mobility weight was set to 1. The peak list produced after the alignment step was filtered using a feature list row filter algorithm to remove any mass ions outside the set range of 100 to 2000 *m*/*z* and keep peak rows that match all criteria.

The resulting feature list, including the retention time, mass to charge ratio, and peak area rows, was exported by converting into a MetaboAnalyst format CSV file, a text format that presents a set of data in Excel style. For this study, CSV files were generated in triplicate for each biological replicate sample and converted to a zip folder before uploading to the MetaboAnalyst 5.0 platform with the option of One Factor-Statistical Analysis [75]. The processed MS peak list data was pre-processed by setting the mass tolerance and retention time tolerance to 0.025 and 30 s, respectively. All features with missing values generated due to poor repeatability and below the limit of detection in the triplicates were replaced with 1/5 of the minimum positive values below their corresponding values by setting the threshold to 20%. The features were further filtered using the interquartile range (IQR), with the relative standard deviations (RSDs) set at 25%. Normalization by sum was performed for the feature peak list, followed by log transformation (base 10) and pareto scaling to transform the data to more a Gaussian-type normal distribution that allows for easy comparability in a graphical style. The above-mentioned parameters were used to generate the Principal Component Analysis (PCA), Dendrogram, and Heatmaps used in this study.

The respective MS feature lists of MS1 and MS2 were exported as quant.csv and MGF files—the formats used to create Feature-Based Molecular Networking (FBMN) analysis on the Global Natural Social Networking (GNPS) [41,76]. For generating the networking, both the precursor ion and MS/MS fragment ion tolerance were set to 0.05 Da. A molecular network was then created with edges filtered to have a cosine score above 0.50 and at least six matched peaks. The top 20 most similar nodes were kept. The spectra in the network were then searched against GNPS spectral libraries [76]. The DEREPLICATOR was used to annotate MS/MS spectra [77] and the molecular networks generated were visualized using Cytoscape software version 3.10.0 [37].

## 4. Conclusions

In this study, we isolated and structurally elucidated three new chlorinated natural products, agelolines B–D (**1**–**3**), alongside two previously reported metabolites, ageloline A (**4**) and gausemycin A (**5**), using metabolomic analysis combined with bioassay-guided purification. The antimicrobial activity of compounds **1**–**5** was assessed against the fish pathogen *A. salmonicida*, with gausemycin A (**5**) exhibiting pronounced activity at the minimum inhibitory concentration (MIC). Subsequent screening against a panel of human pathogens revealed that compound **3** displayed notable inhibitory effects against *S. aureus* ATCC6538P and *S. maltophilia* L2125. Interestingly, the addition of calcium salts enhanced the activity of compound **5** against *A. salmonicida*, resulting in a two-fold increase in potency.

Elucidating the mechanistic basis underlying the selective inhibition of *A. salmonicida* by compound **5** remains a central focus of our future investigations. To date, lipoglycopeptide antibiotics have not been reported to exhibit such activity against Gram-negative bacteria, highlighting the novelty of our findings. To support further biological and structural studies, efforts are currently underway to upscale the isolation and characterization of the newly identified gausemycin congeners dereplicated in this study.

## Figures and Tables

**Table 1:** ^1^H and ^13^C NMR data of compounds **1**–**3** (CD_3_OD, 600 MHz).

	1		2		3	
Position	δ_C_, Type	δ_H_, Mult. (*J* in Hz)	δ_C_, Type	δ_H_, Mult. (*J* in Hz)	δ_C_, Type	δ_H_, Mult. (*J* in Hz)
1	116.7, C		116.7, C		110.3, C	
2	153.3, C		153.3, C		153.6, C	
3	116.9, CH	6.77 (d, 2.2)	116.9, CH	6.77 (d, 2.2)	116.3, CH	6.74 (d, 2.2)
4	141.0, C		141.0, C		140.5, C	
5	115.9, CH	6.56 (dd, 8.7, 2.2)	115.9, CH	6.56 (dd, 8.7, 2.2)	116.0, CH	6.50 (dd, 8.7, 2.2)
6	133.6, CH	7.72 (d, 8.7)	133.6, CH	7.71 (d, 8.7)	133.9, CH	7.77 (d, 8.7)
7	198.1, C		198.1, C		170.8, C	
8	41.2, CH_2_	3.54 (dd, 17.3, 6.3), 3.45 (dd, 16.9, 6.3)	41.2, CH_2_	3.53 (dd, 17.3, 6.3), 3.46 (dd, 16.9, 6.3)		
9	49.3, CH	4.89 (m)	49.3, CH	4.91 (m)		
10	173.1, C		173.1, C			
11	172.9, C		172.9, C			
12	21.8, CH_3_	1.97 (s)	21.8, CH_3_	1.96 (s)		
13			51.5, CH_3_	3.72 (s)		

**Table 2:** In-vitro antimicrobial activity of compounds **1**–**5**.

Compound MIC (µg/mL)					
Pathogenic Species	1	2	3	4	5
*Staphylococcus aureus* ATCC6538P	128	>128	6	128	32
*S. aureus* L3797	>128	>128	>128	>128	>64
*S. pneumoniae* L44	>128	>128	64	128	128
*S. maltophilia* L2125	128	64	32	64	nt
*Aeromonas salmonicida*	>128	>128	>128	>128	32
*Pseudomonas aeruginosa*	>128	>128	>128	>128	>128
*E. coli* ΔtolC L4242	>128	>128	>128	>128	>128

**Table 3:** In-vitro antimicrobial activity of compound **5** after supplemented with CaCl_2_.

Pathogenic Species	0 mM CaCl_2_	0.45 mM CaCl_2_	20 mM CaCl_2_
*Staphylococcus aureus* ATCC6538P	32	4	≤4
*S. aureus* GISA L3797	>64	16	32
*S. pneumoniae* L44	128	128	nt
*Aeromonas salmonicida*	32	16	16
*Pseudomonas aeruginosa*	>128	>128	>128
*E. coli* ΔtolC L4242	>128	>128	>64

## Data Availability

Data available on request from the corresponding author.

## Supplementary Materials

The following supporting information can be downloaded at https://www.mdpi.com/article/10.3390/md23090362/s1. Figure S1: MS/MS fragmentation of gausemycin A at the cyclic core (922.3815 *m*/*z*) and tail substructure fragmentation at the C-terminal end. [A]—represented the structure of gausemycin A, indicating the fragmentation pathway and explanation. Figure S2: The tail fragmentation of gausemycin A at the N-terminal and the neutral loss of the arabinose moiety. Figure S3: The tail fragmentation of gausemycin B at the C-terminal showing the additional amino acid (β-Alanine) attachment at Orn-2. Figure S4: The tail fragmentation of gausemycin C from Ahpb-3 to the fatty acid chain showing the loss of methyl-β-Alanine from the attachment on Orn-2. Figure S5: (+)-HRESIMS spectrum of putative new molecules at *m*/*z* 864.8879 (M+2H)^+2^. Figure S6: The tail fragmentation of putatively new molecules at *m*/*z* 864.8879 (M+2H)^+2^ group attachment to ornithine of the gausemycin D analogue. Figure S7: (+)-HRESIMS spectrum of putative new molecules at *m*/*z* 930.9088 (M+2H)^+2^. Figure S8: The tail fragmentation of putatively new molecules at *m*/*z* 930.9088 (M+2H)^+2^ indicating attachment to ornithine of the gausemycin A analogue. Figure S9: (+)-HRESIMS spectrum of putative new molecules at *m*/*z* 1945.8621 corresponding to 973.4351 (M+2H)^+2^. Figure S10: The tail fragmentation of putatively new molecules at *m*/*z* at *m*/*z* 1945.8621 corresponding to 973.4351 (M+2H)^+2^ showing dimethyl-β-alanine attachment to ornithine of the gausemycin A analogue. Figures S11 and S12: (+)-HRESIMS and ^1^H NMR spectra of **1**. Figures S13–S14: HSQC and COSY NMR spectra of **1**. Figures S15 and S16: HMBC and COSY (DMSO-d6) NMR spectra of **1**. Figures S17 and S18: NOESY spectrum of **1** and (+) HRESIMS of **2**. Figures S19 and S20: ^1^H NMR and HSQC spectra of **2**. Figures S21 and S22: COSY and HMBC spectra of **2**. Figures S23–S24: Extracted ion chromatogram of **1** and **2**, and (+)-HRESIMS spectra of **3**. Figures S25 and S26: ^1^H NMR of **3** and HSQC spectrum of **3**. Figures S27 and S28: COSY and HMBC spectra of **3**. Figures S29 and S30. (+)-HRESIMS and ^1^H NMR spectra of **4**. Figures S31 and S32: HSQC and COSY NMR spectra of **4**. Figures S33 and S34: HMBC spectrum of **4** and (+)-HRESIMS spectrum of **5**. Figures S35 and S36: ^1^H NMR and HSQC spectra of **5**. Figure S37: COSY spectrum of **5**. Figure S38: Phylogenetic characterisation of *Streptomyces* sp. B188M101 by the Genome Taxonomy Database (GTDB-Tk). Figure S39: (A) Comparison of gausemycin-producing BGC from *Streptomyces* sp. B188M101 with most closely related known clusters from the MIBiG database. (B) Alignment of the gausemycin-producing BGC from *Streptomyces* sp. B188M101 (top) and the gausemycin cluster from *Streptomyces* sp. INA-Ac-5812 (bottom), and functional annotation of the B188M101 cluster. Note: the cluster from *Streptomyces* sp. INA-Ac-5812 has been reversed in orientation for improved visualisation of homology. Figure S40: Growth curve inhibition of **3** against *S. S.aureus* ATCC6538P (A) and *S. maltophilia* L21259 (B); Figure S41: Growth curve inhibition of **2** and **4** against *S. maltophilia* L21259 (A) and S. aureus ATCC6538P (B), respectively. Figure S42: (A) Growth curve inhibition of **5** against *A. salmonicida* without CaCl_2_ and (B) represents with CaCl_2_ supplement. Figures S43 and S44: UV absorption of **1** and **2**. Figure S45: UV absorption of **3**. Table S1: Putative chemical dereplication of detected metabolites in the *Streptomyces* B188M101_R358 extract. Table S2: ^1^H and ^13^C NMR data of **1** (CD_3_OD, 600/150 MHz). Table S3: ^1^H and ^13^C NMR data of **2** (CD_3_OD, 600/150 MHz) Table S4: ^1^H and ^13^C NMR data of **3** (CD_3_OD, 600/150 MHz). Table S5: ^1^H and ^13^C NMR data of **4** (CD_3_OD, 600/150 MHz). Table S6: ^1^H and ^13^C NMR data of **5** (DMSO-d6, 600/150 MHz). Table S7: MS2 fragmentation sequences of compound **5** using Orbitrap HR-ESI-MS. Table S8: Marfey’s derivatisation analysis of compound **5** hydrolysates. Table S9: Annotation of open reading frames (ORFs) in the NRPS/betalactone biosynthetic gene cluster from the genome of *Streptomyces* sp. B188M101. Table S10: Homologous ORFs in the gausemycin BGC of *Streptomyces* sp. INA-Ac-5812 and that of *Streptomyces* sp. B188M101. Reference [78] is cited in the supplementary materials.
