# Supplementary material for: Integrated Omics-Based Discovery of Bioactive Halogenated Metabolites from the Deep-Sea Streptomyces sp. B188M101
Source: Mar Drugs. 2025 Sep 19;23(9):362. doi: 10.3390/md23090362 (PMC12471526; doi:10.3390/md23090362)
Supplement: Supplementary file 1 [file marinedrugs-23-00362-s001.zip › marinedrugs-3850075-SI.pdf]

Integrated Omics-Based Discovery of Bioactive Halogenated Metabolites from the Deep-Sea *Streptomyces* sp. B188M101

Emmanuel Tope Oluwabusola[1,†,*], Stephen A. Jackson[2,3,†], Cristina Brunati [4], Stefanie Gackstatter[5], Hannah Vedder[6], Marianna Iorio[4], Gargee Chawande[2], Lekha Menon Margassery [2], Giang-Son Nguyen[7], David J. Clarke[2], Rainer Ebel[1], Marcel Jaspars[1], Alan D.W. Dobson[2,3]

[1] Marine Biodiscovery Centre, Department of Chemistry, University of Aberdeen, Scotland, UK.
[2] School of Microbiology, University College Cork, College Road, Cork, Ireland.
[3] [3]Sustainability Institute, University College Cork, Lee Road, Cork, Ireland.
[4] NAICONS, viale Ortles 22/4, 20139, Milan, Italy.
[5] Interfaculty Institute of Biochemistry, University of Tübingen, Tübingen, Germany.
[6] Department of Pharmacy, Philipps University Marburg, Marburg, Germany.
[7] Department of Biotechnology and Nanomedicine, SINTEF Industry, Trondheim, Norway.

* 'these authors contributed equally'
* Author to whom correspondence should be addressed.

Additional analytical and experimental information

Table of contents

Figure S1: MS/MS fragmentation of gausemycin **A** at the cyclic core (922.3815 m/z) and tail substructure fragmentation at the C-terminal end. [**A]** — represented the structure of gausemycin **A**, indicating the fragmentation pathway and explanation
Figure S2: The tail fragmentation of gausemycin **A** at the N-terminal and the neutral loss of the arabinose moiety.
Figure S3: The tail fragmentation of gausemycin **B** at the C-terminal showing the additional amino acid (β-Alanine) attachment at Orn-2**.**
Figure S4: The tail fragmentation of gausemycin **C** from Ahpb-3 to the fatty acid chain showing the loss of methyl-β-Alanine from the attachment on Orn-2.
Figure S5. (+)-HRESIMS spectrum of putative new molecules at m/z 864.8879 $(M+2H)^{+2}$
Figure S6. The tail fragmentation of putatively new molecules at m/z 864.8879 $(M+2H)^{+2}$ group attachment to ornithine of the gausemycin D analogue.
Figure S7. (+)-HRESIMS spectrum of putative new molecules at m/z 930.9088 $(M+2H)^{+2}$
FigureS8. The tail fragmentation of putatively new molecules at m/z 930.9088 $(M+2H)^{+2}$ indicating attachment to ornithine of the gausemycin A analogue.
Figure S9. (+)-HRESIMS spectrum of putative new molecules at m/z 1945.8621 corresponding to 973.4351 $(M+2H)^{+2}$
Figure S10. The tail fragmentation of putatively new molecules at m/z at m/z 1945.8621 corresponding to 973.4351 $(M+2H)^{+2}$ showing dimethyl-β-alanine attachment to ornithine of the gausemycin A analogue
Figure S11-S12: (+)-HRESIMS and ^{1}H NMR spectra of **1.**
Figure S13-S14: HSQC and COSY NMR spectra of **1.**
Figure S15-S16: HMBC and COSY *(DMSO-d6)* NMR spectra of **1**.
Figure S17-S18: NOESY spectrum of **1** and (+) HRESIMS of **2**.
Figure S19-S20: ^{1}H NMR and HSQC spectra of **2**.
Figure S21-S22: COSY and HMBC spectra of **2**.
Figure S23- S24: Extracted ion chromatogram of **1** and **2,** and (+)-HRESIMS spectra of **3**.
Figure S25-S26: ^{1}H NMR of **3** and HSQC spectrum of **3**.
Figure S27-S28: COSY and HMBC spectra of **3.**
Figure S29-S30. (+)-HRESIMS and ^{1}H NMR spectra of **4**.
Figure S31-S32: HSQC and COSY NMR spectra of **4**.
Figure S33-S34: HMBC spectrum of **4** and (+)-HRESIMS spectrum of **5.**
Figure S35-S36: ^{1}H NMR and HSQC spectra of **5**.
Figure S37: COSY spectrum of **5**.
Figure S38: Phylogenetic characterisation of *Streptomyces sp.* B188M101 by the Genome Taxonomy Database (GTDB-Tk).
Figure S39: (**A**) Comparison of gausemycin-producing BGC from *Streptomyces* sp. B188M101 with most closely related known clusters from the MIBiG database. (**B**) Alignment of the gausemycin-producing BGC from *Streptomyces* sp. B188M101 (top) and the gausemycin cluster from *Streptomyces* sp. INA-Ac-5812 (bottom), and functional annotation of the B188M101 cluster. Note: the cluster from *Streptomyces* sp. INA-Ac-5812 has been reversed in orientation for improved visualisation of homology.
Figure S40: Growth curve inhibition of **3** against S. S.aureus ATCC6538P (A) and S. maltophilia L21259 (B);
Figure S41: Growth curve inhibition of **2** and **4** against *S. maltophilia* L21259 (A) and S. aureus ATCC6538P (B), respectively.
Figure S42: (**A**) Growth curve inhibition of **5** against *A. salmonicida* without $CaCl_2$ and (**B**) represents with $CaCl_2$ supplement.
Figure S43-S44: UV absorption of **1 and 2**.
Figure S45: UV absorption of **3**.
Table S1: Putative chemical dereplication of detected metabolites in the *Streptomyces* B188M101_R358 extract.
Table S2: ^{1}H and ^{13}C NMR data of **1** (CD_3OD, 600/150 MHz).
Table S3: ^{1}H and ^{13}C NMR data of **2** (CD_3OD, 600/150 MHz)
Table S4: ^{1}H and ^{13}C NMR data of **3** (CD_3OD, 600/150 MHz).
Table S5: ^{1}H and ^{13}C NMR data of **4** (CD_3OD, 600/150 MHz).
Table S6: ^{1}H and ^{13}C NMR data of **5** (DMSO-d6, 600/150 MHz).
Table S7: MS2 fragmentation sequences of compound **5** using Orbitrap HR-ESI-MS.
Table S8: Marfey's derivatisation analysis of compound **5** hydrolysates.
Table S9: Annotation of open reading frames (ORFs) in the NRPS/betalactone biosynthetic gene cluster from the genome of *Streptomyces* sp. B188M101.
Table S10: Homologous ORFs in the gausemycin BGC of *Streptomyces* sp. INA-Ac-5812 and that of *Streptomyces* sp. B188M101.

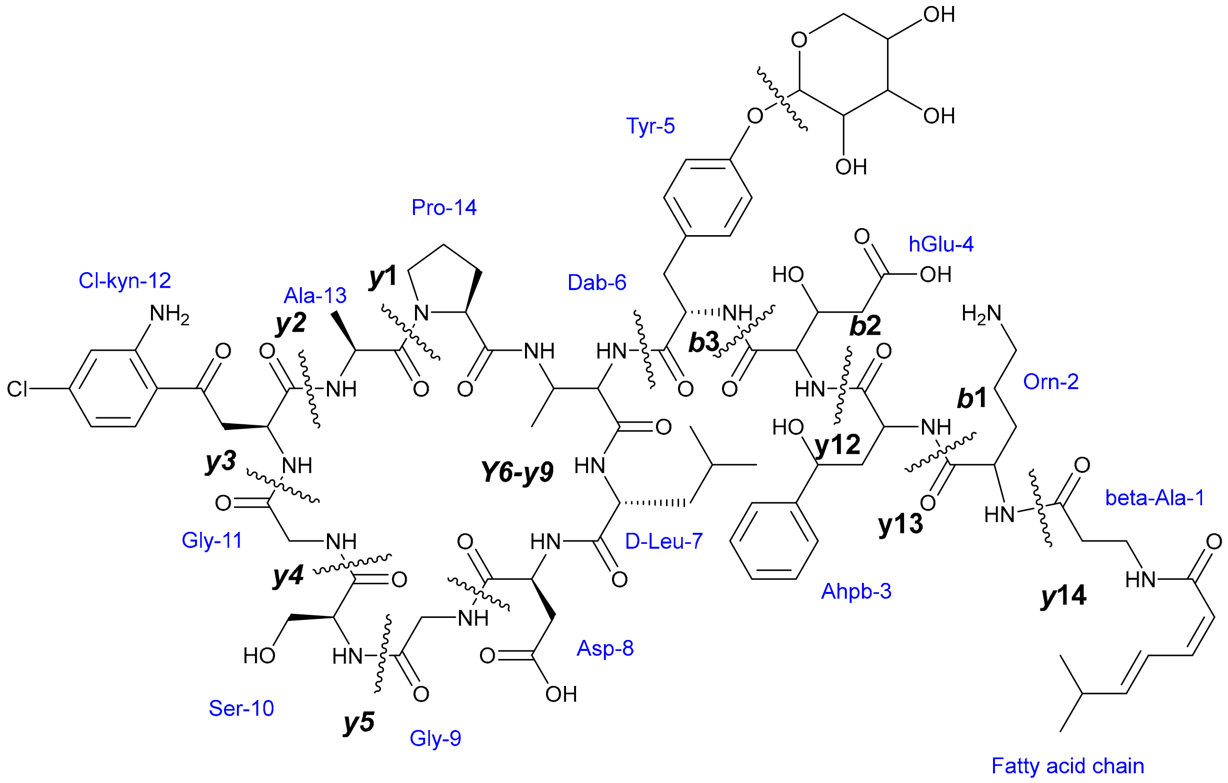

(A)

In the lower mass region, a sequential tail of fragment ions was annotated, corresponding to a fatty acid chain attached to β-alanine (y_{14}), ornithine (y_{13}), and Ahpb (y_{12}), with observed m/z values of 485.2757, 308.1969, and 194.1175, respectively. Subsequently, higher *m/z* fragment ions revealed an N-terminal fragmentation pathway characterized by neutral losses of 132, 163, and 145 Da. These losses are consistent with the presence of arabinose (Ara) glycosidically linked to the phenolic hydroxyl group of a tyrosine residue, along with adjacent amino acid sequences (Ara–Tyr–hGlu–Ahpb), corresponding to the b_1, b_2, and b_3(+Ara) ions. Notably, the tyrosine residue was identified as a direct linker between the cyclic core and the tail, resulting in a prominent peak at *m/z* 922.3815, which represents the fragment ion corresponding to the structural cyclic core (Table S7 and Figure S1-S2).

For the core fragment ion of gausemycin A, the fragment sequence loss of amino acid residues follows Ala-Cl-kyn-Gly-ser-Gly corresponding to y1–y2–y3–y4–y5 ion were observed. The remaining amino acid residuals—Pro-Asp-Leu—`along with a non-proteinogenic residue, 2,4-diaminobutyric acid (Dab, 100 Da) were identified based on the observed fragment ions y6–y7–y8–y9 as shown in in the fragmentation spectrum (Figure S1)

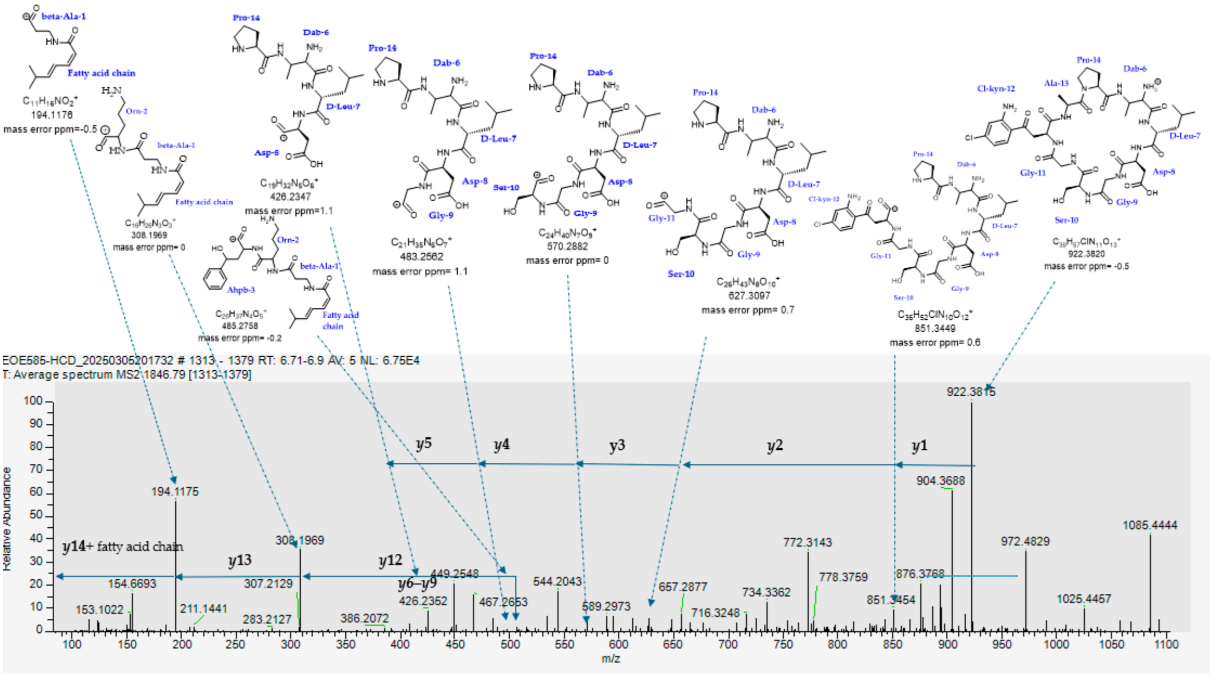

Figure S1. MS/MS fragmentation of gausemycin **A** at the cyclic core (922.3815 m/z), tail substructure fragmentation at the C-terminal end. **A —** represented the structure of gausemycin **A** indicating fragmentation pathway.

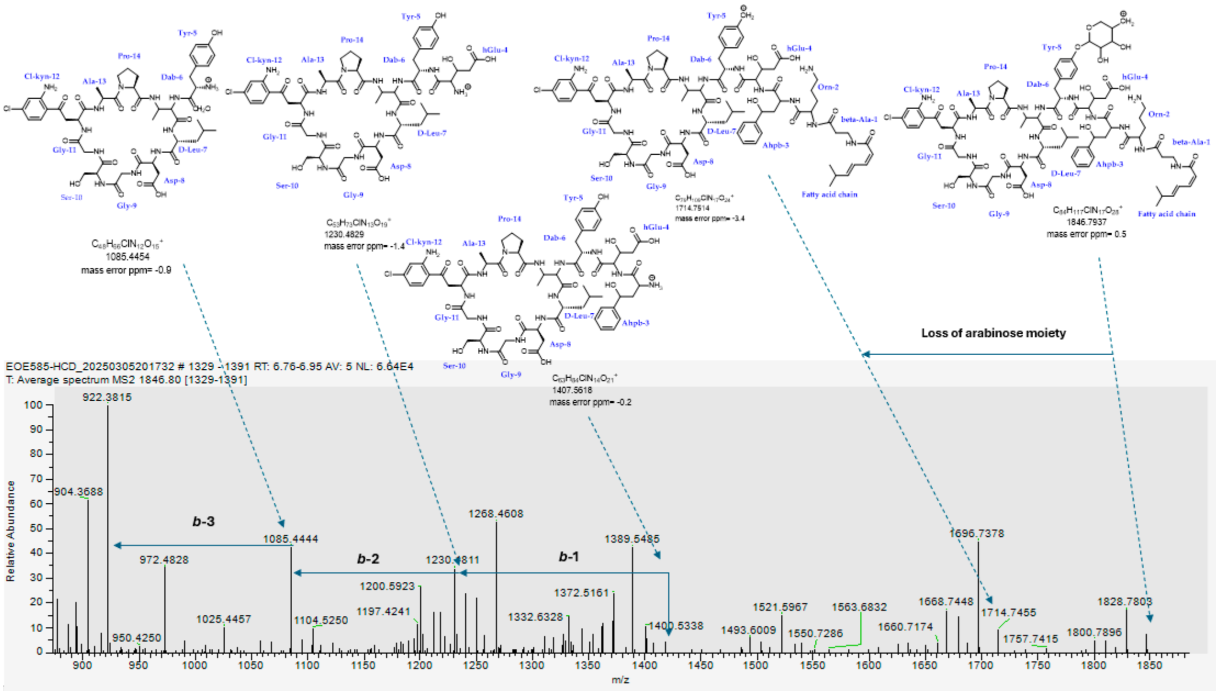

Figure S2. The tail fragmentation of gausemycin **A** at N-terminal and neutral loss of arabinose moiety.

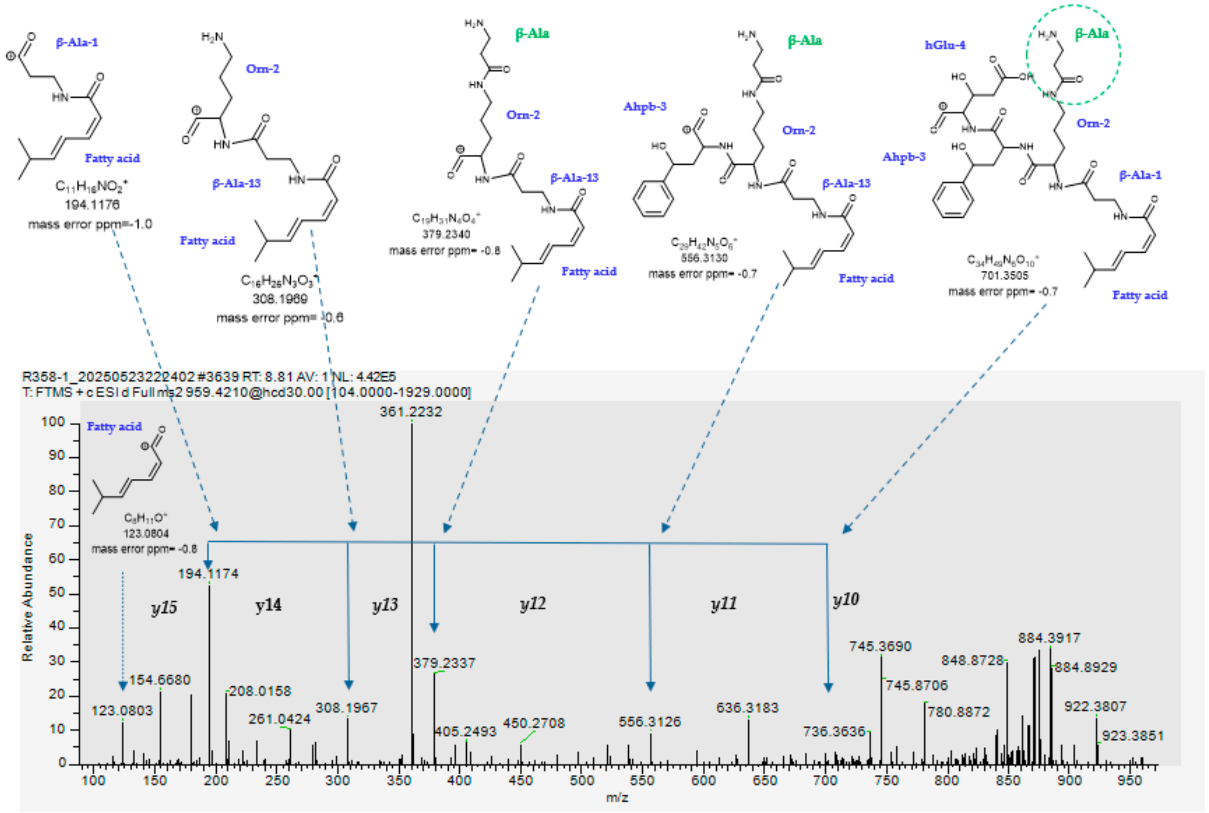

Figure S3. The tail fragmentation of gausemycin **B** at C-terminal showing the additional amino acid (β-Alanine) attachment at **Orn-2.**

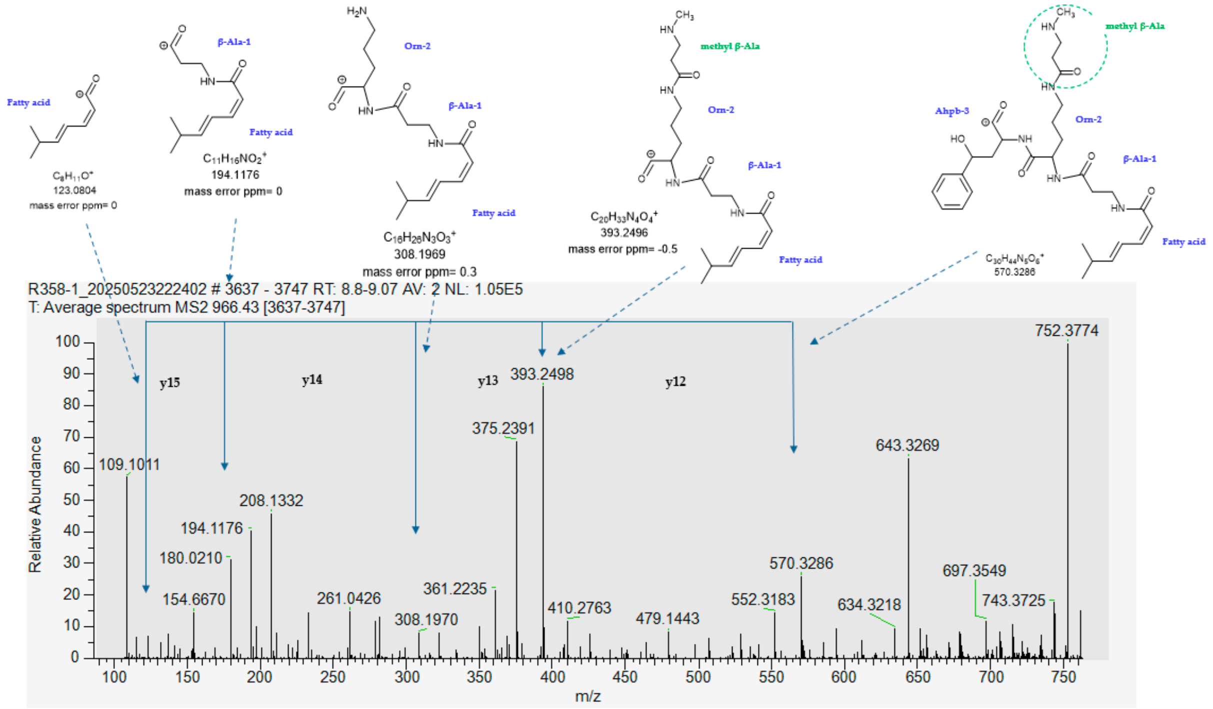

Figure S4. The tail fragmentation of gausemycin **C** from Ahpb-3 to fatty acid chain showing loss of methyl-β-Alanine from attachment on Orn-2.

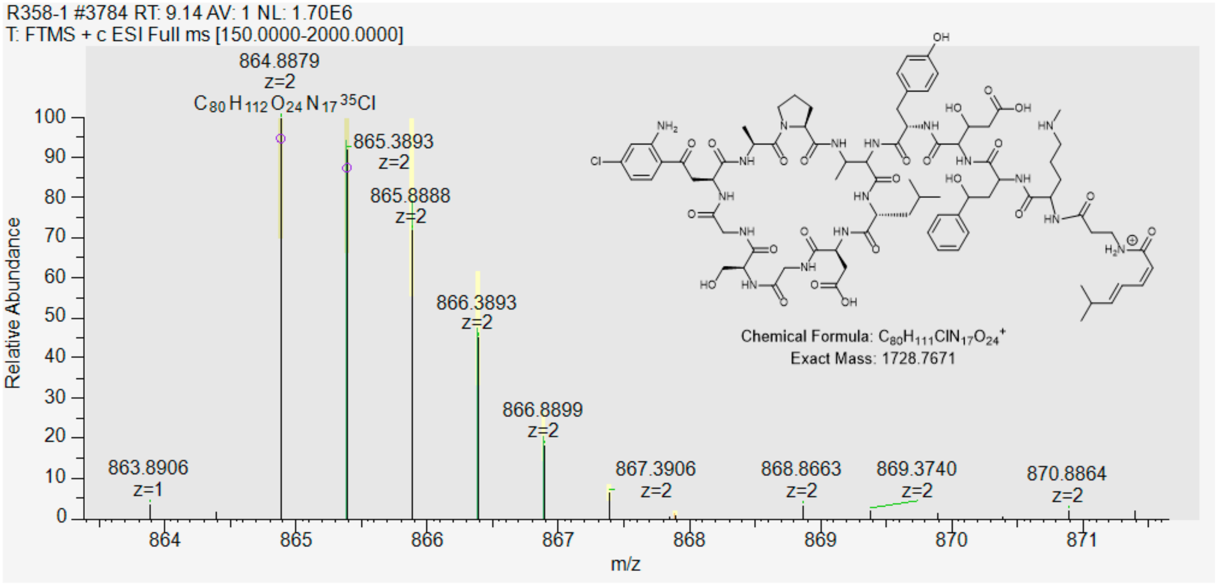

Figure S5. (+)-HRESIMS spectrum of putative new molecules at m/z **864.8879** **(**M+2H)[+2]

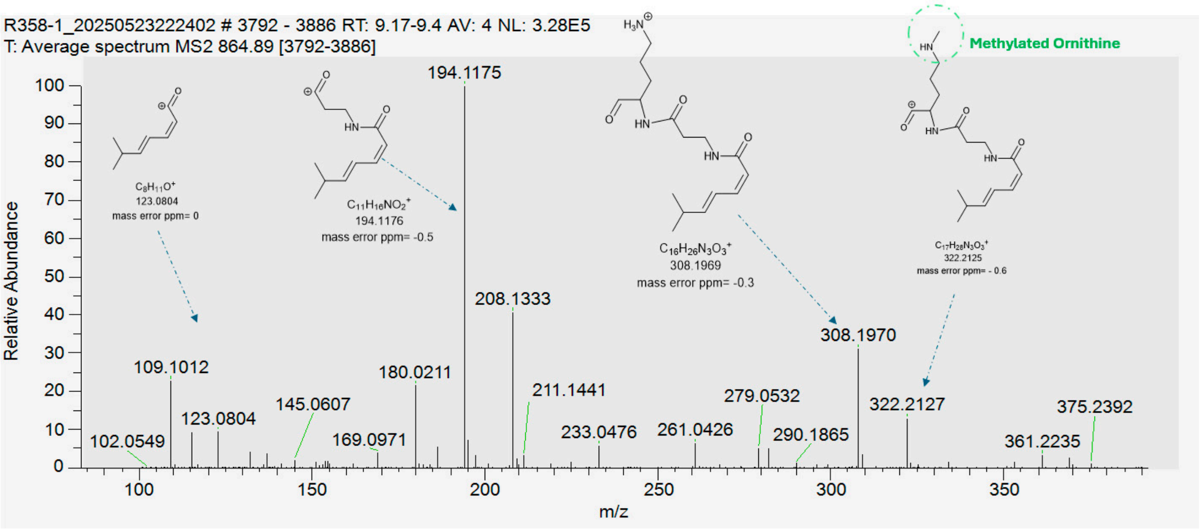

Figure S6. The tail fragmentation of putatively new molecules at m/z 864.8879 (M+2H)$^{+2}$ group attachment to ornithine of the gausemycin D analogue.

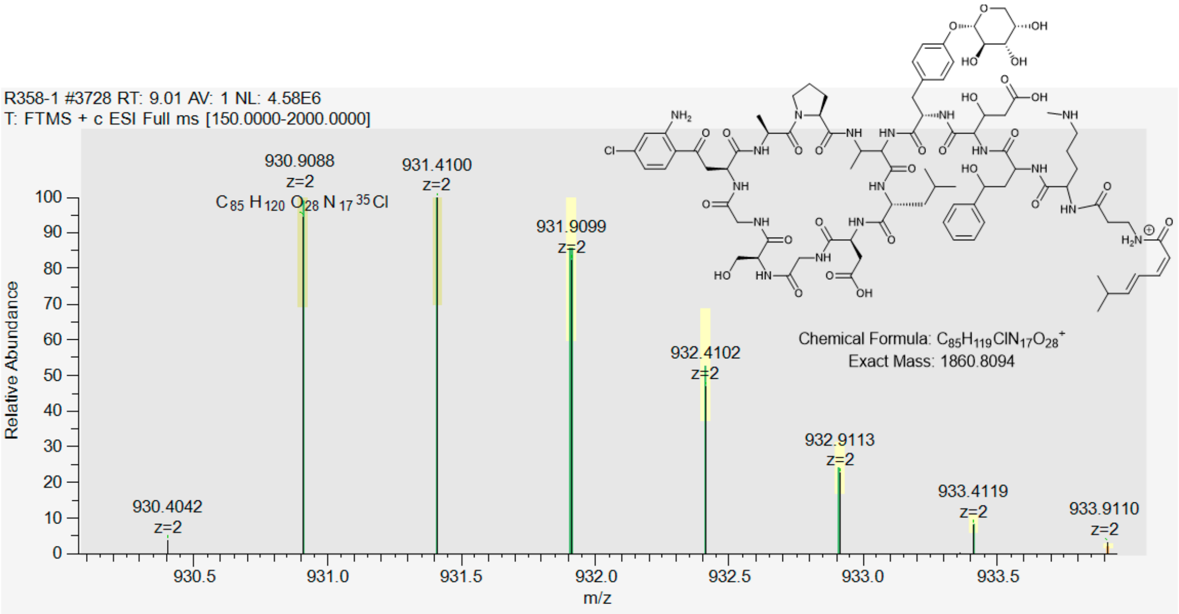

Figure S7. (+)-HRESIMS spectrum of putative new molecules at m/z **930.9088 (**M+2H)$^{+2}$

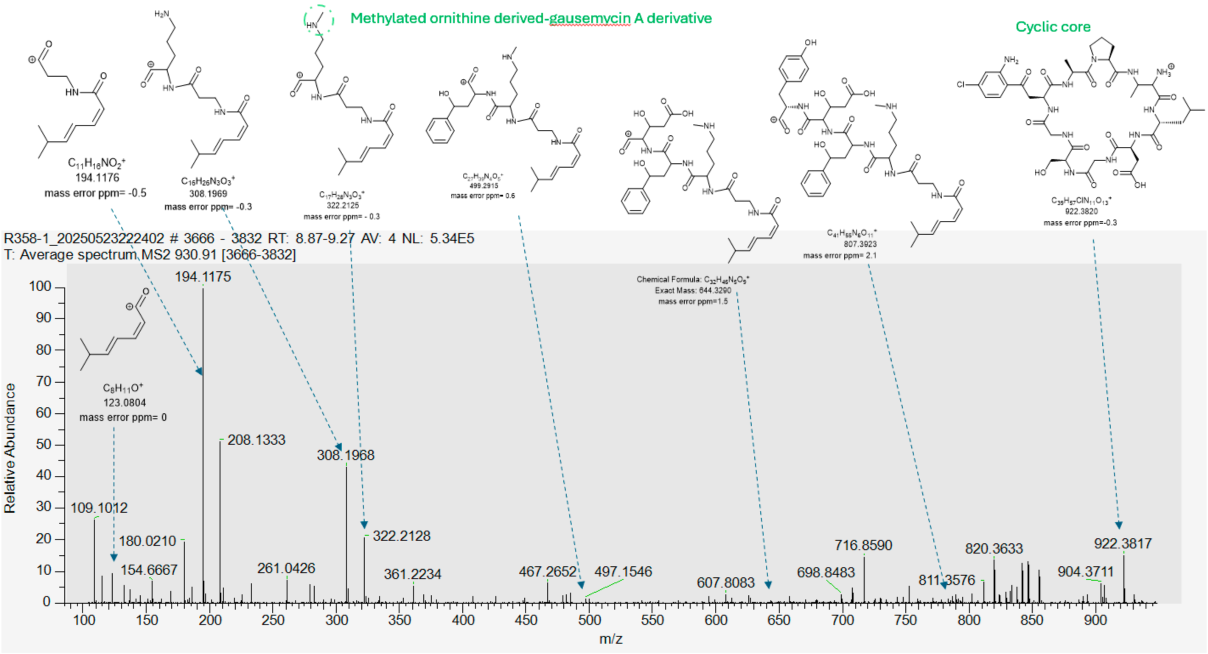

Figure S8. The tail fragmentation of putatively new molecules at m/z **930.9088 (**M+2H)$^{+2}$ indicating attachment to ornithine of the gausemycin A analogue.

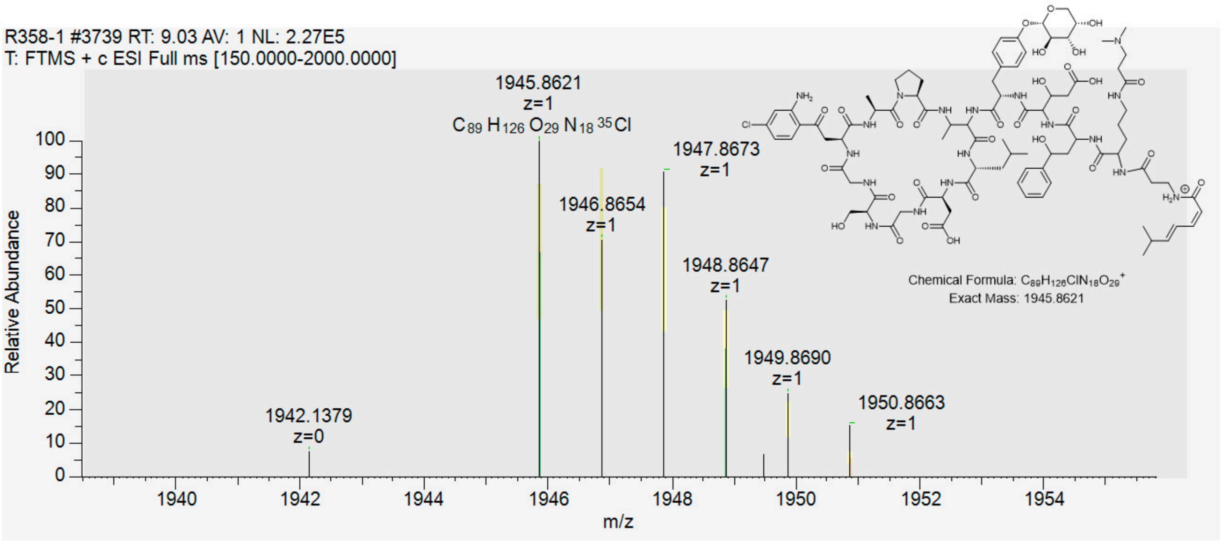

Figure S9. (+)-HRESIMS spectrum of putative new molecules at m/z 1945.8621 corresponding to 973.4351 (M+2H)$^{+2}$

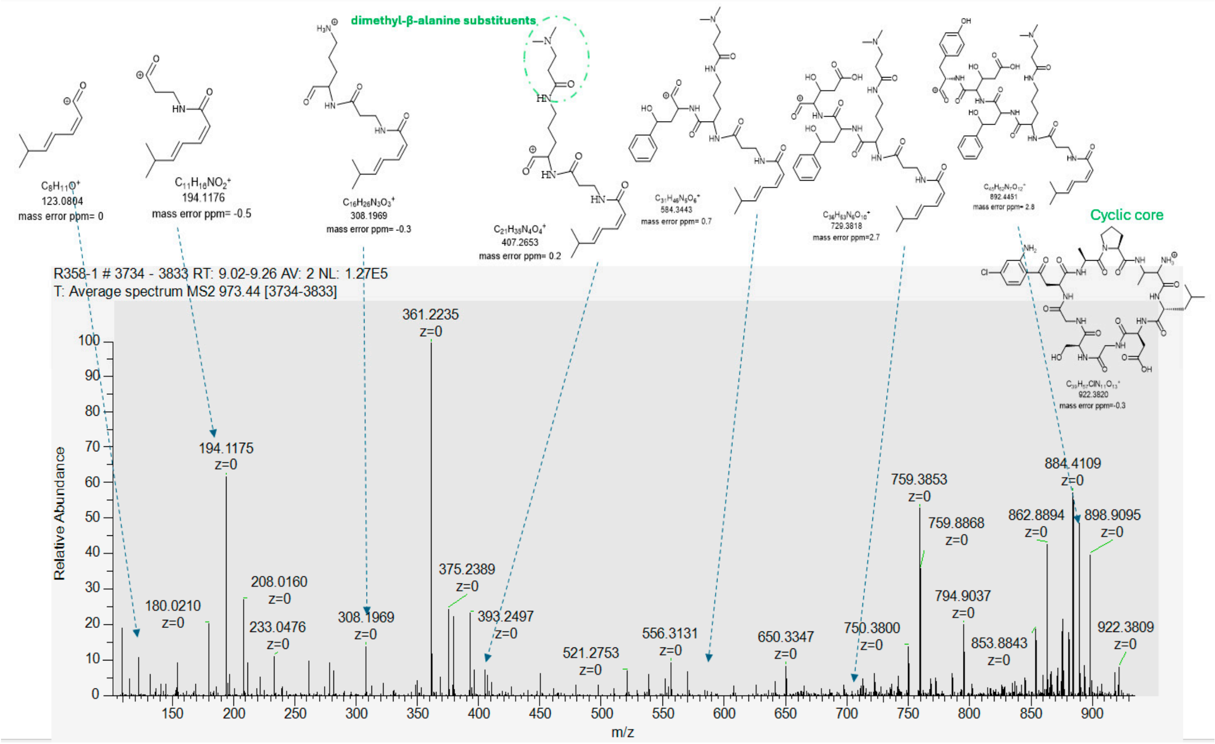

Figure S10. The tail fragmentation of putatively new molecules at m/z at m/z 1945.8621 corresponding to 973.4351 (M+2H)$^{+2}$ showing dimethyl-β-alanine attachment to ornithine of the gausemycin A analogue

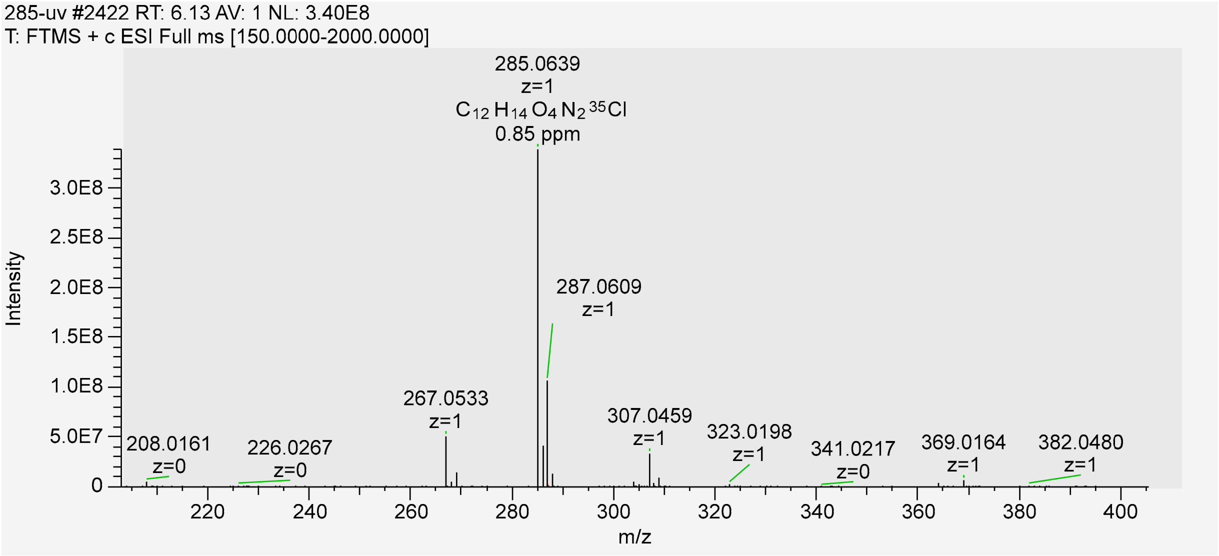

Figure S11. (+)-HRESIMS spectrum of **1**.

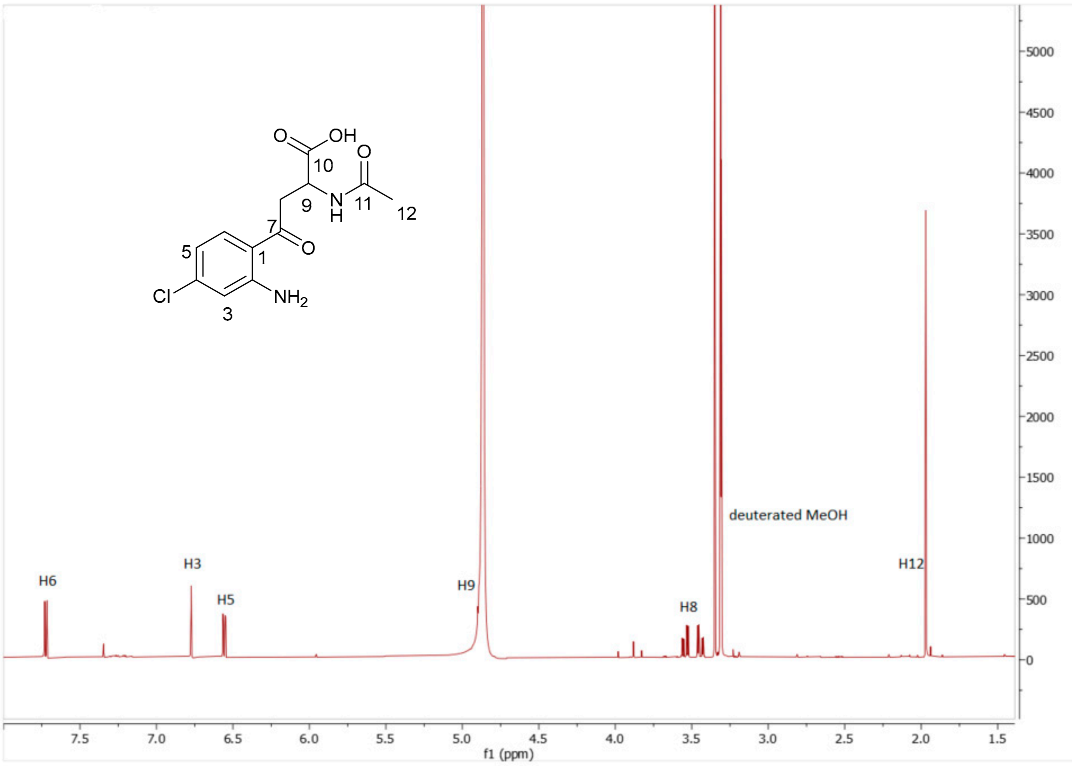

Figure S12. ^{1}H NMR spectrum of **1**.

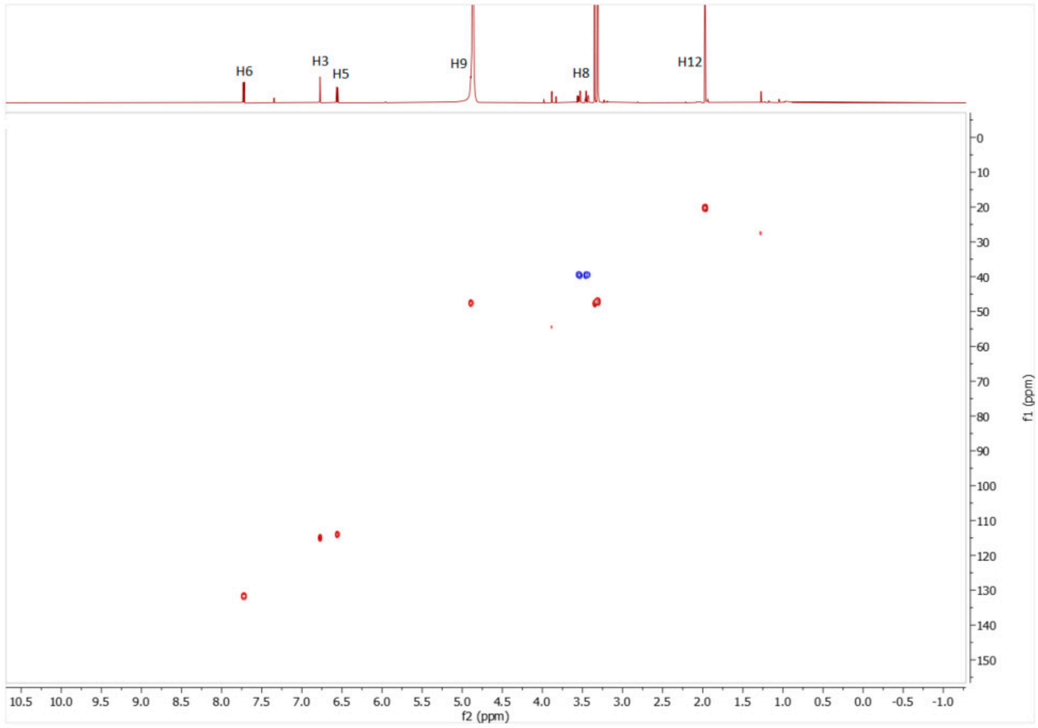

Figure S13. HSQC spectrum of **1**.

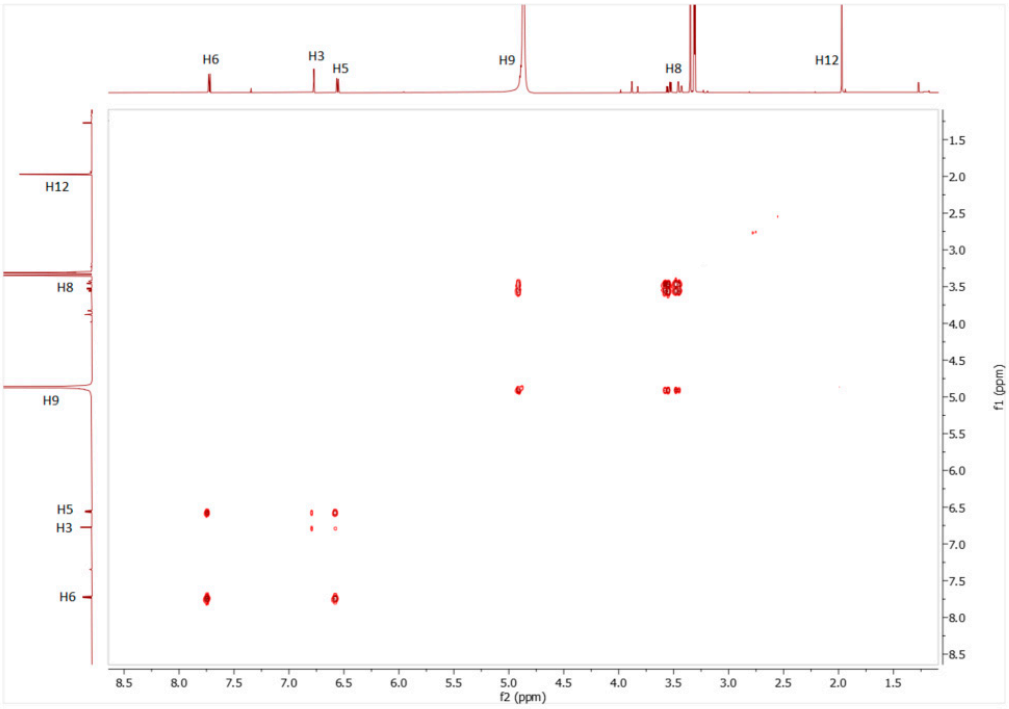

Figure S14. COSY spectrum of **1**.

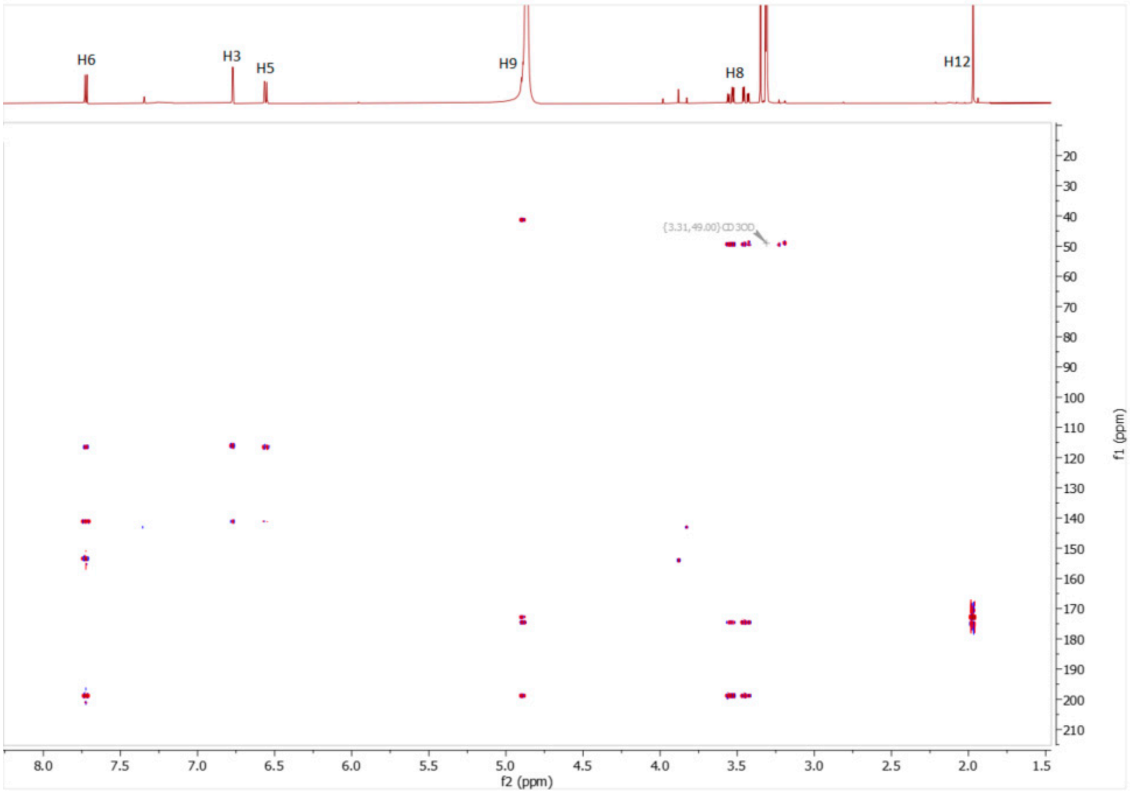

Figure S15. HMBC spectrum of **1**.

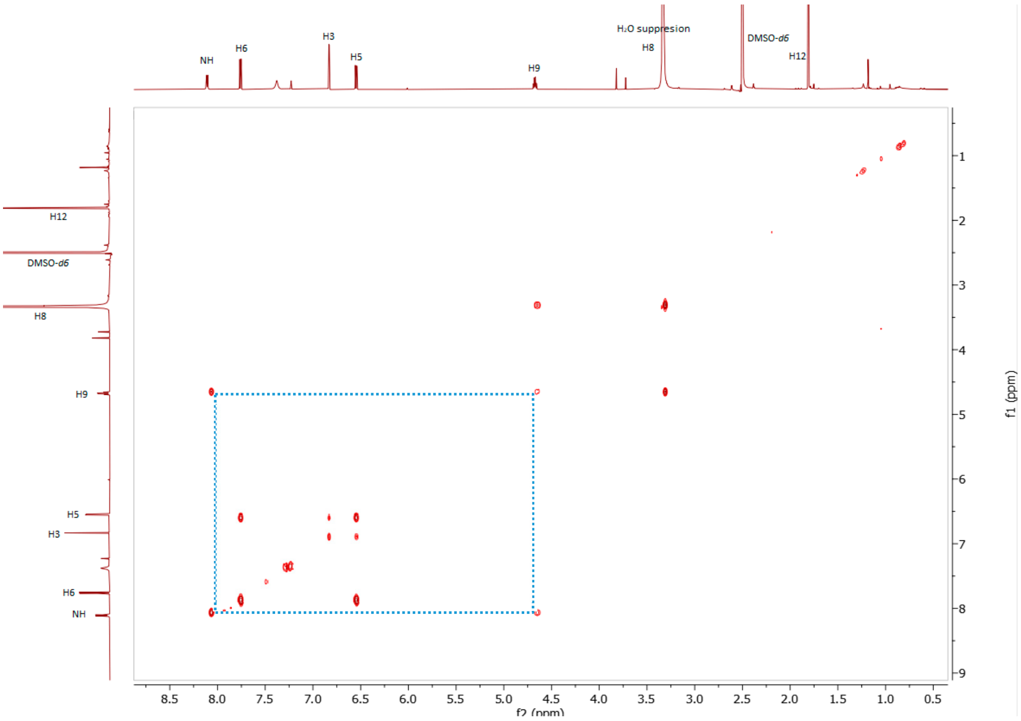

Figure S16. COSY spectrum of 1 in *DMSO*-d6

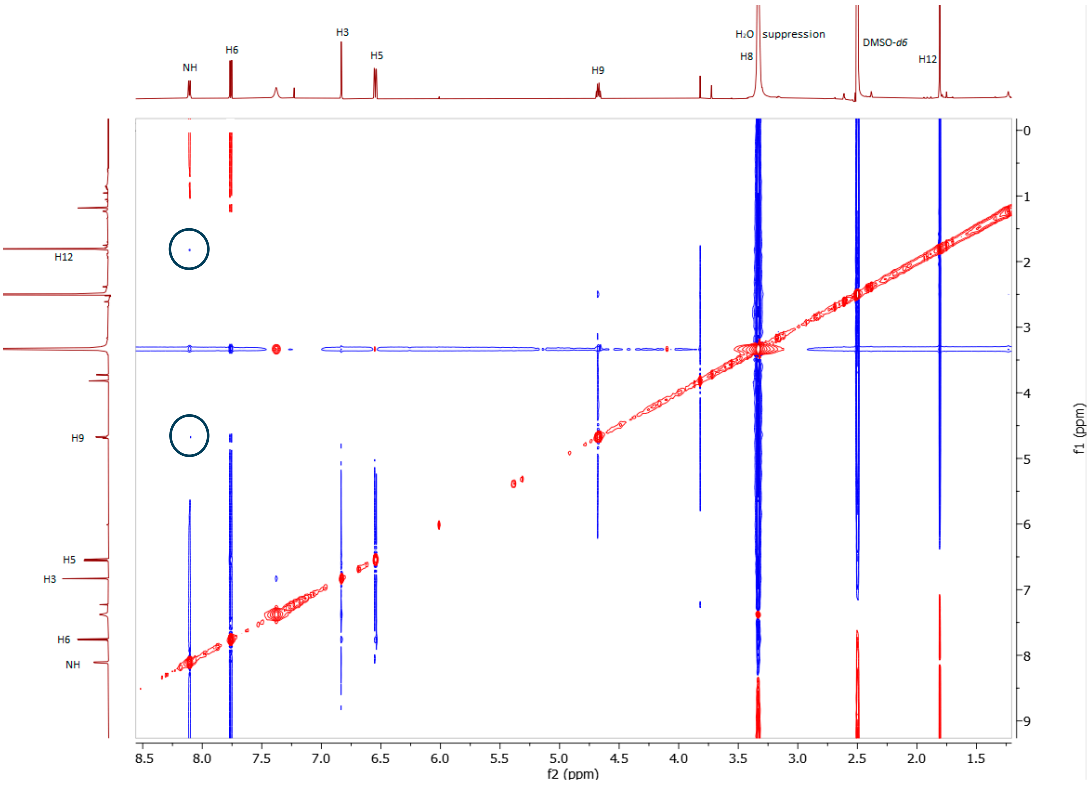

Figure S17. NOESY spectrum of **1** in *DMSO*-d6

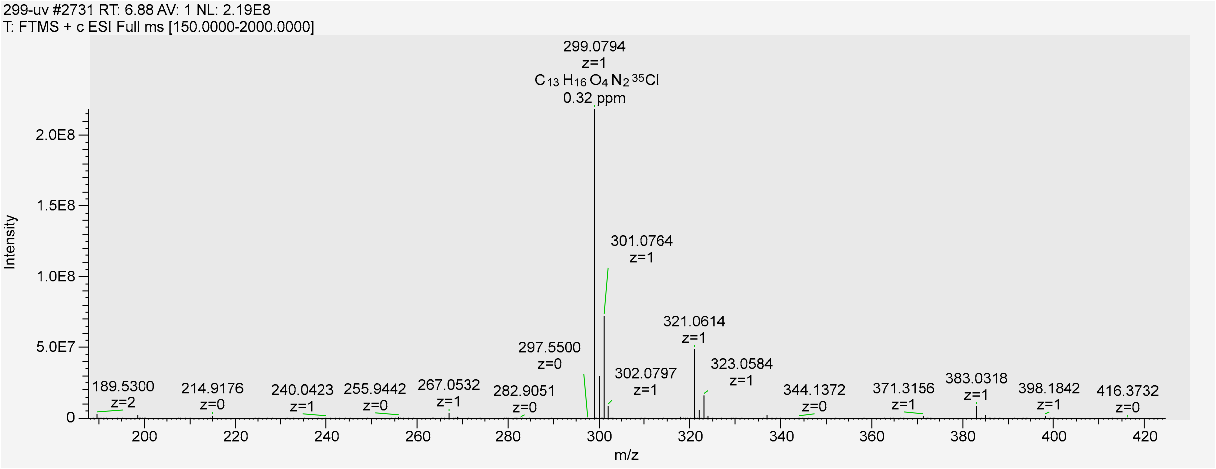

Figure S18. (+)-HRESIMS spectrum of **2**.

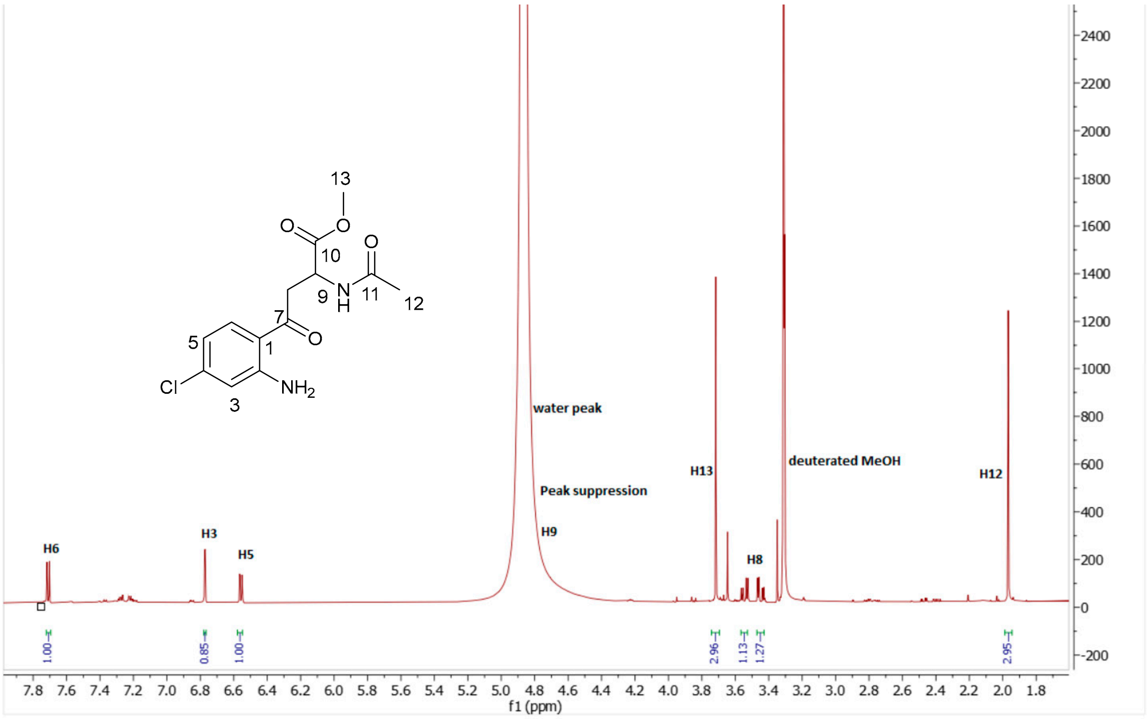

Figure S19. ^{1}H NMR spectrum of **2**.

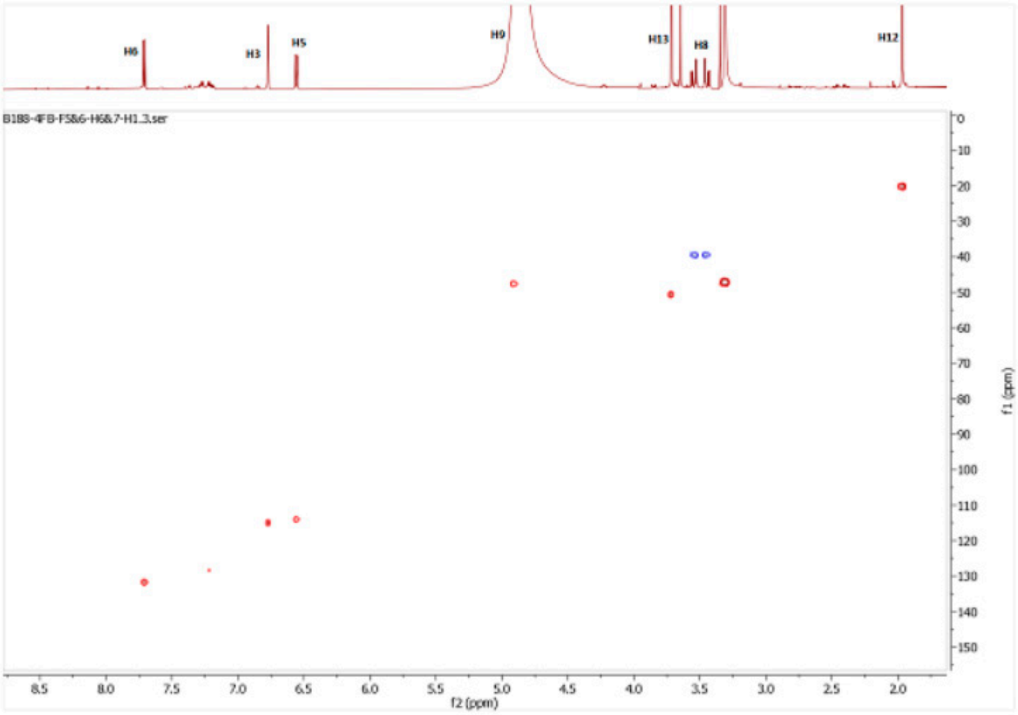

Figure S20. HSQC spectrum of **2**

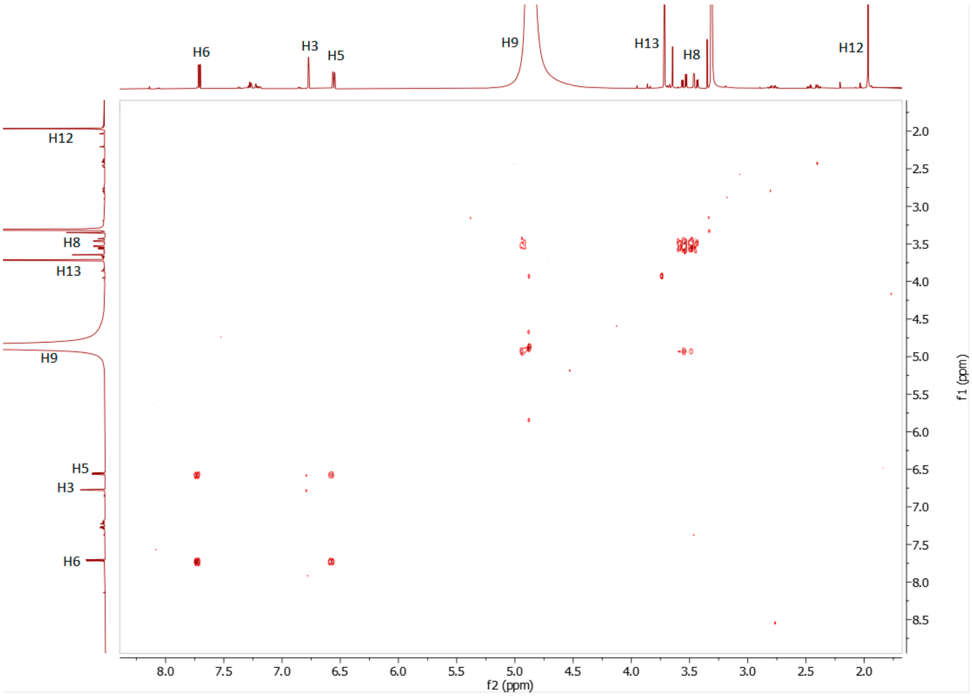

Figure S21. COSY spectrum of **2**.

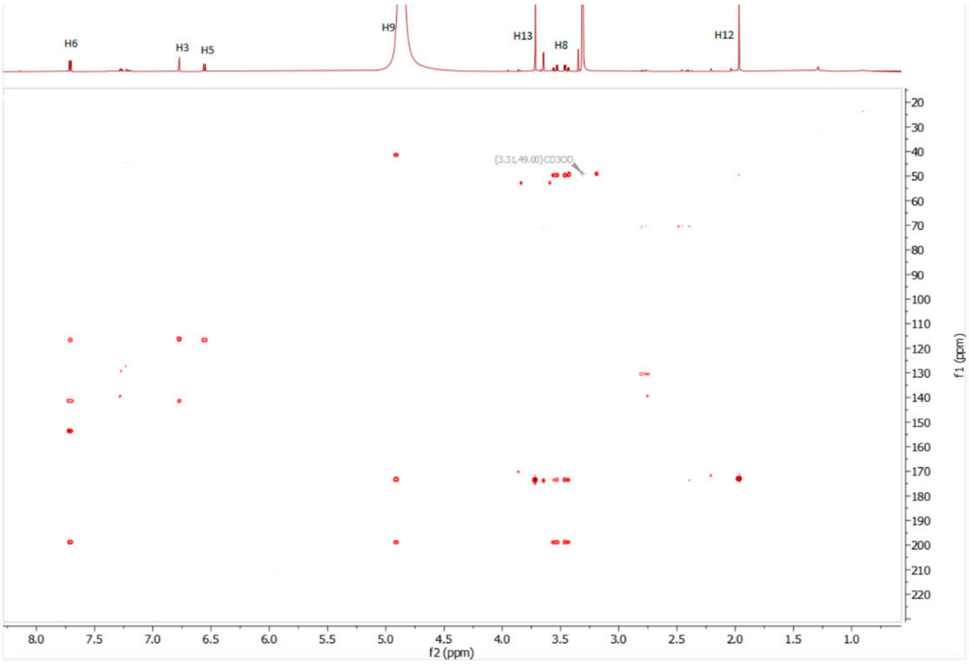

Figure S22. HMBC spectrum of **2**.

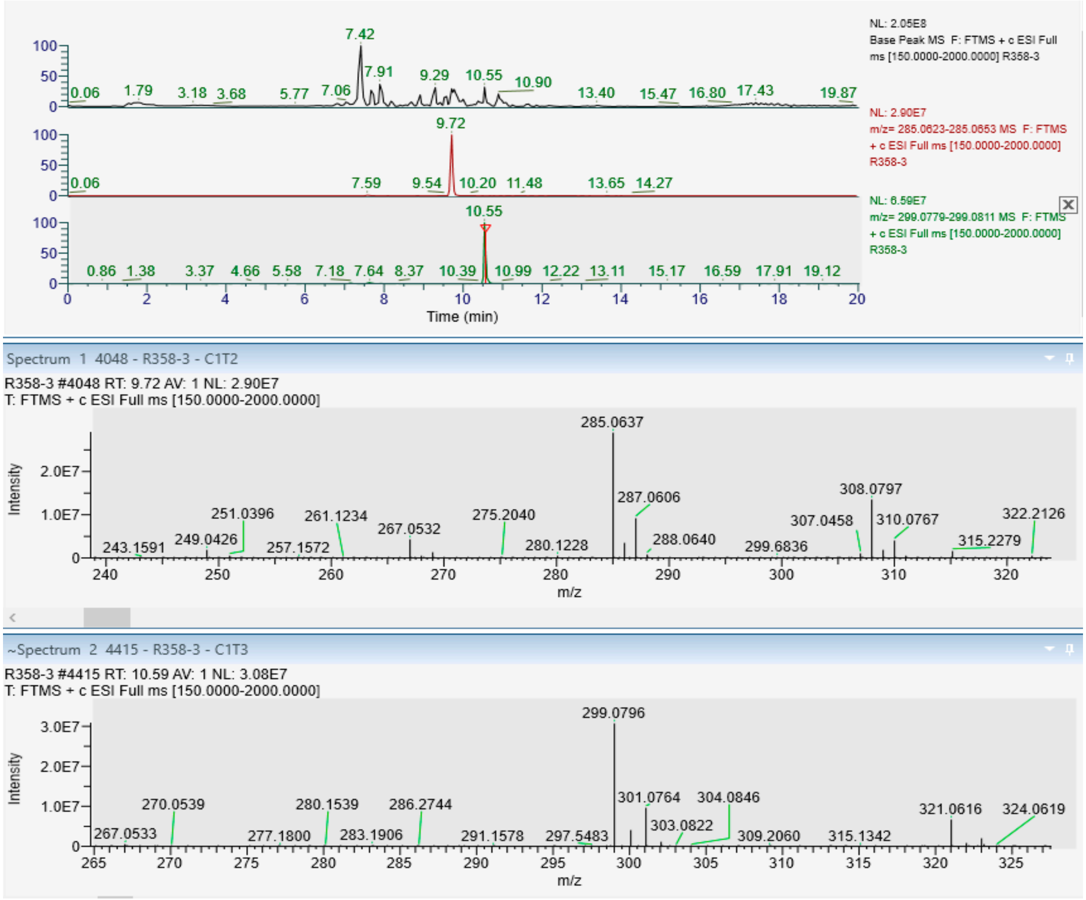

Figure S23. Extracted ion chromatogram and corresponding mass spectra of compound **1** and **2** in the butanol crude extract

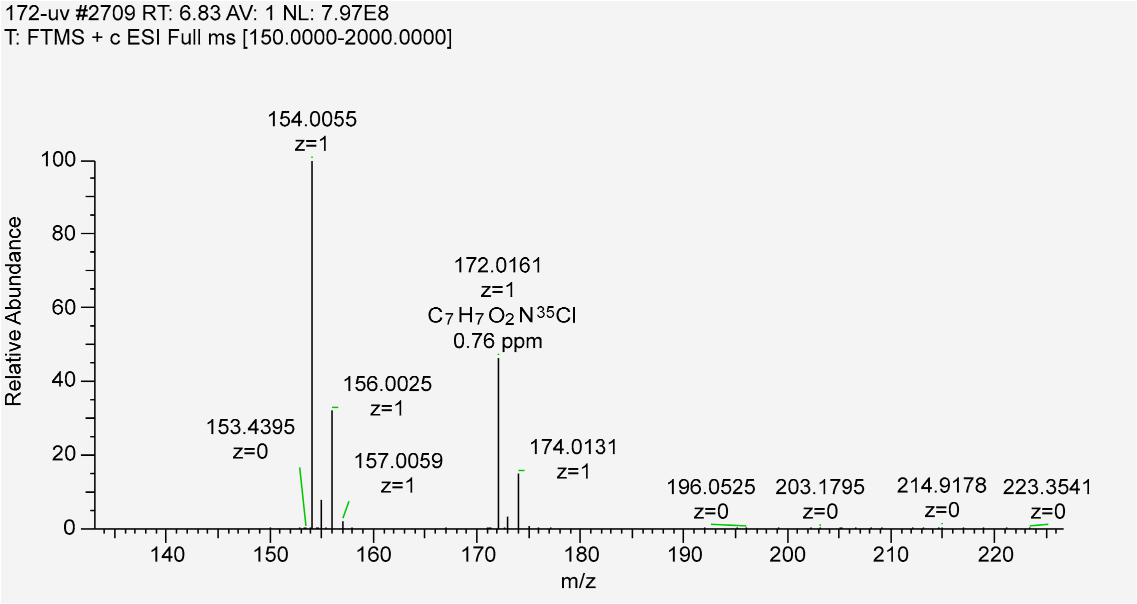

Figure S24. (+)-HRESIMS spectrum of **3**

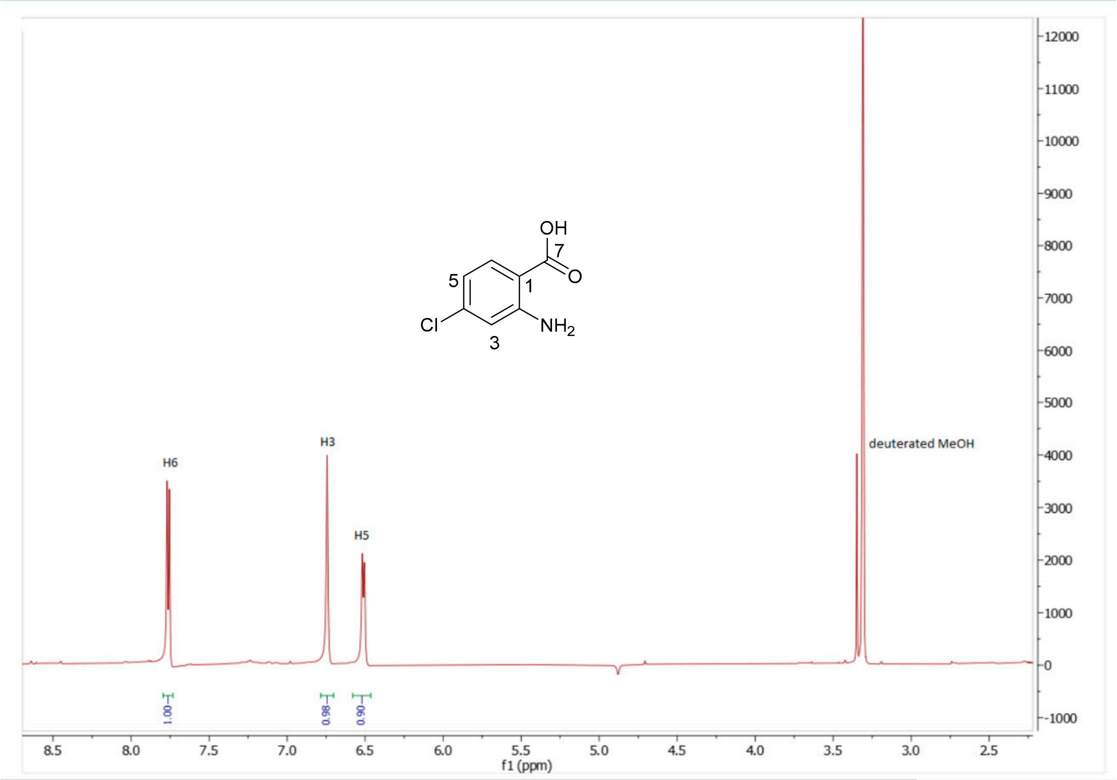

Figure S25. ^{1}H NMR spectrum of **3**.

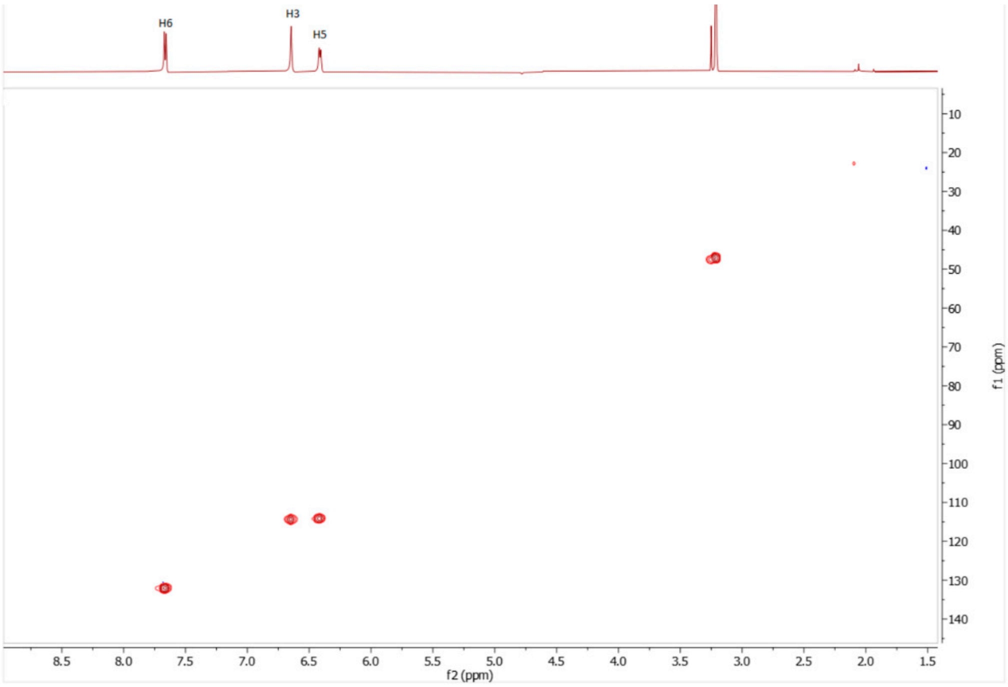

Figure S26. HSQC spectrum of **3**.

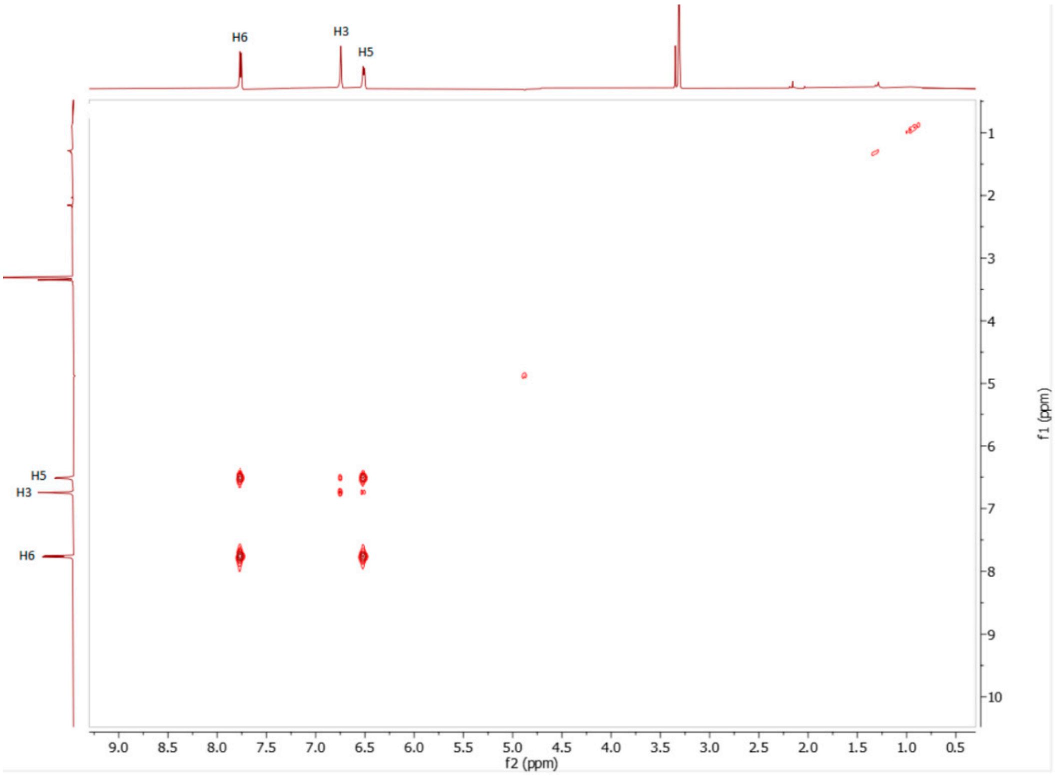

Figure S27. COSY spectrum of **3**

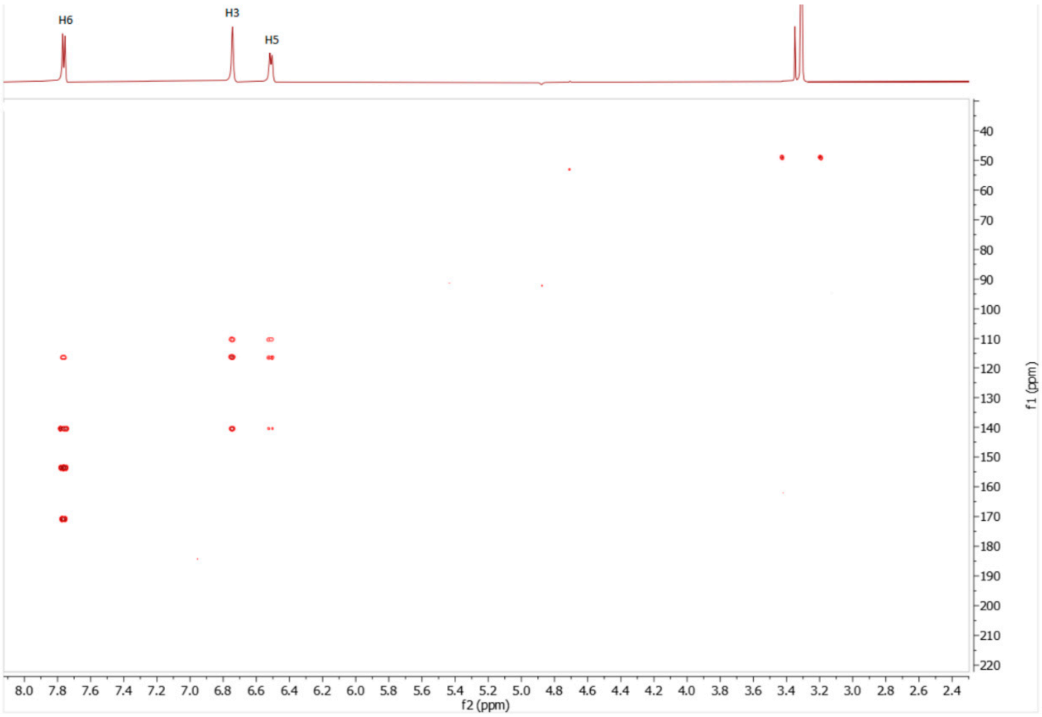

Figure S28. HMBC spectrum of **3**.

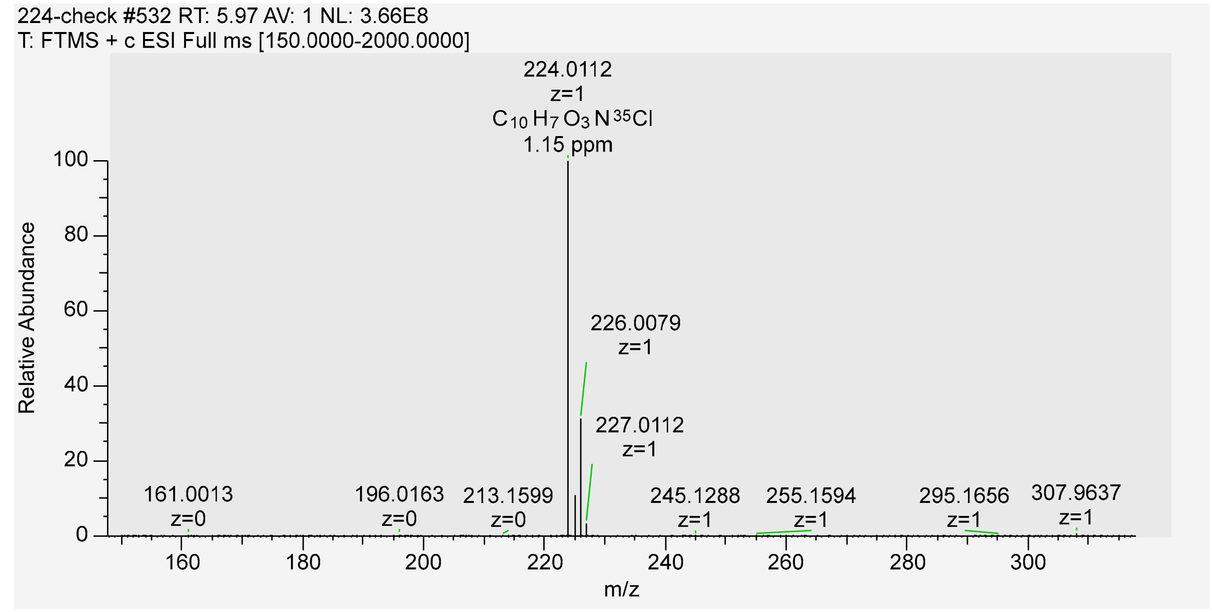

Figure S29. (+)-HRESIMS spectrum of **4**

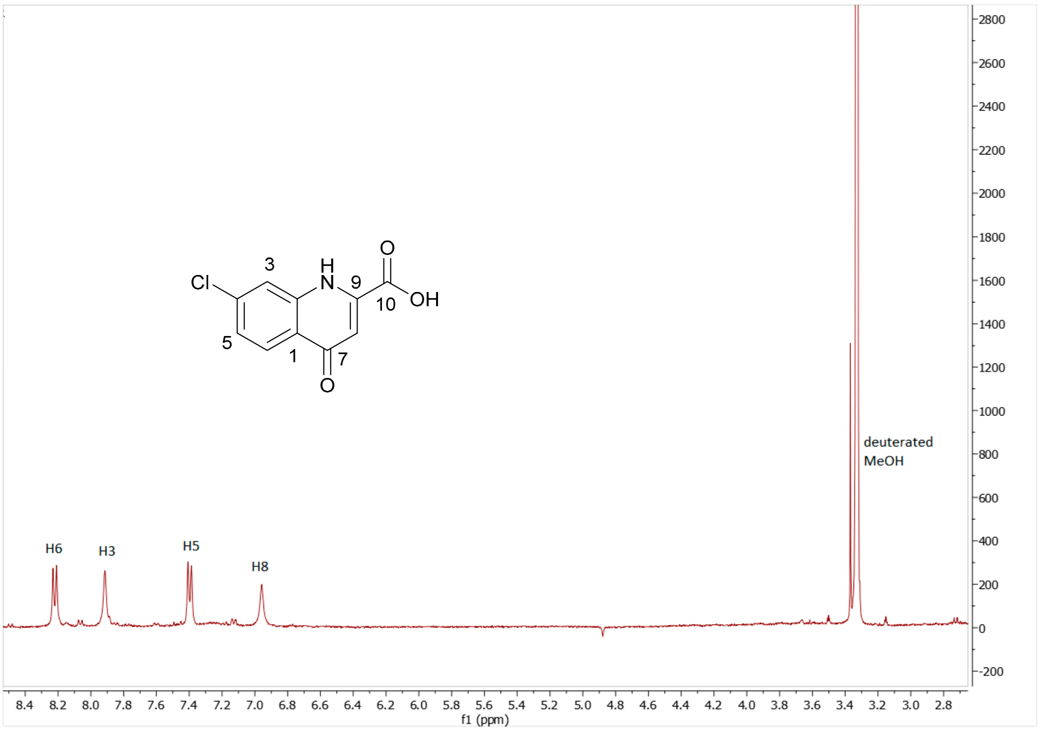

Figure S30. ^{1}H NMR spectrum of **4**.

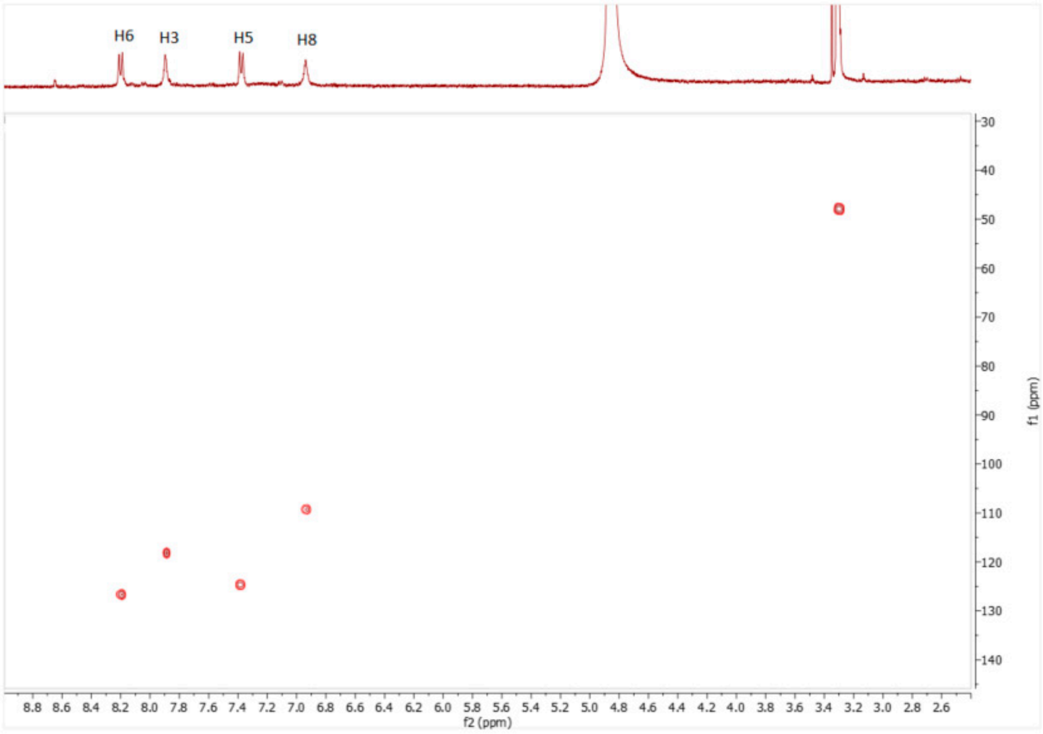

Figure S31. HSQC spectrum of **4**.

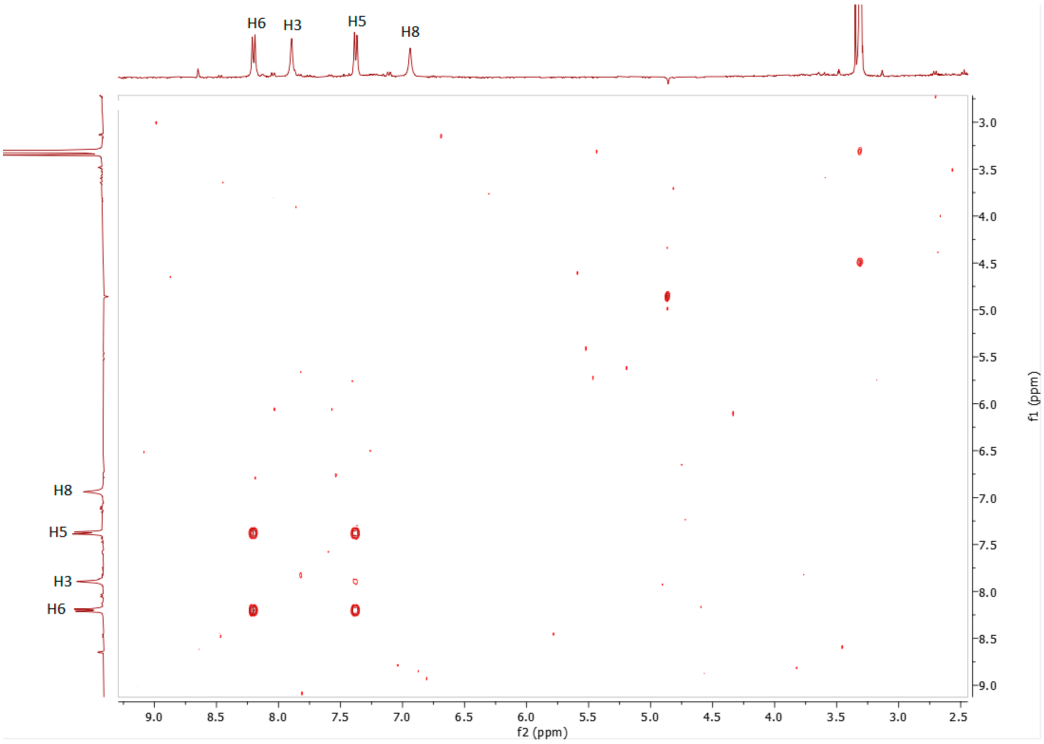

Figure S32. COSY spectrum of **4**.

F

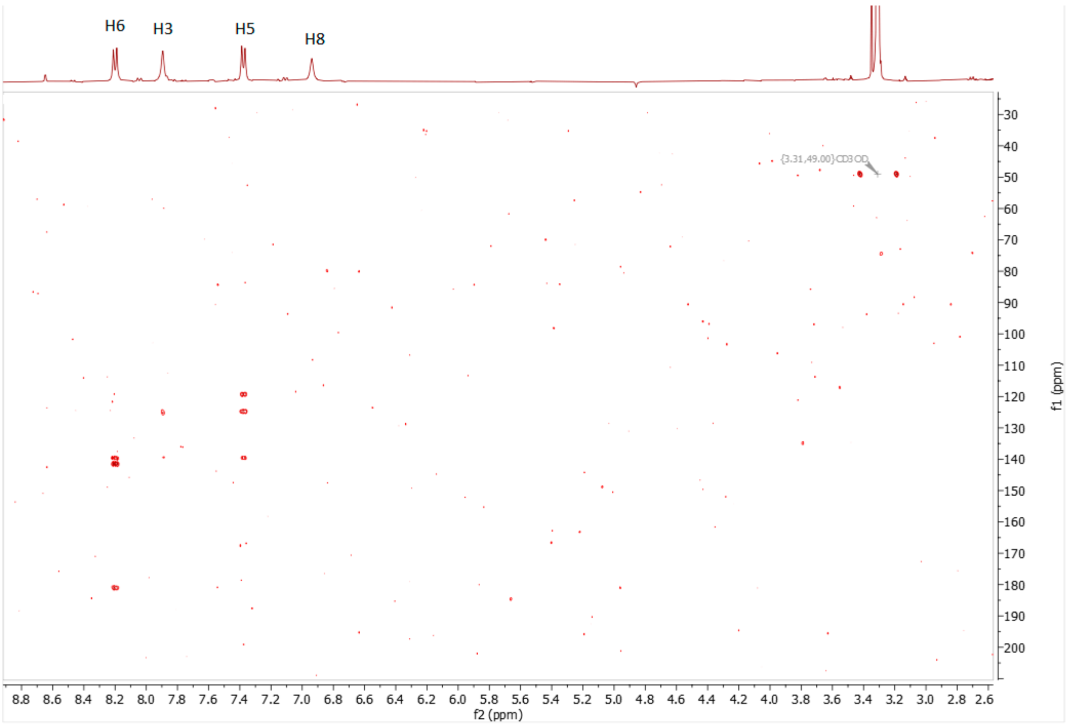

Figure S33. HMBC spectrum of **4**.

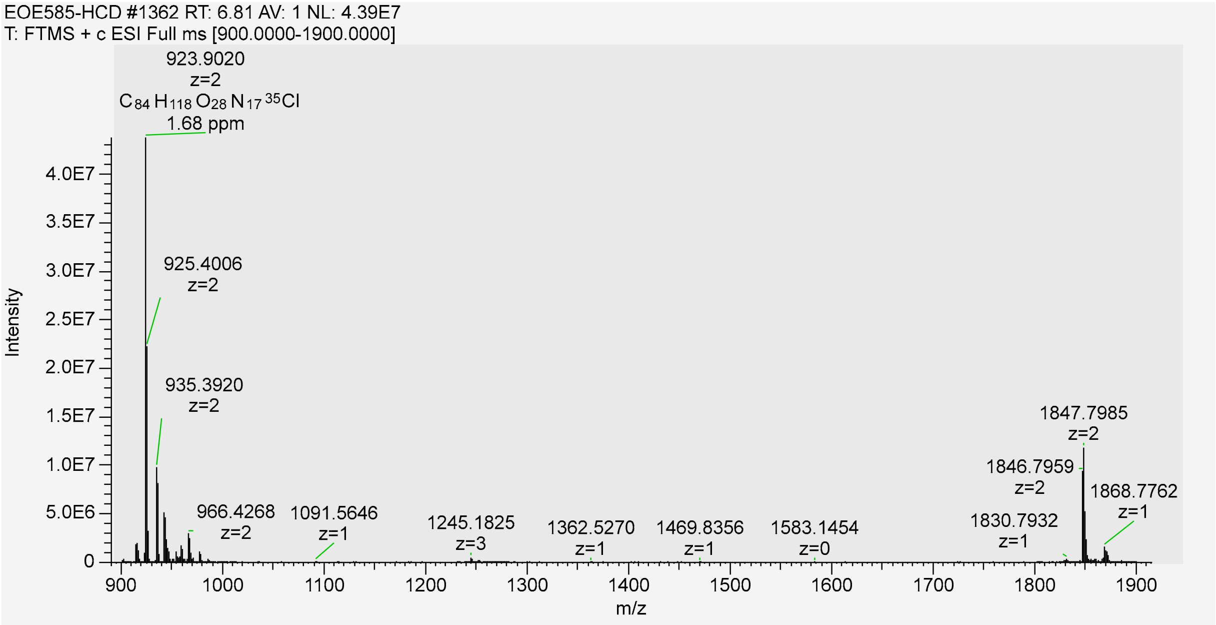

Figure S34. (+)-HRESIMS spectrum of **5**.

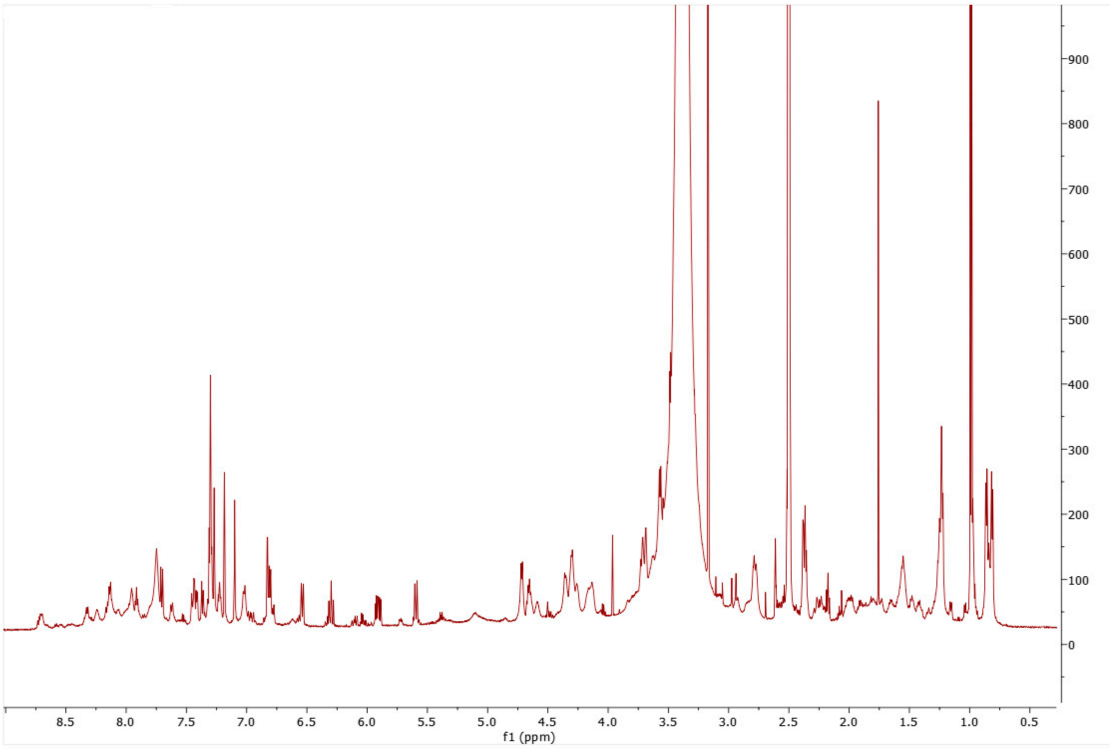

Figure S35. ^{1}H NMR spectrum of **5** at 45ºC in deuterated DMSO of 600 MHz.

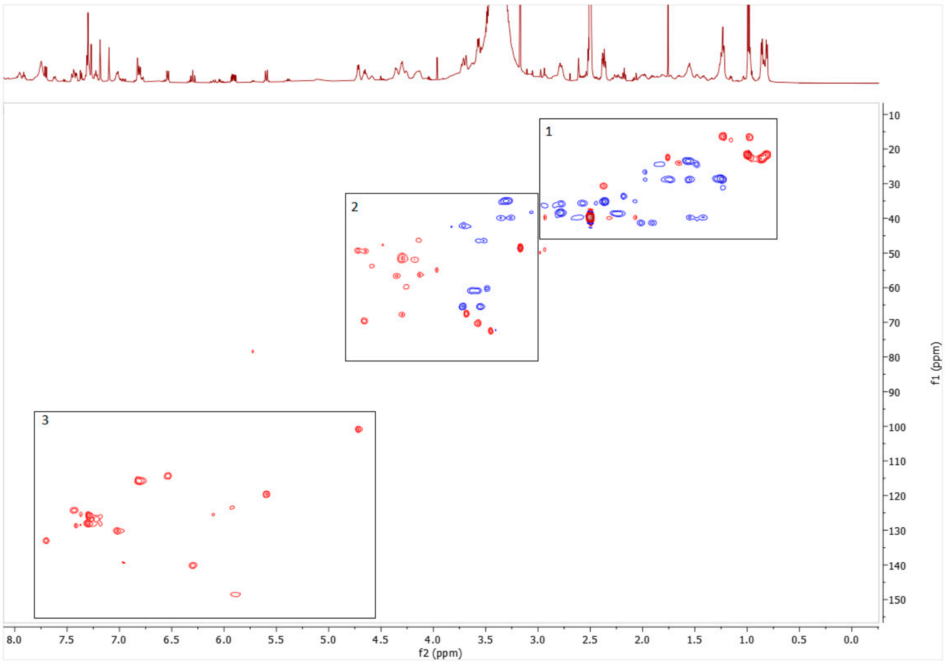

Figure S36. HSQC spectrum of **5** 45°C, DMSO, 800MH, chemical shift region (**1**-**3**).

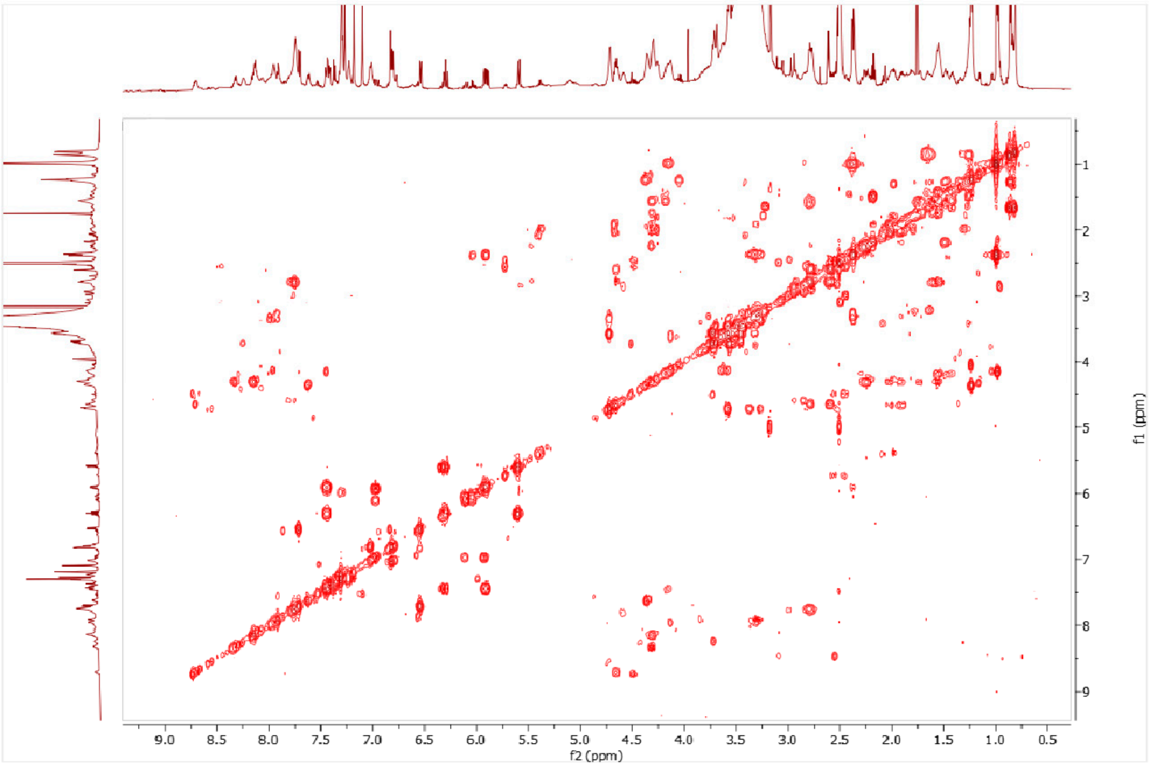

Figure S37. COSY spectrum of **5** at 45OC in deuterated DMSO of 600 MHz.

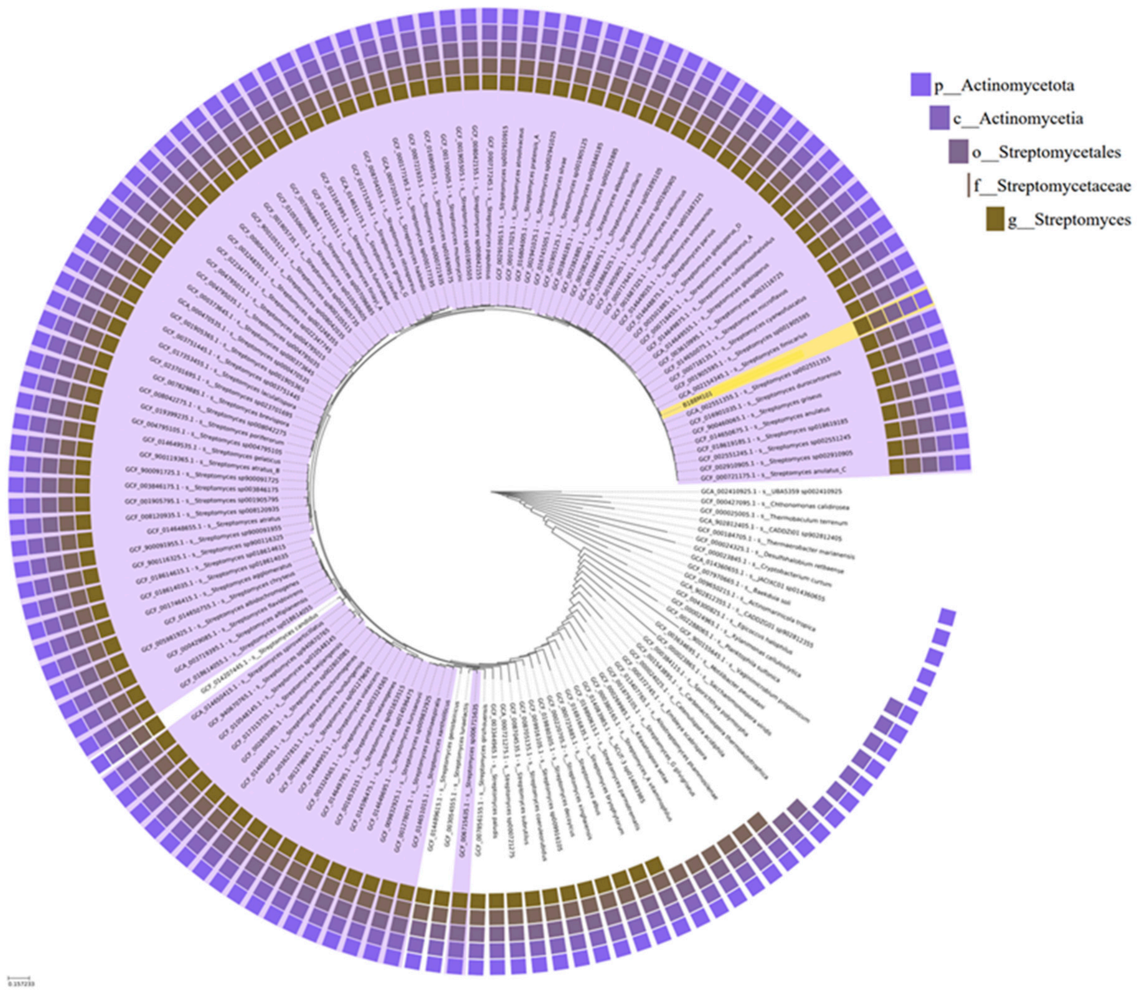

Figure S38. Phylogenetic characterisation of *Streptomyces* sp. B188M101 by the Genome Taxonomy Database (GTDB-Tk).

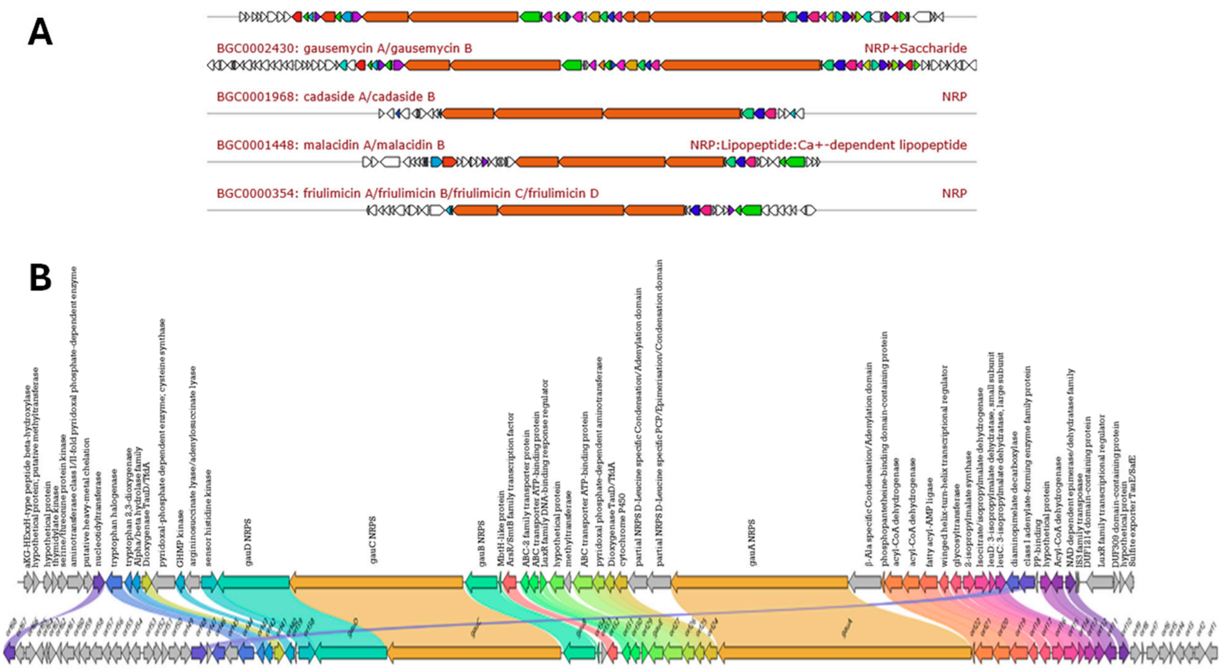

Figure S39. **(A)** Comparison of gausemycin producing BGC from *Streptomyces* sp. B188M101 with most closely related known clusters from the MIBiG database. **(B)** Alignment of gausemycin producing BGC from *Streptomyces* sp. B188M101 (top) and the gausemycin cluster from *Streptomyces* sp. INA-Ac-5812 (bottom), and functional annotation of the B188M101 cluster. Note: the cluster from *Streptomyces* sp. INA-Ac-5812 has been reversed in orientation for improved visualization of homology.

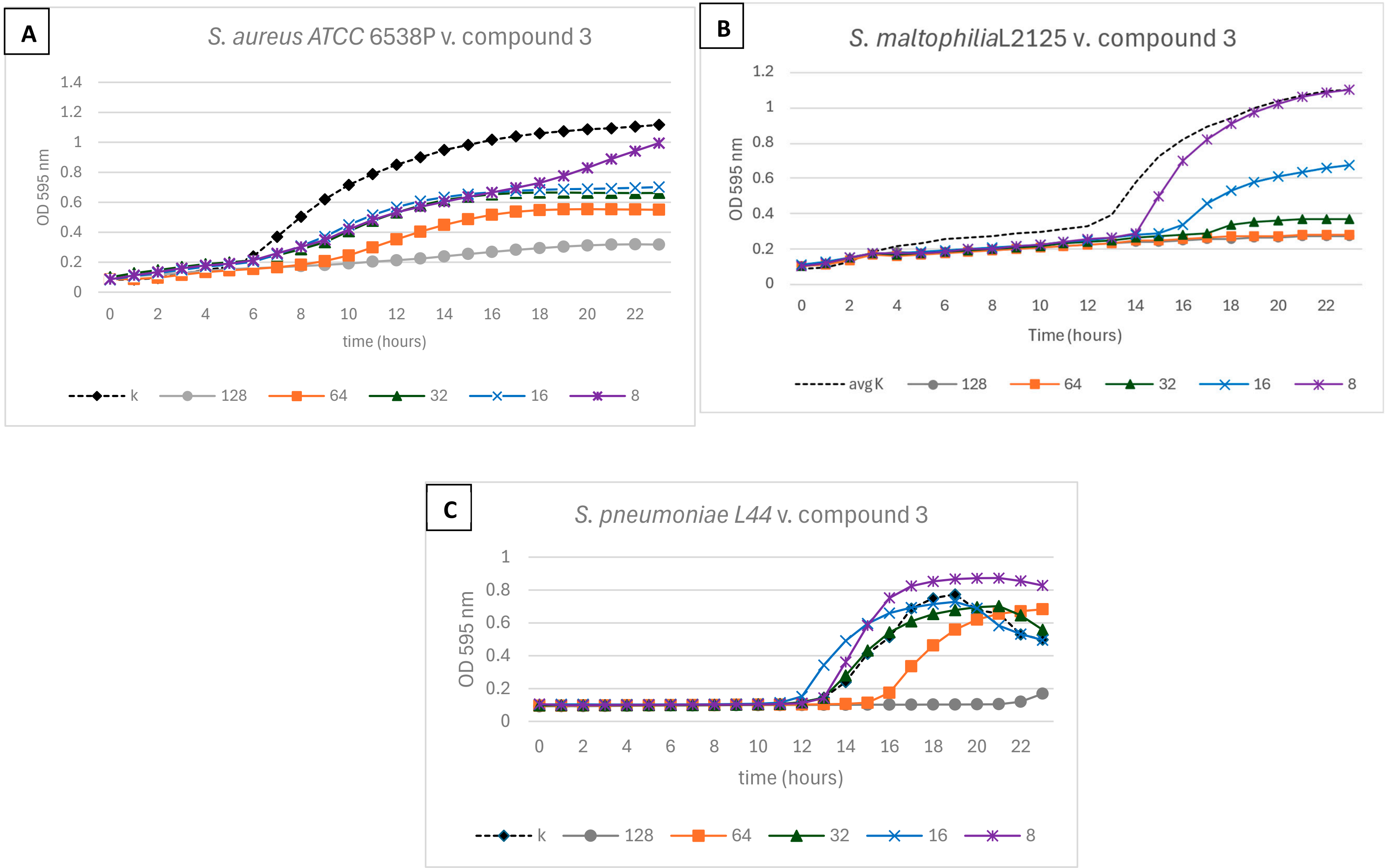

Figure S40. Growth curve inhibition of **3** against *S.aureus* ATCC6538P (**A**) , S. *maltophilia* L21259(**B**) and *S. pneumoniae* L44 (**C**)

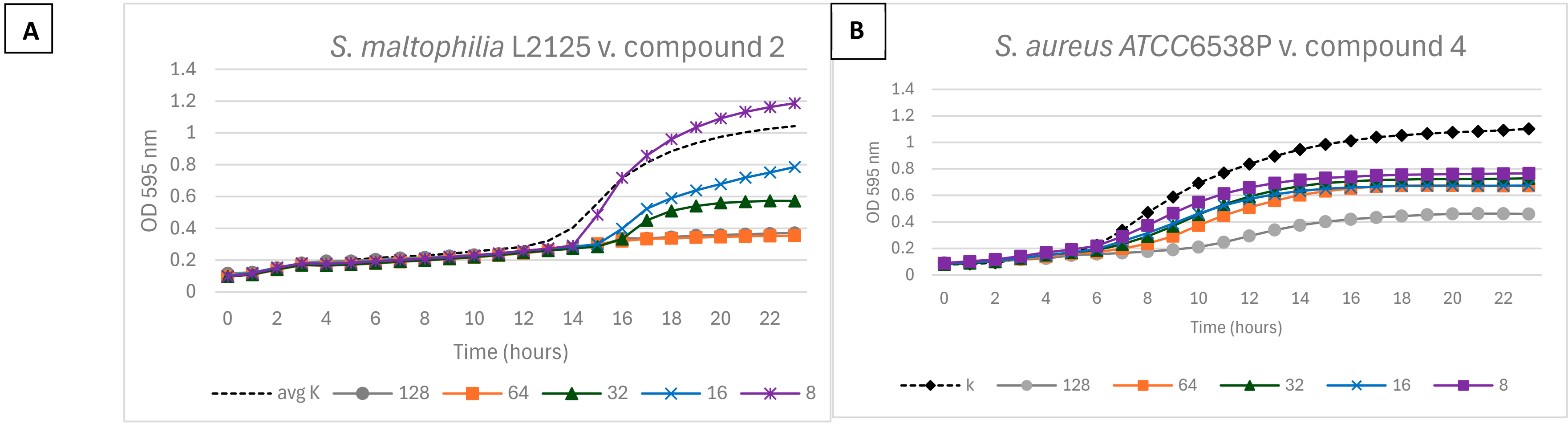

Figure S41. Growth curve inhibition of **2** and **4** against *S. maltophilia* L21259(**A**) and *S.aureus* ATCC6538P (**B**), respectively.

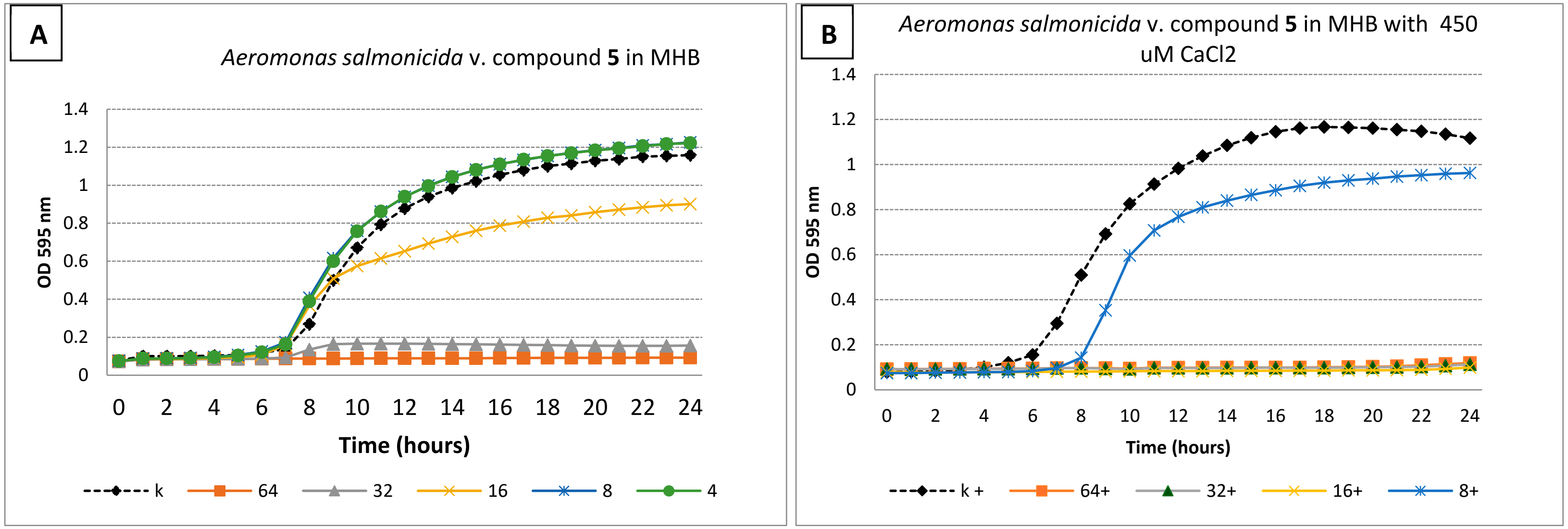

Figure S42. **(A)** Growth curve inhibition of **5** against *A. salmonicida* without $CaCl_2$ and **(B)** represents with $CaCl_2$ supplement.

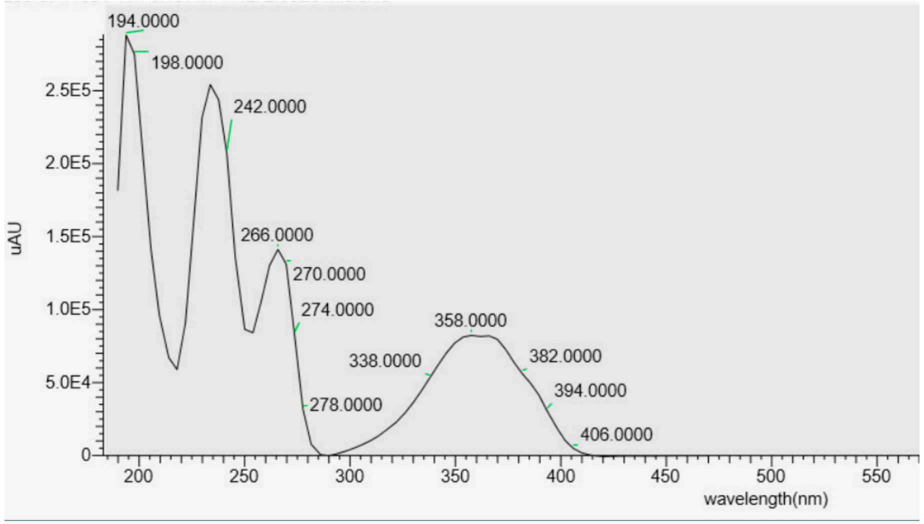

Figure S43. UV absorption of **1**

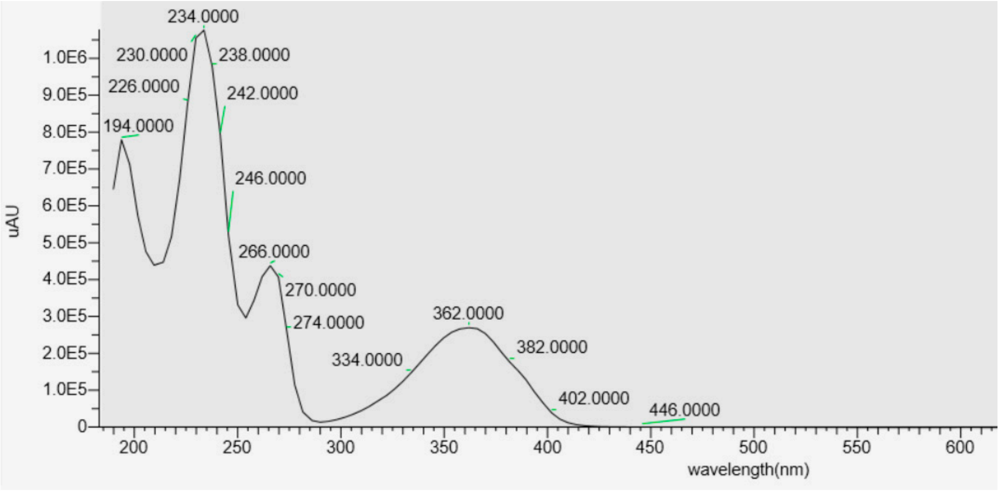

Figure S44. UV absorption of **2**

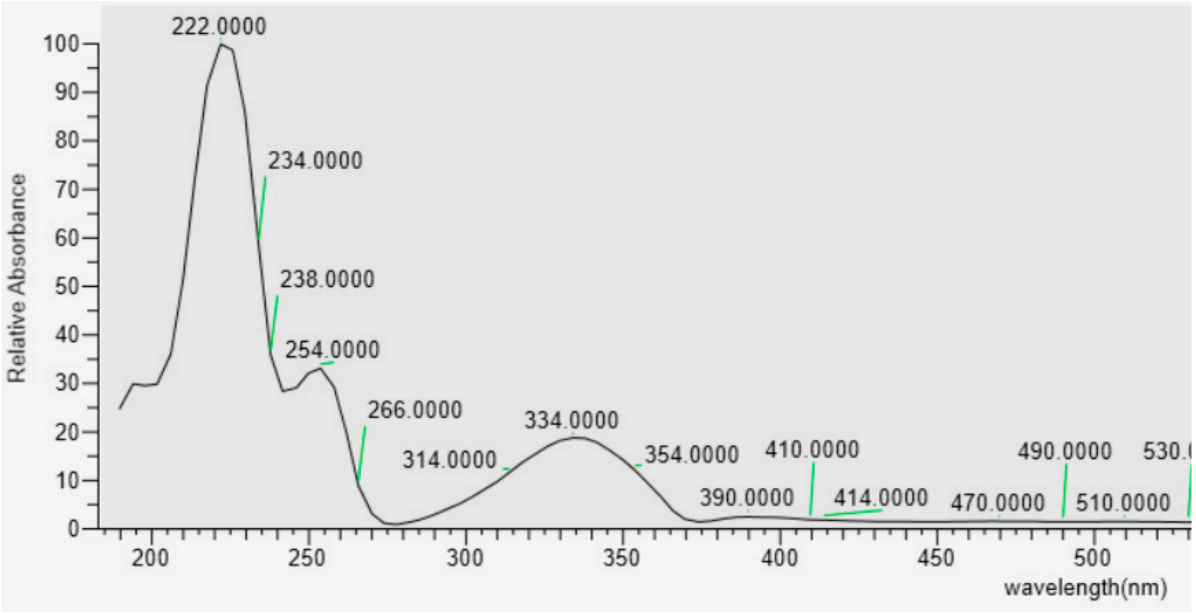

Figure S45. UV absorption of **3**

Table S1. Putative chemical dereplication of detected metabolites in the *Streptomyces* sp. B188M101_R358 extract

RT [min]	Observed m/z	MF	Theoretical mass	Error ppm	Putative compounds (Marinlit, *PubChem, Reaxys.com and NP Atlas*)
4.8	571.2085	$C_{33}H_{27}O_4N_6$	571.2088	-0.6	No hit
5.0	537.2236	$C_{30}H_{29}O_4N_6$	537.2245	-1.6	No hit
5.1	831.3432	$C_{26}H_{45}O_{17}N_{19}$	831.3429	-0.8	No hit
5.2	605.2120	$C_{17}H_{29}O_{11}N_{14}$	605.2135	-2.4	No hit
5.8	605.1929	$C_{36}H_{25}O_4N_6$	605.1932	-0.4	No hit
5.9	555.2133	$C_{33}H_{27}O_3N_6$	555.2133	-1.2	No hit
7.3	503.2871	$C_{26}H_{39}O_6N_4$	503.2864	1.5	No hit
7.4	398.1497	$C_{16}H_{24}O_5N_5S$	398.1493	0.9	No hit
7.7	517.3025	$C_{27}H_{41}O_6N_4$	517.3021	0.8	No hit
7.7	588.3399	$C_{30}H_{46}O_7N_5$	588.3392	1.2	No hit
7.8	602.3557	$C_{31}H_{48}O_7N_5$	602.3548	1.5	No hit
7.9	224.0109	$C_{10}H_7ClO_3N$	224.0109	0	Ageloline A
8.1	285.0638	$C_{12}H_{14}ClO_4N_2$	285.0607	0.5	No hit
8.1	485.2765	$C_{26}H_{37}O_5N_4$	485.2759	1.4	No hit
8.1	443.3232	$C_{22}H_{43}O_5N_4$	443.3228	1	No hit
8.1	556.3136	$C_{29}H_{42}O_6N_5$	556.3130	1.2	No hit
8.2	570.3295	$C_{30}H_{44}O_6N_5$	570.32861	1.5	No hit
8.2	531.3183	$C_{28}H_{43}O_6N_4$	531.3177	1.1	No hit
8.3	238.0267	$C_{11}H_9ClO_3N$	238.0266	0.6	No hit
8.3	370.3319	$C_{23}H_{48}O_3N$	370.3316	1	No hit
8.5	499.2919	$C_{27}H_{39}O_5N_4$	499.2915	0.8	No hit
8.6	584.3448	$C_{31}H_{46}O_6N_5$	584.3443	1.3	No hit
8.6	515.3234	$C_{28}H_{43}O_5N_4$	515.3228	1.2	No hit
8.7	823.4248	$C_{45}H_{57}O_8N_7$	823.4263	-1.8	No hit
8.8	172.0163	$C_7H_7ClNO_2$	172.0160	1.3	No hit
8.9	299.0795	$C_{13}H_{16}ClO_4N_2$	299.0793	0.5	No hit
8.7	923.9016[M+2H]	$C_{84}H_{118}ClO_{28}N_{17}$	923.9005	1.2	Gausemycin A

8.7	916.8937[M+2H]	$C_{83}H_{116}$ $ClO_{28}N_{17}$	916.8927	2.1	No hit
8.8	966.4280[M+2H]	$C_{88}H_{125}ClO_{29}N_{18}$	966.4269	0.6	Gausemycin C
8.8	959.4201[M+2H]	$C_{87}H_{123}ClO_{29}N_{18}$	959.4190	1.7	Gausemycin B
8.9	930.9088[M+2H]	$C_{85}H_{120}ClO_{28}$ N_{17}	930.9083	0.5	No hit
8.9	949.8827[M+2H]	$C_{85}H_{114}ClO_{29}0_{19}$	949.8797	2.8	No hit
9.0	937.9174[M+2H]	$C_{86}H_{123}ClO_{28}$ N_{17}	937.9161	0.9	No hit
9.0	973.4351[M+2H]	$C_{89}H_{126}ClO_{29}$ N_{18}	973.4347	0.4	No hit
9.1	956.8902[M+2H]	$C_{83}H_{118}ClO_{32}N_{18}$	956.8918	-1.7	No hit
9.1	857.8801[M+2H]	$C_{79}H_{110}ClO_{24}N_{17}$	857.8794	0.8	Gausemycin D
9.2	980.4431[M+2H]	$C_{90}H_{129}ClO_{29}$ N_{18}	980.4425	0.6	No hit
9.3	864.8876[M+2H]	$C_{80}H_{112}ClO_{24}N_{17}$	864.8872	0.5	No hit
9.3	871.8955[M+2H]	$C_{81}H_{114}ClO_{24}N_{17}$	871.8957	0.9	No hit
9.4	944.9249[M+2H]	$C_{87}H_{124}ClO_{28}N_{17}$	944.9240	1.3	No hit
9.5	922.9116[M+2H]	$C_{85}H_{120}ClO_{27}N_{17}$	922.9109	1.7	No hit
**9.5**	856.8809[M+2H]	$C_{80}H_{112}ClO_{23}N_{17}$	856.8897	1.4	No hit
9.6	878.9034[M+2H]	$C_{82}H_{116}O_{24}N_{17}Cl$	878.9028	0.6	No hit
9.6	863.8989[M+2H]	$C_{81}H_{114}O_{23}N_{17}Cl$	863.8976	1.7	No hit
10.3	558.4222	$C_{28}H_{56}O_6N_5$	558.4225	-0.5	No hit
10.5	485.3341	$C_{24}H_{45}O_6N_4$	485.3334	1.5	No hit
10.9	616.4638	$C_{31}H_{62}O_7N_5$	616.4644	-0.9	No hit
11.4	674.5053	$C_{34}H_{68}O_8N_5$	674.5062	-1.4	No hit

Table S2. ^{1}H and ^{13}C NMR data of **1** (CD$_3$OD, 600 MHz)

Atom	δ^{13}C, mult.	δ^{1}H (mult., *J* in Hz)	COSY (H→H)	HMBC (H→C)
1	116.7, C			
2	153.3, C			
**3**	116.9, CH	6.77 (d, 2.2)	5	1, 4, 5
4	141.0, C			
5	115.9, CH	6.56 (dd, 8.7, 2.2)	3, 6	1, 3, 4w
6	133.6, CH	7.72 (d, 8.7)	5	1, 2, 4, 7
7	198.1, C			
8	41.2, CH$_2$	3.54(dd, 17.3, 6.3), 3.45 (dd, 16.9, 6.3)	9	7, 9, 10
9	49.3, CH	4.89 (m)	8	7 ,8, 10, 11
10	173.1, C			
11	172.9, C			
12	21.8, CH$_3$	1.97(s)		11

Table S3. ^{1}H and ^{13}C NMR data of 2 (CD$_3$OD, 600/150MHz)

Position	δ^{13}C, mult.	δ^{1}H (mult., *J* in Hz)	COSY (H→H)	HMBC (H→C)
**1**	116.7, C			
**2**	153.3, C			
**3**	116.9, CH	6.77 (d, 2.2)	5	1, 5
**4**	141.0, C			
**5**	115.9, CH	6.56 (dd, 8.7, 2.2)	3, 6	1, 3, 4w
	133.6, CH	7.71 (d, 8.7)	5	2, 3, 4, 7
**7**	198.1, C			
**8**	41.2, CH$_2$	3.53(dd, 17.3, 6.3), 3.46 (dd, 16.9, 6.3)	9, 8	7, 9, 10
**9**	49.3, CH	4.91 (m)	8	7, 8, 10, 11
**10**	173.1, C			
**11**	172.9, C			
**12**	21.8, CH$_3$	1.96(s)		11
**13**	51.5, OCH$_3$	3.65 (s)		10

Table S4. ^{1}H and ^{13}C NMR data of **3** (CD_3OD, 600/150 MHz)

Position	δ^{13}C, mult.	δ^{1}H (mult., *J* in Hz)	δ^{13}C, mult.[1]	δ^{1}H (mult., *J* in Hz)[1]
1	125.0, C		125.0, C	
2	141.4, C		141.7, C	
3	119.3, CH	7.89(s)	119.6, C	7.90 (d, 1.9)
4	139.7, C		139.7, C	
5	125.4, CH	7.38 (d, 8.7)	125.7, C	7.36 (dd, 8.7)
6	127.7, CH	8.20 (dd, 8.7)	128.0, C	8.19 (d, 8.7)
7	181.0, C		181.5, C	
8	109.5, CH	6.94 (s)	110.0, C	6.94 (s)
9	-		148.4, C	
10	167.9, C			

Carbon in position C2 was not observed due to lower intensity resulting from quaternary carbon or poor relaxation[2].
Ageloline A NMR data was acquired using

Table S5. ^{1}H and ^{13}C NMR data of **4** (CD_3OD, 600/150 MHz)

Position	δ^{13}C, mult.	δ^{1}H (mult., *J* in Hz)	COSY (H→H)	HMBC (H→C)
1	110.3, C			
2	153.6, C			
3	116.3, CH	6.74 (d, 2.2)	5	1, 5
4	140.5, C			
5	*116.0, CH*	6.50(dd, 8.7, 2.2)	3, 6	1, 3w, 4
6	133.9, CH	7.77 (d, 8.7)	5	2, 3, 4, 7
7	170.8, C			

Table S6. ^{1}H and ^{13}C NMR data of **5** (DMSO-*d6*, 600/150 MHz).

Amino acids	Position	δH	δc	Amino acid	Position	δH	δc
**β-Ala-1**	α	4.36	47.8		γ	1.64	23.9
	ß	1.23	16.5		δ	0.87/0.87	22.9/21.6
	C′		171.2		C′		177.1
	NH	7.63			NH	8.26	
**L-Orn-2**	α	4.30	52.6	**L-Asp-8**	α	4.61	53.7
	ß	1.75/1.56	28.7		β	2.58/ 2.78	35.7
	γ	1.55	23.6		γ		171.5
	δ	2.79	38.4		C′		171.2
	C′		171.7		NH	8.71	
	NH	7.76		**Gly-9**	α	3.83	42.6
**Ahpb-3**	α	4.3	51.6		C′		175
	ß	2.02/1.92	41.3		NH	8.25	
	γ	4.67	69.6	**L-Ser-10**	α	4.13	56.4
	δ		145.3		ß	3.64/3.57	60.9
	ε	7.31	125.6		C′		172.1
	ζ	7.31	127.9		NH	7.96	
	η	7.23	126.7	**Gly-11**	α	3.73/3.69	42.3
	C′		172.7		NH	8.25	
	NH	8.70			C′		172.4
**hGlu-4**	α	4.35	56.6	**ClKyn-12**	α-1	4.73	49.2
	ß	4.32	67.7		ß-2	3.36/3.26	40.1
	γ	2.25	38.7		γ-3		197.4
	δ		171.7		δ -4		115.6
	C′	-			NH	7.97	

	NH	7.62			ε^{1}	7.71	133.6
**Tyr-5**	α	4.60	53.8		ε^{2}		152.1
	ß	2.93/2.85	36.5		ζ^{1}	6.54	114.9
	γ		139		ζ^{2}	6.82	116.3
	δ	7.03	130.3		η		139.4
	NH	7.77			C′		170.9
	ε	6.84	116.1	**L-Ala-13**	α-1	4.36	47.8
	ζ		156.4		ß-2	1.23	16.5
	C′		171.9		NH	7.63	
**Dab-6**	α	4.30	51.6		C′		171.2
	ß	4.13	46.4	**L-Pro-14**	α	4.28	59.9
	γ	0.99	16.5		ß	1.99/1.79	28.7
	NH	`	7.46		γ	1.91	24.5
	NH $^{\gamma}$		8.14		δ	3.58/3.51	46.4
	C′		170.9		C′		171.1
**D-Leu-7**	α	4.18	51.9	**Fatty acid chain**	α	5.59	119.5
	ß	1.54/1.44	39.7		β	6.30	140.1
	γ	7.44	124.2		γ	7.44	124.2
	δ	5.89	148.5		δ	5.89	148.5
	ε	2.37	30.9		б	2.37	30.9
	ζ	0.99	22.1		ς	0.99	22.1
	C′		166.1		ς		166.1
**Arabinose [sugar moiety]**					α-1	4.72	
					ß-2	3.57	70.3
					γ-3	3.45	72.5
					δ -4	3.68	67.4
					ε-5	3.71/3.54	65.4

TableS7. MS^2 fragmentation sequences of compound **5** using Orbitrap HR-ESI-MS

Structural arrangement	Observed fragment ions m/z	Ion mapped m/z	Neutral loss m/z (error ppm)	Sequence of observed mass ions (amino acid/ non-amino acid loss)
Tail	1714.7493	1846.7947	132.0467	Arabinose
	194.1175		194.1175	fatty acid chain+ β-Ala1
	308.1969	194.1175	114.0794	Orn2
	485.2758	308.1969	177.0805	Ahpb3 observed at C-terminal
	1230.4811	1407.5615	177.0805	(Ahpb3)-observed at N-terminal
	1085.4444	1230.4811	145.0369	hGlu4
	922.3811	1085.4444	163.0631	Tyr5
Core	851.3453	922.3811	71.0384	Ala13
	627.3111	851.3453	224.0337	Cl-Kyn12
	570.2895	627.3111	57.0215	Gly11
	483.2556	570.2895	87.0320	Ser10
	426.2349	483.2556	57.0215	Gly9
	408.2238	426.2349	18	H_2O loss
			408.2238	Asp8-Leu7-Dab6-Pro14

Table S8. Marfey's derivatisation analysis of compound **5** hydrolysates.

Derivatised Amino acid	**Compound 5** retention time	L Retention time	D Retention time	DL Retention time
Alanine	23.901	24.091	26.011	
Serine	18.365	18.556	17.907	
Leucine	33.513	30.406	33.407	
Aspartic acid	19.291	19.624	20.389	
Ornithine	29.333			D-27.866, L-29.493
Proline	23.532	23.062	24.095	
Tyrosine	34.00	35.302		
Glycine	22.014	22.419		
β-alanine	27.420	27.653		

Table S9: Annotation of open reading frames (ORFs) in the NRPS/betalactone biosynthetic gene cluster, from the genome of *Streptomyces* sp. B188M101.

ORF	Contig annotation	Length (nt)	Length (AA)	Orient-ation	Product
orf1	ctg_588	909	302	(+)	aKG-HExxH-type peptide beta-hydroxylase
orf2	ctg_589	609	202	(+)	hypothetical protein; putative methyltransferase
orf3	ctg_590	840	279	(+)	hypothetical protein
orf4	ctg_591	603	200	(+)	Thymidylate kinase
orf5	ctg_592	915	304	(+)	serine/threonine protein kinase
orf6	ctg_593	1662	553	(+)	aminotransferase class I/II-fold pyridoxal phosphate-dependent enzyme
orf7	ctg_594	948	315	(+)	Putative heavy-metal chelation
orf8	ctg_595	1119	372	(+)	nucleotidyltransferase
orf9	ctg_596	1593	530	(-)	Tryptophan halogenase
orf10	ctg_597	717	238	(-)	tryptophan 2,3-dioxygenase
orf11	ctg_598	849	282	(-)	Alpha/beta hydrolase family
orf12	ctg_599	987	328	(+)	Dioxygenase TauD/TfdAfdA
orf13	ctg_600	2358	785	(-)	pyridoxal-phosphate dependent enzyme; cysteine synthase
orf14	ctg_601	912	303	(-)	GHMP kinase
orf15	ctg_602	1530	509	(-)	argininosuccinate lyase/adenylosuccinate lyase
orf16	ctg_603	1641	546	(+)	sensor histidine kinase
orf17	ctg_604	7215	2404	(-)	gauD NRPS
orf18	ctg_605	17388	5795	(-)	gauC NRPS
orf19	ctg_606	3168	1055	(-)	gauB NRPS
orf20	ctg_607	213	70	(-)	MbtH-like protein

orf21	ctg_608	1347	448	(-)	ArsR/SmtB family transcription factor
orf22	ctg_609	804	267	(-)	ABC-2 family transporter protein
orf23	ctg_610	960	319	(-)	ABC transporter ATP-binding protein
orf24	ctg_611	657	218	(+)	LuxR family DNA-binding response regulator
orf25	ctg_612	1410	469	(-)	hypothetical protein
orf26	ctg_613	711	236	(-)	methyltransferase
orf27	ctg_614	1923	640	(-)	ABC transporter ATP-binding protein
orf28	ctg_615	1182	393	(-)	pyridoxal phosphate-dependent aminotransferase
orf29	ctg_616	954	317	(-)	Dioxygenase TauD/TfdA
orf30	ctg_617	1287	428	(-)	cytochrome P450
orf31	ctg_618	1890	629	(-)	partial NRPS D-Leucine specific Condensation/Adenylation domain
orf32	ctg_619	2430	809	(-)	partial NRPS D-Leucine specific PCP/Epimerisation/Condensation domain
orf33	ctg_620	17733	5910	(-)	gauA NRPS
orf34	ctg_621	3246	1081	(-)	β-Ala specific Condensation/Adenylation domain
orf35	ctg_622	264	87	(-)	phosphopantetheine-binding domain-containing protein
orf36	ctg_623	1749	582	(-)	acyl-CoA dehydrogenase
orf37	ctg_624	1707	568	(-)	acyl-CoA dehydrogenase
orf38	ctg_625	1815	604	(-)	fatty acyl-AMP ligase
orf39	ctg_626	903	300	(-)	winged helix-turn-helix transcriptional regulator
orf40	ctg_627	1056	351	(-)	glycosyltransferase
orf41	ctg_628	1185	394	(+)	2-isopropylmalate synthase
orf42	ctg_629	1395	464	(+)	leuC: 3-isopropylmalate dehydratase, large subunit
orf43	ctg_630	606	201	(+)	leuD: 3-isopropylmalate dehydratase, small subunit
orf44	ctg_631	1029	342	(+)	Isocitrate/isopropylmalate dehydrogenase

orf45	ctg_632	1233	410	(-)	diaminopimelate decarboxylase
orf46	ctg_633	1575	524	(-)	class I adenylate-forming enzyme family protein
orf47	ctg_634	291	96	(-)	PP-binding
orf48	ctg_635	1086	361	(+)	hypothetical protein
orf49	ctg_636	1167	388	(-)	Acyl-CoA dehydrogenase
orf50	ctg_637	975	324	(+)	NAD dependent epimerase/dehydratase family
orf51	ctg_638	198	65	(+)	IS3 family transposase
orf52	ctg_639	153	50	(+)	DUF1214 domain-containing protein
orf53	ctg_640	2655	884	(-)	LuxR family transcriptional regulator
orf54	ctg_641	480	159	(+)	DUF309 domain-containing protein
orf55	ctg_642	489	162	(+)	hypothetical protein
orf56	ctg_643	834	277	(-)	Sulfite exporter TauE/SafE

Table S10. Homologous ORFs in the gausemycin BGC of *Streptomyces* sp. INA-Ac-5812 and that of *Streptomyces* sp. B188M101.

**Gausemycin BGC from *Streptomyces* sp. INA-Ac-5812**				**Homologous ORFs in *Streptomyces* sp. B188M101 gausemycin BGC**	**% Identity**
orf1	QWT72257.1	1 - 1215 (+)	calcineurin		
orf2	QWT72258.1	1208 - 2257 (-)	alcohol dehydrogenase		
orf3	QWT72259.1	2422 - 3342 (+)	hypothetical protein		
orf4	QWT72260.1	3342 - 4274 (+)	Ku protein		
orf5	QWT72261.1	4366 - 4692 (-)	hypothetical protein		
orf6	QWT72262.1	4759 - 5772 (-)	SDR family oxidoreductase		
orf7	QWT72263.1	5833 - 7017 (-)	Zn-dependent alcohol dehydrogenase		
orf8	QWT72264.1	7341 - 7514 (-)	CsbD family protein		
orf9	QWT72265.1	7668 - 8651 (-)	hypothetical protein		
orf10	QWT72266.1	8815 - 9786 (-)	SDR family oxidoreductase	orf51 ctg1_638	89.9
orf11	QWT72267.1	10116 - 11279 (+)	acyl-CoA dehydrogenase	orf50 ctg1_637	89.2
orf12	QWT72268.1	11283 - 12209 (-)	hypothetical protein	orf49 ctg1_636	80.4
orf13	QWT72269.1	12206 - 13240 (-)	isocitrate/isopropylmalate dehydrogenase family protein	orf44 ctg1_631	97.1
orf14	QWT72270.1	13256 - 13861 (-)	3-isopropylmalate dehydratase small subunit	orf43 ctg1_630	95
orf15	QWT72271.1	13914 - 15308 (-)	3-isopropylmalate dehydratase large subunit	orf42 ctg1_629	96.6
orf16	QWT72272.1	15305 - 16489 (-)	pyruvate carboxyltransferase	orf41 ctg1_628	95.7
orf17	QWT72273.1	16730 - 17785 (+)	glycosyltransferase	orf40 ctg1_627	94.9
orf18	QWT72274.1	17994 - 18896 (+)	winged helix-turn-helix transcriptional regulator	orf39 ctg1_626<br>orf20 ctg1_607	92.3<br>38.2
orf19	QWT72275.1	18981 - 20795 (+)	fatty acyl-AMP ligase	orf38 ctg1_625	94.9
orf20	QWT72276.1	20792 - 22498 (+)	acyl-CoA dehydrogenase	orf37ctg1_624	93.8
orf21	QWT72277.1	22495 - 24240 (+)	acyl-CoA dehydrogenase	orf36 ctg1_623	93.6
orf22	QWT72278.1	24305 - 24568 (+)	hypothetical protein	orf35 ctg1_622	87.4
orf23	QWT72279.1	24626 - 49969 (+)	non-ribosomal peptide synthetase	orf16 ctg1_603<br>orf17 ctg1_604<br>orf31 ctg1_618<br>orf32 ctg1_619<br>orf33 ctg1_620<br>orf34 ctg1_621	43.5<br>34.6<br>90.6<br>95.9<br>93.4<br>93.6
orf24	QWT72280.1	50071 - 51357 (+)	cytochrome P450	orf30 ctg1_617	97.2
orf25	QWT72281.1	51382 - 52338 (+)	TauD/TfdA family dioxygenase	orf11 ctg1_598	38.1
orf26	QWT72282.1	52370 - 53551 (+)	pyridoxal phosphate-dependent aminotransferase	orf28 ctg1_615	92.9
orf27	QWT72283.1	53560 - 55482 (+)	ABC transporter ATP-binding protein	orf27 ctg1_614	96.9

orf28	QWT72284.1	55479 - 56933 (+)	hypothetical protein	orf25 ctg1_612	84.3
orf29	QWT72285.1	56954 - 57613 (-)	response regulator transcription factor	orf24 ctg1_611	94.5
orf30	QWT72286.1	57817 - 58776 (+)	ABC transporter ATP-binding protein	orf22 ctg1_609	99.1
orf31	QWT72287.1	58781 - 59584 (+)	ABC transporter permease	orf21 ctg1_608	95.5
orf32	QWT72288.1	60080 - 61081 (+)	winged helix-turn-helix transcriptional regulator	orf20 ctg1_607<br>orf39 ctg1_626	92.2<br>98.6
orf33	QWT72289.1	61111 - 61620 (-)	flavin reductase family protein		
orf34	QWT72290.1	61886 - 62098 (+)	MbtH family protein	orf19 ctg1_606	98.6
orf35	QWT72291.1	62280 - 65432 (+)	amino acid adenylation domain-containing protein	orf18 ctg1_605	93.9
orf36	QWT72292.1	65711 - 83146 (+)	putative non-ribosomal peptide synthetase	orf16 ctg1_603<br>orf17 ctg1_604<br>orf31 ctg1_618<br>orf32 ctg1_619<br>orf33 ctg1_620<br>orf34 ctg1_621	56.7<br>89.8<br>41.3<br>45.9<br>36.7<br>36.1
orf37	QWT72293.1	83143 - 90369 (+)	non-ribosomal peptide synthetase	orf16 ctg1_603<br>orf18 ctg1_605<br>orf32 ctg1_619<br>orf34 ctg1_621	92.4<br>31.9<br>46.2<br>35.1
orf38	QWT72294.1	90309 - 91949 (-)	histidine kinase	orf15 ctg1_602	92.3
orf39	QWT72295.1	92209 - 92424 (+)	argininosuccinate lyase		
orf40	QWT72296.1	92418 - 93329 (+)	kinase	orf13 ctg1_600	95.7
orf41	QWT72297.1	93402 - 94388 (-)	hypothetical protein	orf11 ctg1_598	93.3
orf42	QWT72298.1	94571 - 95419 (+)	alpha/beta hydrolase	orf10 ctg1_597	92.1
orf43	QWT72299.1	95416 - 96144 (+)	tryptophan 2,3-dioxygenase	orf9 ctg1_596	94.1
orf44	QWT72300.1	96457 - 98055 (+)	tryptophan 7-halogenase	orf8 ctg1_595	97.7
orf45	QWT72301.1	98077 - 99366 (+)	cation/H(+) antiporter		
orf46	QWT72302.1	99373 - 100626 (+)	decarboxylase	orf45 ctg1_632	68.8
orf47	QWT72303.1	100661 - 101122 (-)	NUDIX domain-containing protein		
orf48	QWT72304.1	101169 - 102692 (-)	long-chain fatty acid--CoA ligase	orf46 ctg1_633	45.3
orf49	QWT72305.1	102705 - 103757 (-)	aminoglycoside phosphotransferase family protein		
orf50	QWT72306.1	103754 - 104947 (-)	hypothetical protein		
orf51	QWT72307.1	105002 - 105643 (-)	hypothetical protein		
orf52	QWT72308.1	105669 - 106472 (-)	class I SAM-dependent methyltransferase		
orf53	QWT72309.1	106466 - 107470 (-)	hypothetical protein SMALA_6641		
orf54	QWT72310.1	107850 - 108836 (+)	aldo/keto reductase		
orf55	QWT72311.1	108899 - 109669 (+)	glucose 1-dehydrogenase		
orf56	QWT72312.1	109732 - 110808 (+)	myo-inositol-1-phosphate synthase		
orf57	QWT72313.1	110815 - 111618 (+)	histidinol-phosphatase		
orf58	QWT72314.1	111660 - 112877 (+)	S-adenosylmethionine synthetase		
orf59	QWT72315.1	112871 - 113893 (+)	hypothetical protein		
orf60	QWT72316.1	113890 - 114456 (+)	hypothetical protein		
orf61	QWT72317.1	114449 - 115726 (+)	DegT/DnrJ/EryC1/StrS family aminotransferase		
orf62	QWT72318.1	115741 - 116232 (+)	NUDIX domain-containing protein		
orf63	QWT72319.1	116262 - 116996 (-)	class I SAM-dependent methyltransferase		
orf64	QWT72320.1	117123 - 117446 (+)	hypothetical protein		
orf65	QWT72321.1	117512 - 118261 (+)	HAD hydrolase family protein		
orf66	QWT72322.1	118273 - 119295 (-)	UDP-glucose 4-epimerase		
orf67	QWT72323.1	119477 - 120388 (+)	hypothetical protein		
orf68	QWT72324.1	120388 - 121518 (+)	hypothetical protein		

Experimental procedure

Marfey's analysis

In sealed Eppendorf tubes, 150 µg of Compound 5 was dissolved in 100 µL of 6 M HCl and heated to 100 °C for 24 hours. After the hydrolysate was dried in a nitrogen drier at 40 °C, it was treated with 20 µL of 1 M NaHCO3 solution and 40 µL of 1% L-FDAA (Marfey's reagent, 1-fluoro-2-4-dinitrophenyl-5-ɣ-alanine amide) solution in acetone. Following one hour of gentle heating at 40 °C, the solutions were neutralised by adding 20 µL of 6 M HCl, diluted with 100 µL of acetonitrile, and centrifuged. The same previously reported procedure was used to derivatise the standard amino acids with L-FDAA. A Phenomenex C18 analytical column (5 µm, 4.6 × 150 mm) and an HPLC Agilent 1260 Infinity equipment were then used to receive the L-FDAA derivatised standard amino acids (2 µL supernatant) and the L-FDAA derivatised hydrolysates (2 µL supernatant) of compounds 1 and 2 at room temperature. A diode array HPLC detector at 340 nm monitored the linear gradient elution mode of the mobile phase solvents, which included a mixture of A (100% water, 0.1% FA) and B (100% acetonitrile, 0.1% FA). The elution mode was 10 to 65% CH3CN for 50 minutes and 65 to 100% CH3CN over 20 minutes at a flow rate of 1.0 mL/min.

Reference

(1) Cheng, C.; Othman, E. M.; Reimer, A.; Grüne, M.; Kozjak-Pavlovic, V.; Stopper, H.; Hentschel, U.; Abdelmohsen, U. R. Ageloline A, New Antioxidant and Antichlamydial Quinolone from the Marine Sponge-Derived Bacterium Streptomyces Sp. SBT345. *Tetrahedron Lett* **2016**, *57* (25), 2786–2789. https://doi.org/10.1016/j.tetlet.2016.05.042.

(2) *NMR Guidelines for ACS Journals*. https://pubsapp.acs.org/paragonplus/submission/acs_nmr_guidelines.pdf?
